# Improving Immunotherapy Through Glycodesign

**DOI:** 10.3389/fimmu.2018.02485

**Published:** 2018-11-02

**Authors:** Matthew J. Buettner, Sagar R. Shah, Christopher T. Saeui, Ryan Ariss, Kevin J. Yarema

**Affiliations:** ^1^Department of Biomedical Engineering and the Translational Tissue Engineering Center, The Johns Hopkins University, Baltimore, MD, United States; ^2^Pharmacology/Toxicology Branch I, Division of Clinical Evaluation and Pharmacology/Toxicology, Office of Tissues and Advanced Therapies, Center for Biologics Evaluation and Research, U.S. Food and Drug Administration, Bethesda, MD, United States

**Keywords:** immunotherapy, glycosylation, antibody-drug conjugates (ADCs), monoclonal antibodies, antibody-dependent cell cytotoxicity (ADCC), glycoengineering, metabolic glycoengineering

## Abstract

Immunotherapy is revolutionizing health care, with the majority of high impact “drugs” approved in the past decade falling into this category of therapy. Despite considerable success, glycosylation—a key design parameter that ensures safety, optimizes biological response, and influences the pharmacokinetic properties of an immunotherapeutic—has slowed the development of this class of drugs in the past and remains challenging at present. This article describes how optimizing glycosylation through a variety of glycoengineering strategies provides enticing opportunities to not only avoid past pitfalls, but also to substantially improve immunotherapies including antibodies and recombinant proteins, and cell-based therapies. We cover design principles important for early stage pre-clinical development and also discuss how various glycoengineering strategies can augment the biomanufacturing process to ensure the overall effectiveness of immunotherapeutics.

## Introduction

**Figure 1:**
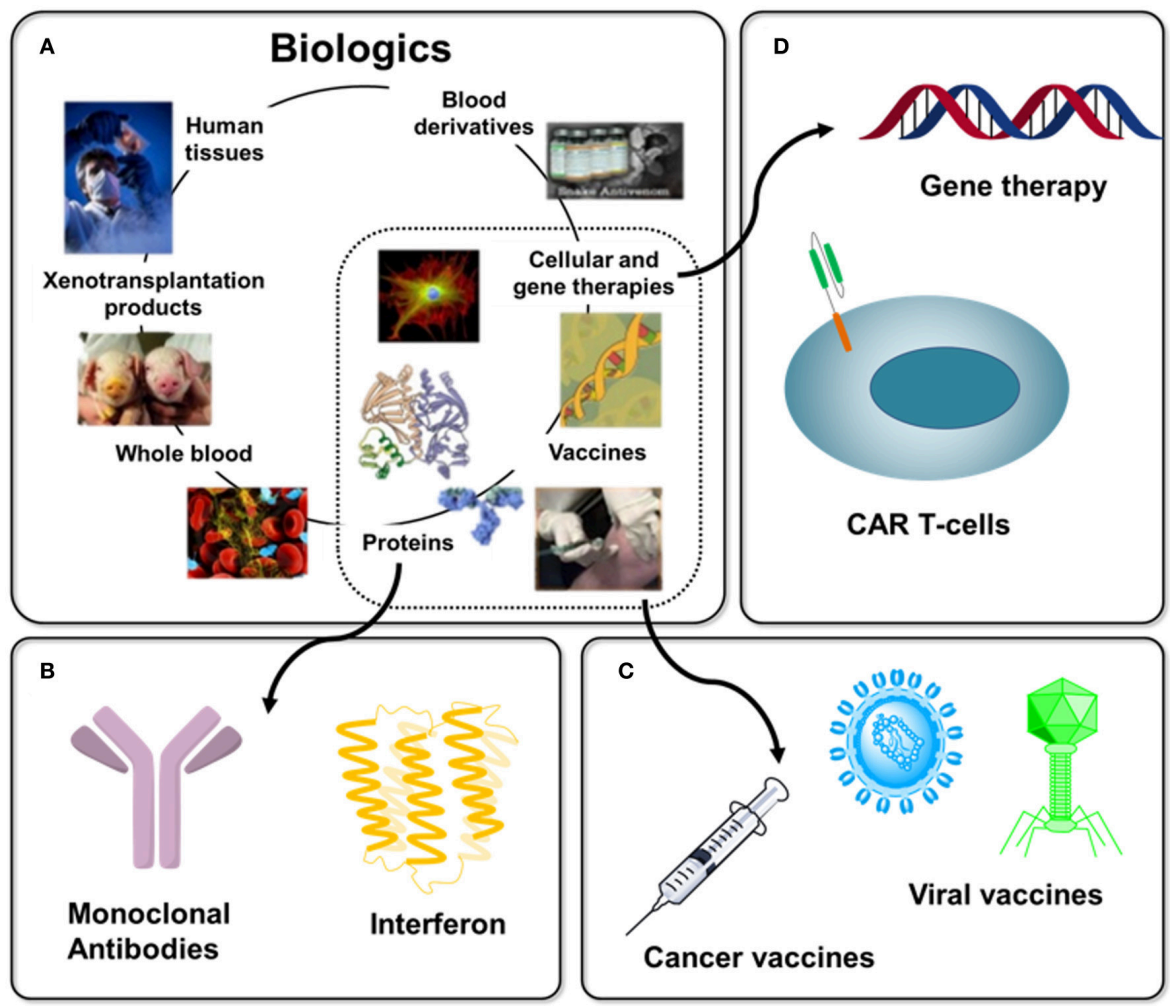
Overview of Biologics with immunotherapy-related examples. **(A)** “Biologics” is a broad term that refers to any therapy created using material derived from a living system, several examples are shown [as adapted from Chhina (8)]. **(B)** Protein-based biologics dominate today's commercial products with examples discussed in this article including monoclonal antibodies (section Antibodies) and interferon (section Blocking Antibodies). **(C)** Until a few decades ago, vaccines dominated immunotherapy, a 200-year old endeavor (section Vaccines), with cancer vaccines (section O-Glycans in Immunotherapy and 3.3) representing one example of this trend today. **(D)**. The extraordinarily diverse nature of immunotherapy is illustrated by emerging cell-based (e.g., CAR T-cell, section Chimeric Antigen Receptor (CAR) T-cell Therapy) and gene therapies.

Over the past 30 years immunotherapy, a term that encompasses any strategy that induces, enhances, or suppresses the body's natural immune system to treat disease, has emerged as today's preeminent approach to new drug development. In reality immunotherapy is a centuries-old technology, dating from Edward Jenner's discovery in 1796 that inoculation with fluid from cowpox lesions could protect against smallpox. Over the next ~200 years immunotherapy largely involved vaccine development until the advent of recombinant DNA technology in the 1970s and 1980s opened the door to today's impressive repertoire of immunotherapeutics, which include hormones, cytokines, antibodies, enzymes, and immune cells (1–6). The value of immunotherapeutics reached $107 billion (U.S. dollars) in 2017 with market projections soaring to $180 billion by 2025 (7); this strong projected growth indicates that many new immunotherapies are anticipated in the near future. This article describes how glycosylation is critical for the ongoing success of this important segment of today's burgeoning “biologics” drug market (Figure 1) by ensuring the safety and improving the function, activity, efficacy, physicochemical, and pharmacokinetic properties of immunotherapeutics (9–14).

To begin this article (next, in section The Role of Glycosylation in Immunotherapy), we provide an overview of mammalian glycosylation—with a focus on N-glycosylation—and highlight how specific glycans impact human immunity and then in section Glycodesign of Immunotherapeutics provide illustrative examples of how glycans modulate various types of immunotherapies. The sheer complexity and vast diversity of glycosylation makes quality control during the manufacturing of biologics a daunting task (15); we are confident, however, that various “glycoengineering” strategies, as outlined in section Design Considerations and Biomanufacturing, hold great promise for improving existing, and developing novel, immunotherapeutics.

## The role of glycosylation in immunotherapy

Historically, the central dogma of biochemistry was based on the belief that the flow of information from a DNA template to RNA to protein could unlock and predict underlying functional and evolutionary relationships in biology. In recent years this paradigm has shifted dramatically by emphasizing upstream epigenetic factors that control gene expression as well as downstream post-translational modifications (PTMs). This article focuses on glycosylation, a ubiquitous PTM in all three domains of life (archaea, bacteria, and eukarya); in mammals, carbohydrates can be divided into three primary types: N-linked glycans, O-linked glycans, and glycolipids (16). With the emergence of glycobiology in 1980s (17) and the realization that glycans modulate almost all aspects of human biology—especially the immune system [exemplified by the role of glycans in modulating the function of IgG antibodies (18), a topic discussed throughout this article]—the stage was set to apply lessons learned to the burgeoning field of immunotherapy. Here, in section The Role of Glycosylation in Immunotherapy, we briefly review mammalian glycosylation and its impact on immunotherapy; this focus stems from emerging dominance of mammalian systems as the predominant production platform for immunotherapeutics (6).

### N-glycans

N-Glycans are oligosaccharides covalently linked to the amide nitrogen of asparagine; they constitute one of the most common and almost certainly the most complex type of PTM (19, 20). Here we provide an overview of mammalian N-glycan biosynthesis [for more thorough information, see (19–22)] along with illustrative examples of how various N-glycans modulate immunity. In the next sub-sections we describe N-glycan biosynthesis in a step-by-step manner and highly salient features relevant to immunotherapy. This information provides a foundation for optimizing drugs—mostly biologics—used in immunotherapy (this class of drugs is referred to as “immunotherapeutics” in this paper).

#### Early steps in N-glycan biosynthesis

N-Glycan biosynthesis occurs in two distinct stages in the endoplasmic reticulum (ER) and the Golgi apparatus, respectively (19, 23). N-Glycan biosynthesis begins in the ER with the synthesis of the lipid-linked oligosaccharide (LLO) structure. Dolichol is an isoprenoid lipid that functions as an oligosaccharide carrier during early LLO synthesis on the cytosolic face of the ER membrane (19, 24, 25) where Man_5_GlcNAc_2_-P-P-dolichol is formed. This glycolipid is translocated into the ER lumen by a flippase (26, 27) where it is further elaborated to the final 14-mer LLO structure (Glc_3_Man_9_GlcNAc_2_-P-P-dolichol), which is transferred by an oligosaccharyltransferase to an asparagine residue in the consensus motif Asn-X-Ser/Thr of a nascent polypeptide chain during its translation across the ER membrane (28, 29).

#### N-glycan processing and structural diversification

**Figure 2:**
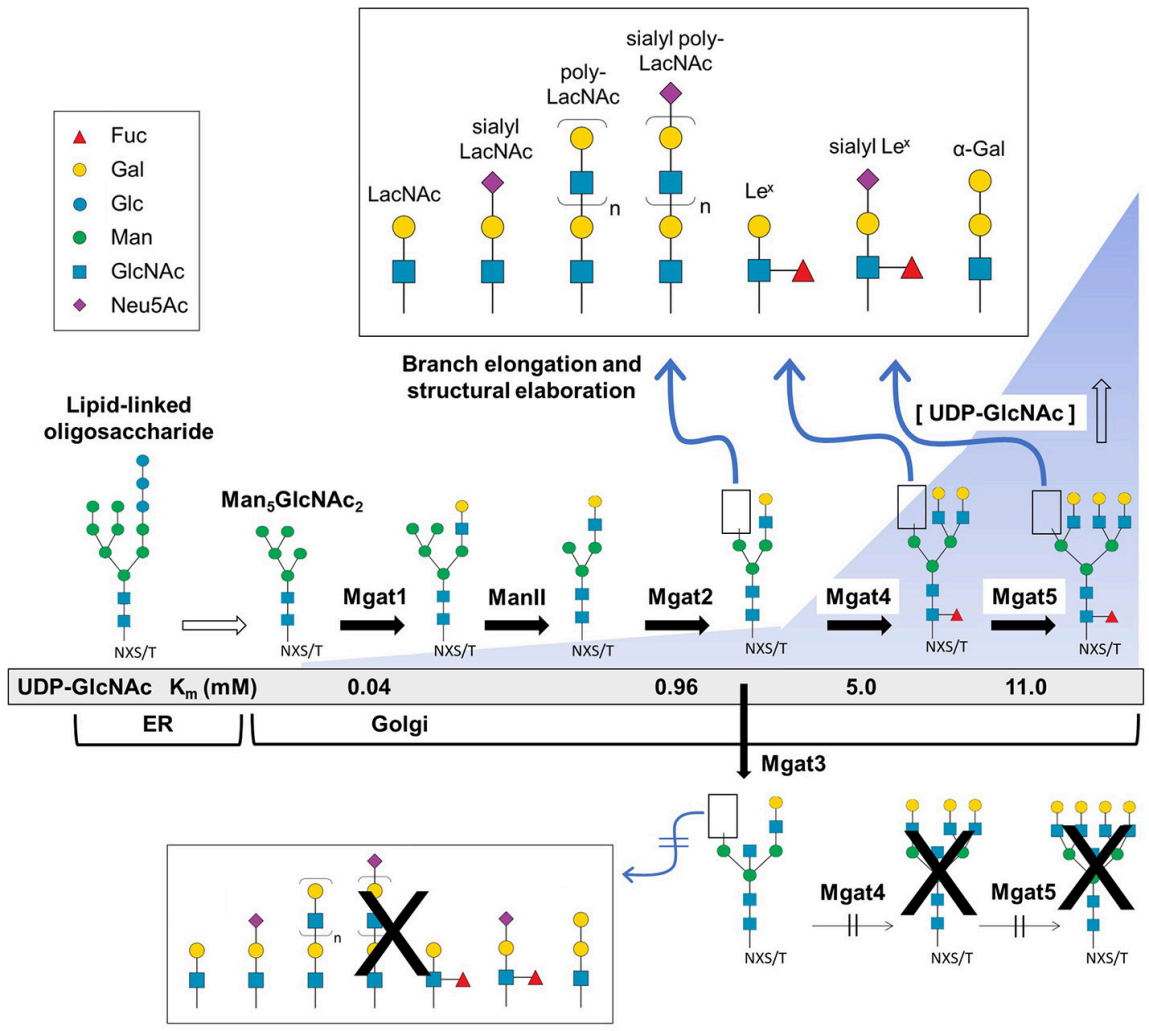
Branch elongation and structural diversity of N-glycans. The Glc_3_Man_9_GlcNAc_2_-P-P-dolichol LLO structure is synthesized in the ER where it is further processed and transferred to the Golgi resulting in high mannose (e.g., Man_5_GlcNAc_2_), hybrid, and complex type N-glycans that undergo branching via Mgat1, 2, 4, and 5 GlcNAc transferase activity that respectively creates di-, tri-, or tetra-antennary structures. Following the initial branching step, the glycan structure may be fucosylated or undergo additional elongation and capping modifications (Top panel). Alternatively, Mgat3 may add a bisecting GlcNAc residue which blocks Mgat4 and 5 activity thereby preventing tri- and tetra-antennary and further terminal diversification (bottom). The presence of a bisecting GlcNAc also hinders core fucosylation (red triangle) and reduces the capacity for downstream elongation and capping. [All glycan symbol structures in this figure and throughout this document were made using software from Cheng and coauthors (30)].

The second phase of N-glycan biosynthesis encompasses the processing of LLOs (as outlined in Figure 2) into three general categories (high mannose, hybrid, and complex) decorated with thousands of potential structural motifs (31–33) after transport of the host protein from the ER to the Golgi. This diversification of N-glycans—being a non-template based process—results in numerous and difficult-to-predict glycoforms. As described below, the sequential modification of mannose, GlcNAc, galactose, fucose, and sialic acid modulates many aspects of biology, including most aspects of immunotherapy (20).

##### Mannose

In the Golgi, a proportion of the Man_8/9_GlcNAc_2_ structures avoid further modification (beyond the cleavage of mannose residues to form Man_5−9_GlcNAc_2_) resulting in high mannose type N- glycans (19) that affect glycoprotein secretion, folding, and stability (34). For example, high mannose N-glycans can increase serum clearance and immunogenicity of IgG antibodies (35–37) although this is not always the case (38). High mannose N-glycans are associated with enhanced IgG monoclonal antibody (mAb) binding to FcγRIIIa and concomitant higher antibody-dependent cell cytotoxicity (ADCC) activity [ADCC is discussed in more detail in section Antibody-dependent Cell Cytotoxicity (ADCC)]. This effect was observed across the range of five to nine mannose residues (36, 37, 39–41) suggesting that enhanced activity could be due to a lack of core fucosylation (discussed below in section Fucose) and not the presence (or absence) of mannose *per se*. High mannose glycans with more than five mannose resides also lessen C1q (a vital receptor for complement dependent cytotoxicity [CDC]) binding, yielding diminished CDC activity (36, 39, 42).

##### Branching (Mgat1,2,4,5)

**Figure 3:**
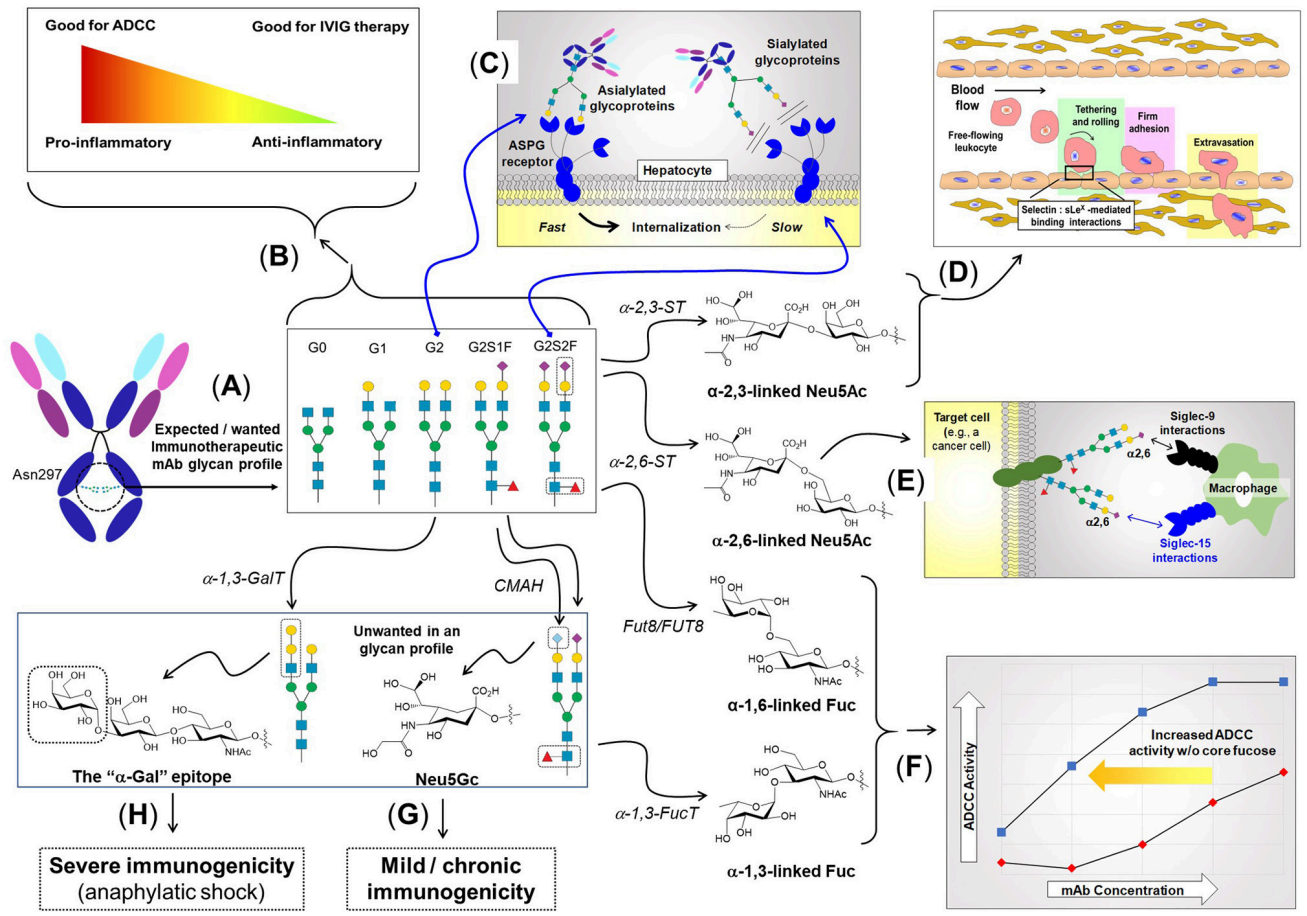
The role of N-linked glycosylation in mAb function and other aspects of immunity. **(A)** IgG type antibodies have two N-linked glycosylation sites at Asn297 of the Fc region that usually bear biantennary complex type N-glycans elongated with zero (G0), one (G1), or two (G2) galactose residues. **(B)** The presence of fucose and sialic acid inhibits FcγRIIIa binding resulting in lower ADCC activity; conversely, the anti-inflammatory character of sialic acid makes its presence desirable for IVIG therapy. **(C)** The presence (or absence) of sialic acid affects binding to the ASGP receptor, resulting in quick recycling of asialylated therapeutic proteins, which reduces serum half-life. By contrast, sialylation block ASGP receptor-mediated recycling, improving pharmacokinetic properties. **(D)** Neu5Ac added to galactose in an α2,3-linkage elicits a certain set of biological responses, one of which is—as part of the sLe^x^ epitope (shown in Figure 4)—to facilitate immune cell trafficking throughout the body by enabling “tethering and rolling” steps of leukocyte extravasation from the vascular system. **(E)** Neu5Ac in an α2,6-linkage elicits a distinct set of response, including binding to Siglec receptors (45), where in the example shown, adapted from Büll et al. (46), this moiety modulates macrophage activity. **(F)** Core fucose, in particular in the α1,6-linkage, inhibits ADCC requiring higher mAb antibodies compared to defucosylation drug [adapted from GlycoWord (47)]. Glycans can also result in unwanted immunogenicity ranging from mild, chronic responses emanating from Neu5Gc **(G)** (48), to life-threatening, anaphylactic responses from α-Gal **(H)** (49).

In most cases, high mannose type N-glycans are further processed in the Golgi resulting in hybrid- and complex-type N-glycans (Figure 2). The process of N-glycan branching and elongation begins in the *medial*-Golgi with the transfer of GlcNAc to the Man_5_GlcNAc_2_ structure by N-acetylglucosaminyltransferase, Mgat1 (43). For hybrid N-glycans, the high mannose branch remains unaltered while the branch ending in GlcNAc is usually further elongated with galactose and GlcNAc or capped with sialic acid, or fucose, as described below. Complex type N-glycans have two additional mannose residues cleaved by α-mannosidases (Man2a1 or Man2a2) to produce GlcNAcMan_3_GlcNAc_2_ (44), which is elaborated with bi- (and sometimes tri-, and tetra-) antennary branches by the sequential addition of GlcNAc residues via Mgat2, Mgat4, and Mgat5. The GlcNAc transferases have decreasing affinity (higher K_m_ values) for the substrate UDP-GlcNAc creating an ultrasensitive cascade (Figure 2) that usually limits branching to bi-antennary structures (e.g., as shown in Figure 3 for a typical IgG mAb) (43, 50).

N-Glycan branching plays numerous roles in regulating the immune system ranging from T-cell activation (38, 51), autoimmunity (38, 51), cytokine production (52), cancer metastasis (53), to cell proliferation and differentiation (54). From an immunotherapy perspective, N-glycan branching influences the physicochemical properties and the metabolic turnover of immunotherapeutics by modulating the overall charge, isoelectric point, size, and valence of these molecules; more specifically increased branching provides more sites for sialylation giving the glycoprotein a higher negative charge (55) that impacts physicochemical properties (see section Design Considerations and Biomanufacturing). The serum half-life of immunotherapeutics also is influenced by terminal sialylation, which masks the penultimate galactose moiety from the hepatocyte asialoglycoprotein (ASGP) receptor (Figure 3C), reducing glomeruli clearance in the kidneys (56, 57).

##### Bisecting GlcNAc (Mgat3)

The discerning reader may have noted the curious omission of Mgat3 from the previous paragraph; the reason is that this enzyme is an outlier that counteracts several aspects of N-glycan diversification and elongation. Specifically, Mgat3-catalyzed addition of GlcNAc to the β-mannose of an N-glycan in a bisecting orientation (53, 58) inhibits the activity of Mgat4 and Mgat5 negating tri- and tetra-antennary branching (and subsequent elongation of the resultant antennary branches) and also reduces core fucosylation (Figure 2) (41, 43, 59). Although only a single monosaccharide, the ability of bisecting GlcNAc to block subsequent branching and core fucosylation has a disproportional impact on overall N-glycan structure and bioactivity [e.g., in cancer metastasis (60–63), apolipoprotein B function (64) and the epithelial-mesenchymal transition (65, 66)].

The potent ability of bisecting GlcNAc to modulate biological activity makes this monosaccharide a crucial design parameter in immunotherapy. For example, bisecting GlcNAc blocks tri- and tetra-antennary N-glycan branching, which limits the number of potential sites for sialylation on a glycoprotein thereby reducing serum half-life and altering the physicochemical properties (sialylation is further discussed in section Sialic Acid). Similarly, limiting N-glycan branching alters the overall structure and composition of glycoproteins which has numerous implications for surface charge, hydrophobicity and colloidal/conformation stability, which is discussed further in section Physicochemical Properties Mgat3 inhibits α(2,3)-sialylation, which can reduce terminal sialylation or alternately, enhance α(2,6)-sialylation (67) (Figure 3). The presence of a bisecting GlcNAc in Fc region N-glycans in IgG antibodies increases binding affinity to FcγRIIIa leading to a 10-20 fold increase in antibody dependent cell cytotoxicity (68); which is consistent with the loss of core fucosylation that can increase ADCC activity by up to ~100-fold (69–71). Finally, Mgat3 impedes synthesis of galactose-α(1,3)-galactose (α-Gal, Figure 3), an epitope that can elicit severely-deleterious immunogenic responses (49, 72).

##### Galactose

After GlcNAc has been added to a nascent N-glycan to form hybrid or complex structures, this moiety is commonly elongated with galactose by a β(1,4)-galactosyltransferase, which creates the Galβ(1-4)GlcNAc unit known as “LacNAc” (73, 74). Additional galactose residues may be added by β(1,4)- or α(1,3)-galactosyltransferases, either consecutively or interspersed with other monosaccharides (e.g., GlcNAc) to create a variety of N-glycan structures (Figure 2). Although terminal galactose has minimal influence on ADCC activity or the pharmacological properties of recombinant IgGs (75, 76), it can nonetheless impact the efficacy of various therapeutic mAbs (41, 77); for example, increases in heavy chain galactose content can increase CDC in rituximab (78) and alemtuzumab (79). Although generally modest, galactose-dependent CDC has led regulatory bodies to require strict monitoring of galactosylation patterns of immunotherapeutics (and other biologics) with terminal galactose groups (G0, G1, or G2, Figure 3) now a major quality control parameter in the biomanufacturing industry (77, 80, 81).

Galactose linked to an underlying galactose via an α(1,3)-linkage constitutes the α-Gal epitope, which can have widespread ramifications for the safety, efficacy, and pharmacokinetic properties of immunotherapeutics. The α-Gal epitope is common in non-primate mammals but is absent in humans; as a result people have circulating antibodies against this antigen, which led to severe immunogenic responses, and even patient deaths, in early immunotherapy trials in 2004 (49, 82, 83). Sequential addition of GlcNAc in conjunction with galactose produces LacNAc units that often are added preferentially to a specific N-glycan branch resulting in structural asymmetry that impacts function and biological recognitionthat, in one example, affects the immunomodulatory properties of milk oligosaccharides through tuning interactions with both pathogens and glycan binding proteins such as galectin (84).

##### Fucose

**Figure 4:**
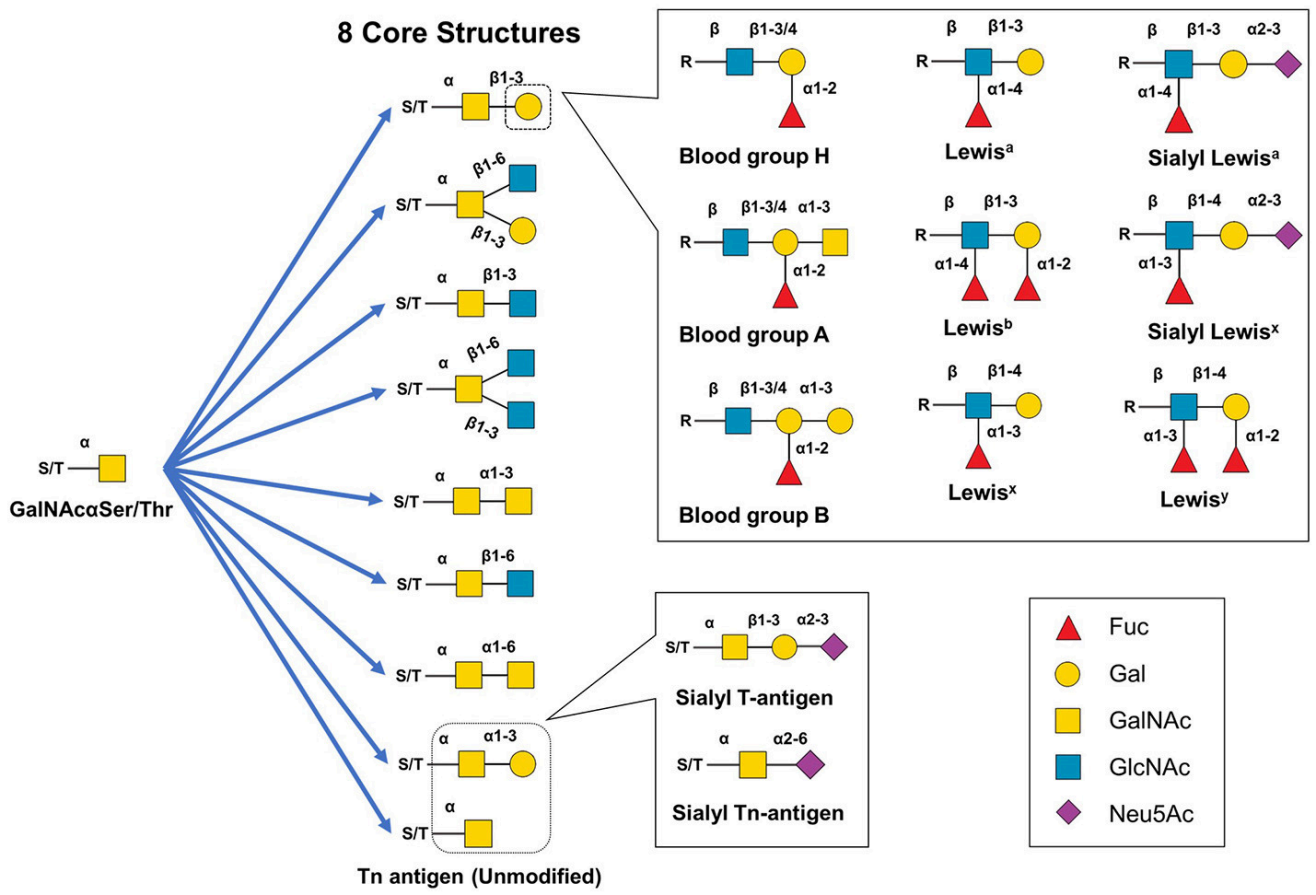
Structural diversity of mucin-type O-glycans. Mucin type O-glycan biosynthesis begins with the transfer of GalNAc to serine or threonine. The GalNAc monosaccharaide can be left unmodified but is typically extended to create eight different core structures that can be further modified with single monosaccharides, Lewis structural epitopes, blood group antigen groups, or other glycan epitopes (e.g., the cancer-related sT or sTn antigens).

Hybrid and complex type N-glycan branches often end with GlcNAc or galactose but can also be decorated with fucose (this section) or terminally capped with sialic acids, meaning that typically once these sugars are added, the oligosaccharide chain cannot be further elongated (section Sialic Acid, below). Fucose is a prevalent modification of the complex type N-glycans; in humans fucosyltransferases add this sugar in an α(1,2) (FUT1,2), α(1,3/4) (FUT3-7,9), or α(1,6) (FUT8) orientation; in mammals, Fut8 adds a fucose residue exclusively to the innermost Asn-linked GlcNAc group (*a.k.a*., “core” fucosylation). Fucose can also be added as a capping moiety to an outermost galactose by Fut1,2 forming Lewis and blood group antigens (85, 86) (see Figure 4).

Core α(1-6) fucose has widespread biological activity ranging from modulating growth factors (87–89) and to affecting the incidence and progression of cancer (90–94) while *Fut8*-null mice display multiple phenotypes including semi-lethality, the development of emphysema, brain dysfunction, and impaired immunity (58). Based on the many biological and physiological roles of core fucosylation, it is not surprising that this sugar plays integral roles in immunotherapy; for example, core fucosylation inhibits IgG binding to FcγRIIIa thereby decreasing ADCC activity (41, 70, 71, 95–105). Conversely, defucosylation of clinically-used mAbs including rituximab, trastuzumab, and pertuzumab can increase ADCC activity up to two-fold (70, 71, 101, 105). Another wrinkle of core fucosylation is that α(1,3)-fucosylation—which is prevalent in plant cells including those under consideration for biomanufacturing (106)—can impact mammalian immunity [e.g., through Fc receptor interactions (107)]; as a result, the use of plant hosts for biomanufacturing is proceeding cautiously.

##### Sialic acid

Sialic acids – a family α-keto acids comprised of a nine carbon backbone with over 50 different variants—ubiquitously cap glycans (19, 20, 108). N-Acetylneuraminic acid (Neu5Ac) is the predominant sialic acid in humans and is typically found at the termini of N-glycan branches where it is added to the penultimate galactose via α(2,3)-, α(2,6)-, or less commonly, α(2,8)-sialyltransferases (109, 110). Depending on its linkage [e.g., α(2,3)- vs. α(2,6)-] sialic acid exhibits numerous biological functions in nervous system embryogenesis, cancer metastasis, immune responses, and protein bioactivity and stability (110, 111).

Relevant to therapeutics, sialic acid increases the serum half-life of numerous recombinant glycoproteins including erythropoietin (EPO), interferon γ, interferon α, IgG antibodies, and serum albumin (12) by masking the terminal galactose and GlcNAc residues from the hepatocyte ASGP receptor and thus preventing endocytosis to prolong circulatory lifetime (12, 57, 112). Furthermore, the negative charge of sialic acid reduces proteolytic degradation and kidney clearance (12, 113, 114) due to its impact on physicochemical properties. Finally, sialylation (along with fucose) can tune the immunogenicity of antibodies (Figure 3) resulting in contrasting effects illustrated by ADCC and intravenous immunoglobin (IVIG) therapy. Sialylation of IgG interferes with FcγRIIIa binding reducing ADCC activity in mouse hybridoma lines (41, 76); conversely, this immunosuppressive activity is critical for IVIG therapy (see section Intravenous Immunoglobulin (IVIG) Therapy). Mechanistically, suppression of inflammation is linked to the C-type lectin receptor-specific intracellular adhesion molecule-grabbing nonintegrin R1 (SIGN-R1 or DC-SIGN in humans), which requires IgG ligands with sialylated Fc glycans (115–117).

Another example of a coordinated function of sialic acid and fucose is provided by sialyl Lewis x (sLe^x^) (86) where both sugars are required for selectin-mediated immune cell trafficking (section Mesenchymal Stem Cell (MSC) Homing). The mechanism for homing relies on the selectin family comprised of E-selectin (CD62E), L-selctin (CD62L), and P-selectin (CD62P) which bind to a sialofucosylated epitope, namely sLe^x^, in a Ca^+2^-dependent manner. The sLe^x^ epitope is vital for both naïve T-cell and activated T effector cell homing to various tissues (118).

### O-glycans

O-Glycans are monosaccharides or oligosaccharides covalently linked to serine or threonine. Similar to N-glycans, O-glycan synthesis is not template-based and is defined by a vast array of possible structural permutations that play many biological and pathological roles including: protein stability, structure, folding, activity, metabolism, cell signaling, cell-cell interactions, and oncogenesis (119–122). This section focuses on mucin type O-glycans and how this category of O-linked glycosylation impacts immunotherapeutics.

#### Mucin type O-glycans

Although there are several types of O-glycans including O-linked GlcNAc, O-linked glucose, and O-linked fucose (120, 122) this article focuses on mucin-type O-glycans because of their relevance to immunotherapeutics. Mucin-type O-glycans, so named because of their abundance in mucins (and their initial isolation and characterization from mucus), are defined by having a GalNAc at the reducing terminus (119). Biosynthesis of mucin-type O-glycans begins in the Golgi with the transfer of GalNAc to a Ser or Thr residue by one of ~22 GalNAc transferases (123–125). While possible, a single unextended GalNAc (Tn antigen) is uncommon, instead various glycosyltransferases generate one of eight core structures (121, 122) (Figure 4A). These core structures can be further elongated and capped (generally with GlcNAc, Gal, sialic acid, fucose) to create numerous motifs such as the Lewis antigens (e.g., Le^y^, Le^x^, sLe^x^, Le^a^, sLe^a^, Le^b^) thereby substantially increasing structural diversity (119, 122, 126). Mucin-type O-glycans are involved in many biological functions including fertilization, signal transduction, cell structure, adhesion, homing, glycoprotein clearance, stability, and of course, immunity (119, 122).

#### O-glycans in immunotherapy

**Figure 5:**
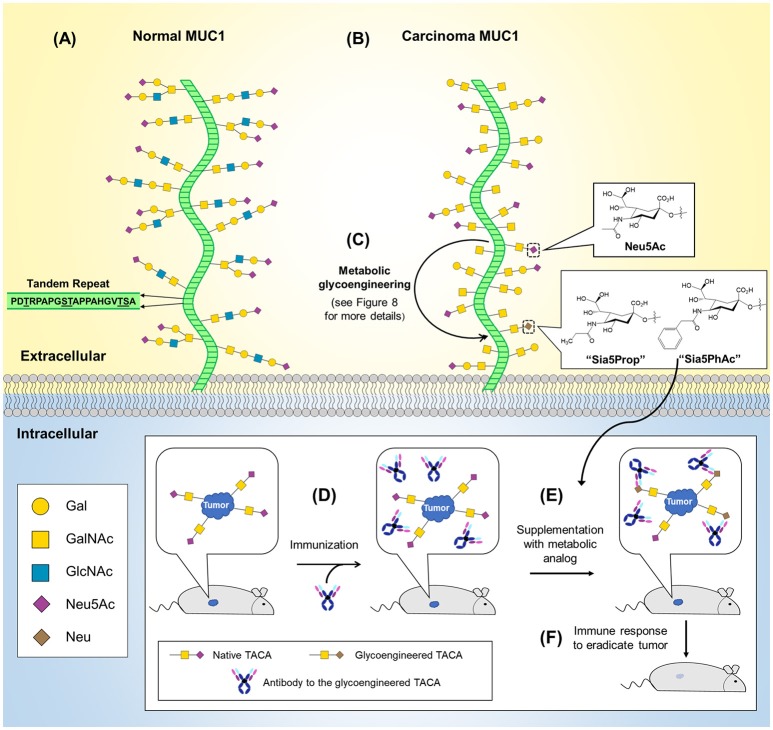
O-Glycans in normal and cancerous MUC1 and MUC1-based cancer vaccine development. **(A)** The MUC1 protein core (green) is composed of a 20 amino acid tandem repeat with each unit having five potential O-glycosylation sites. **(B)** MUC1 is overexpressed in numerous cancers (not shown) and is characterized by truncated O-glycans (shown). **(C)** MGE can be used to introduce non-natural chemical moieties (e.g., Sia5Prop and Sia5PhAc) to enhance the immunogenicity of tumor-associated cancer antigens (TACAs). As shown in the inset (bottom), antibodies can be developed to the glycoengineered TACAs and used to immunize a tumor-bearing animal **(D)**. **(E)** Supplementation with the MGE analog induces expression of the non-natural version of the TACA, resulting in tumor-selective binding and stimulation of the immune system to recognize and eradicate the tumor **(F)**.

An early example of O-glycosylation in immunotherapy is provided by mucin 1 (MUC1), a transmembrane glycoprotein overexpressed and abnormally glycosylated with Tn and sialyl Tn antigen in adenocarcinomas, squamous cell carcinomas, and myelomas making it a broad based cancer biomarker (127–129). Astonishingly, in 1999 it was estimated that cancers with aberrant MUC1 expression accounted for 72% of new cases and 66% of deaths in all cancers (130). The widespread occurrence of MUC1 across multiple types of cancer has made it a popular immunotherapy target with 16 new trials initiated in 2017 alone (127). Interest in MUC1-based cancer immunotherapy stems from this marker's aberrant glycosylation in tumor cells due to truncated, highly sialylated O-glycans that occur at up to five potential sites on each of MUC1's 20 amino acid tandem repeat sequence (Figure 5A). MUC1-targeting immunotherapies fall into three general categories vaccines, mAbs, and adoptive cell therapies. First, vaccines based on several different MUC1 antigens, such as synthetic peptides or MUC1 endogenously expressed by plasmid, synthetic mRNA, or viral vectors are now being tested (127–129, 131). An especially intriguing “cancer vaccine” approach to MUC1 employs metabolic glycoengineering strategies (a technology described in more detail in section Metabolic Glycoengineering) that incorporate non-natural sialic acids into glycan structures that increase their immunogenicity [as shown in Figure 5B and described in a series of papers primarily from the Guo group (132–135)]. In another approach, murine anti-MUC1 antibodies (muHMFG-1, mAB-AR20.5) and humanized anti-MUC1 antibodies (hPAM4, AS1402) are being evaluated in clinical trials (128, 136). Finally, autologous dendritic cells engineered to contain MUC1 as a peptide, mRNA or fused tumor cells have been designed to elicit immune-based antitumoral cytotoxicity (137–139) and most recently, chimeric antigen receptor (CAR) T-cells have been engineered to target MUC1 and the Tn antigen with 10 current phase I/II trials targeting MUC1 (127, 140–143).

In contrast to robust efforts to exploit O-glycans in immunotherapy, as just illustrated by MUC1, O-glycans largely have been overlooked as a design parameter in the biomanufacturing of immunotherapeutics; indeed, until a few years ago human IgGs—the largest class of immunotherapeutics—were not thought to contain O-glycans. It is now known, however, that the hinge region of several classes of human immunoglobins including IgA (144–146), IgD (147, 148), as well as IgG (149, 150) have potential O-glycosylation sites. Specifically, IgA1 has nine potential O-glycosylation sites with three to five typically occupied (146, 150); IgD has six potential sites (148, 151); and human IgG has three potential sites with occupancies between 10 and 13% for IgG3 (150). Although relatively little is known about how O-glycosylation modulates the activity, specificity, or stability of mAbs it has been shown O-glycosylation plays an important role in Fc-fusion protein serum longevity. Notably, increased sialylation of the O-glycans of etanercept (tumor necrosis factor α receptor II-Fc-fusion) and BR3-Fc fusion enhance serum half-life (152, 153). Similar to N- glycans, this effect is attributed to sialic acid's ability to mask galactose from ASGP receptors preventing degradation in the liver (41). In the future, as the biological implications of mAb O-linked glycosylation are uncovered, the biomanufacturing industry (section Design Considerations and Biomanufacturing) likely will focus additional effort on controlling mucin-type O-glycosylation. At present O-glycans nevertheless provide an attractive “chemical handle” for conjugation reactions to improve glycoprotein pharmacokinetics. For example, GalNAc-transferases have been used to modify recombinantly-produced proteins with polyethylene glycol (PEG), a technology termed GlycoPEGylation (154). Covalently attaching PEG to recombinant proteins can augment serum half-life, pharmacokinetic and pharmacodynamic properties. Typically, recombinant proteins are PEGylated through amino acid residues, however it is vital to avoid conjugating PEG to amino acids in or near an active site or, for mAbs, near the antigen recognition domain (155). This issue can be circumvented by targeting O-glycans, which are usually located away from an active site (156, 157). GlycoPEGylation is predominantly used for recombinant therapeutic proteins expressed in *Escherichia coli* that lack endogenous mucin-type O-glycosylation and occurs in two general steps: (i) GalNAc-transferase adds a GalNAc to a Ser/Thr residue and (ii) CMP-Neu5Ac with covalently-attached PEG is added by a sialyltransferase. This technology has been employed for two clinically approved biologics: granulocyte/macrophage colony stimulating factor, and interferon-α2b (154, 158).

### Glycolipids

**Figure 6:**
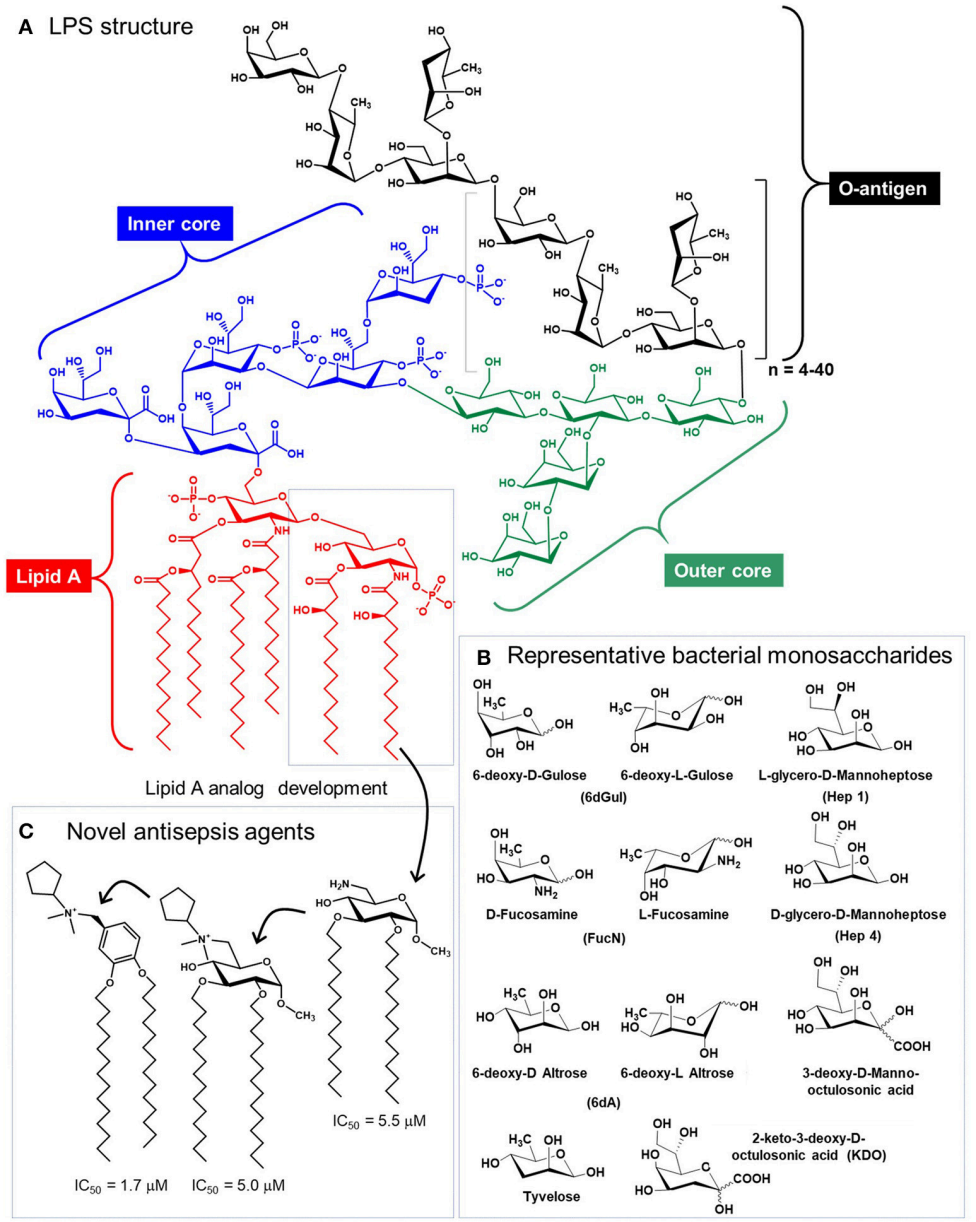
Structure of lipopolysaccharide (LPS). **(A)** Glycolipids, exemplified by bacterial structures such as LPS contain the Lipid A, and inner core, an outer core, and the O-antigen, which varies based on species and strain [*Salmonella enterica* Serotype Typhi is show (160)]. **(B)** LPS glycans contains a variety of non-mammalian monosaccharides, which contributes to their immunogenicity and provokes sepsis [**(A,B)** are adapted from Saeui et al. (161)]. **(C)** Medicinal chemistry efforts have exploited the Lipid A structure to create anti-inflammatory analogs [three are shown, from Piazza et al. (162)] that are promising anti-sepsis agents.

Glycolipids—a third major class of glycans—are perhaps an unlikely candidate for immunotherapy considering their longstanding role in provoking severe, detrimental immune responses (e.g., sepsis) that remains an increasing source of mortality in American hospitals (159). Sepsis is triggered by highly-immunogenic, microbe-derived Lipid-A-linked oligo- or polysaccharides that typically contain non-mammalian monosaccharides (Figure 6) (163). Interestingly, in 2009 Piazza and coworkers were able to rationally design glyco- and a benzylammonium-modified lipids that function as lipid-A antagonists and inhibit lipopolysaccharide-induced septic shock *in vivo* (162). This class of molecules provides a “small molecule” example of an immunotherapeutic that mimics IgG antibodies in that the compound's inherent immunomodulatory ability can be tuned up or down by chemical structural modifications. Since then, “immunopharmacy” efforts have continued to develop lipid A variants for vaccines and other therapies, as summarized by Wang and coauthors (164).

**Figure 7:**
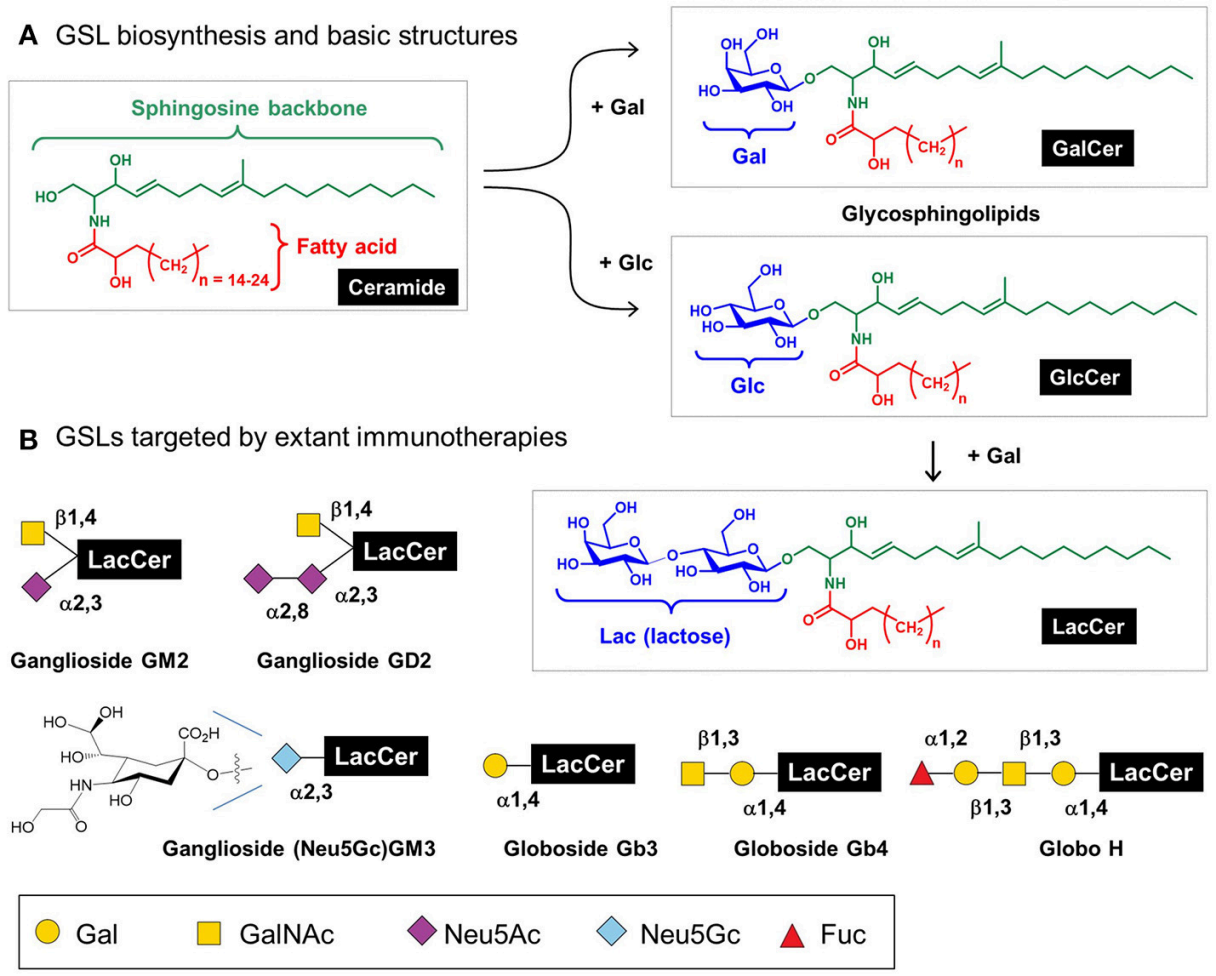
Glycosphingolipids (GSL) structures and role in immunotherapy. **(A)** Human GSLs are derived from ceramide upon addition of galactose (to form “GalCer”) or, more commonly, addition of glucose (to form “GlcCer”); a fraction of GlcCer is further elaborated with galactose to form “LacCer,” which is the building block for lacto(neo)series, globosides, and gangliosides as cataloged elsewhere (21); here [in **(B)**] we show several GSLs currently targeted by immunotherapy.

Mammalian glycosphingolipids (GSLs), comprised of a sphingolipid, fatty acid, and carbohydrate (Figure 7) provide another example of immunotherapy. GSLs are part of the cell membrane with various biological functions including cellular adhesion, cell-cell interactions, signal transduction, oncogenesis, ontogenesis, and immunogenicity (165–167). To date, efforts to exploit GSLs in immunotherapy have focused on cancer; these molecules are aberrantly expressed in a variety of cancers including breast, lung, colorectal, melanoma, prostate, ovarian, leukemia, renal, bladder, and gastric thereby constituting attractive broad-based diagnostic biomarkers and providing potential targets for cancer immunotherapy (168). Notably, multiple antibodies are in preclinical and clinical trials that target GSLs including GD2 (169), GM2 (170), Neu5GcGM3 (171), Gb3, Gb4, and Globo H (172). Another GSL, α-GalCer, has potential anti-tumor activity and is currently in phase 1 clinical trials in high risk melanoma patients (173).

Finally, from the perspective of the production of immunotherapeutic products, inhibition of GSL biosynthesis in Chinese hamster ovary (CHO) cells can enhance sialylation; for example, repressing the GSL biosynthetic enzyme UDP-glucose ceramide glucosyltranferase increased recombinant EPO sialylation. Interestingly, GSL inhibition did not change CMP-Neu5Ac levels in the Golgi or cytoplasm, suggesting that CMP-Neu5Ac was diverted to EPO sialylation as part of a dynamic equilibrium between GSL and N-glycan biosynthesis (174). Overall, this study provides an option for modulating GSL biosynthesis as a glycoengineering strategy to produce glycoproteins with favorable glycoforms.

## Glycodesign of immunotherapeutics

Over the past 30 years immunotherapy has moved from a focus on vaccines to encompass a diverse array of treatments with glycosylation now firmly established as a key parameter in the design, development, and production of virtually all types of immunotherapeutics. Here, we describe specific examples of how glycosylation impacts and modulates the efficacy of antibody-, recombinant protein-, and cell-based therapies while highlighting glycoengineering techniques that can ameliorate problems (e.g., safety) and enhance bioactivity and pharmacokinetics during the development and manufacturing of immunotherapeutics.

### Antibodies

Antibodies' ligand-specific targeting and their ability to elicit downstream effector functions (175) have established them as one of the largest classes of biologics overall and as the dominant commercial immunotherapeutic. As described in the following sub-sections, these versatile immunotherapeutics fall into several—often overlapping but sometimes very distinct—categories; several of these categories are summarized with a focus on the role of glycosylation.

#### Blocking antibodies

Blocking antibodies, as their name implies, are designed to bind to a biological target and by doing so, diminish its activity; for example, Cetuximab (a.k.a., Erbitux)—a pioneering cancer immunotherapeutic from ~20 years ago—blocks epidermal growth factor receptor activation and downstream oncogenic signaling (176–178). Interestingly, this early immunotherapeutic alerted the biomedical community to the importance of glycans when several patients suffered severe immune reactions to the α-Gal epitope (Figure 3) (49). As an aside, this unfortunate incident provided impetus for the subsequent transition of almost all recombinant mAb production to CHO cells (discussed in more detail in section Chinese Hamster Ovary (CHO) Cells) (6, 179, 180). Despite these early setbacks, interest in blocking antibodies remains strong with the programmed death ligand-1 (PDL1) providing a recent high-profile example. PDL1 is a transmembrane protein [which is glycosylated itself (181)] that binds to the programmed cell death protein-1 (PD1) thereby inhibiting T lymphocyte proliferation and cytolytic activity, immune suppression, and cytokine production (181). PDL1-blocking antibodies alleviate these inhibitory PDL1/PD1 interactions and reactivate T-cells to fight cancer (181, 182) with promising results against both leukemias and solid tumors (183). One recent study developed a mAb targeting glycosylated PDL1 in triple negative breast cancer cells which blocks PDL1/PD1 interactions and enhances PDL1 internalization and degradation. Furthermore, conjugating the anti-mitotic drug monomethyl auristatin E to this mAb resulted in significant cytotoxicity to cancer cells expressing glycosylated PDL1 with limited host toxicity (184).

#### Antibody-dependent cell cytotoxicity (ADCC)

ADCC is a cell-mediated immune defense where effector cells (typically natural killer cells but also macrophages, neutrophils, and eosinophils) actively lyse a target cell whose membrane-surface antigens have been bound by specific antibodies (185). In immunotherapy, antibodies are designed to selectively coat cancer cells, targeting them for eradication by Fc receptor effector cells (186). ADCC can be improved (or hindered) by glycosylation as illustrated by the glycosylation profiles of anti-HIV monoclonal antibodies (187) and the role of fucose and sialic acid in ADCC, as outlined by Ravetch and coauthors (101, 102, 188, 189); the “take home” message is that sialylation and core fucosylation generally inhibit ADCC, positioning simpler N-glycans that lack sialic acid, and especially fucose (e.g., as shown in Figure 3) as ideal glycoforms for antibodies designed to elicit ADCC. Interestingly, certain mAbs intended to block biological activity (section Blocking Antibodies) also elicit ADCC thus doubly benefitting cancer immunotherapy; indeed, the pioneering drug Cetuximab fits this criteria (190, 191).

#### Intravenous immunoglobulin (IVIG) therapy

In contrast to ADCC where sialic acid is unwanted, this sugar is critical for immunosuppression as illustrated by IVIG therapy, which is used to treat a wide range of autoimmune, infectious, and inflammatory diseases (115, 188, 192–194). In IVIG therapy, patients are dosed with concentrated IgG collected from pooled plasma (195). Although sialylation is not the sole determinant of the anti-inflammatory response underlying IVIG therapy (194), efficacy is enhanced by sialic acid (188). Because only ~10% of IgG Fc glycans are sialylated (with just 1–3% disialylated), very high doses (e.g., 1–2 g/kg) of IgG are required for IVIG therapy (9, 188, 196). A study by Washburn et al. where tetra-Fc sialylation of recombinant human IgG1 was achieved by the enzymatic addition of sialic acid showed up to ~10-fold higher anti-inflammatory activity than unsialylated IVIG across multiple animal models (18, 194).

#### Antibody drug conjugates (ADCs)

**Figure 8:**
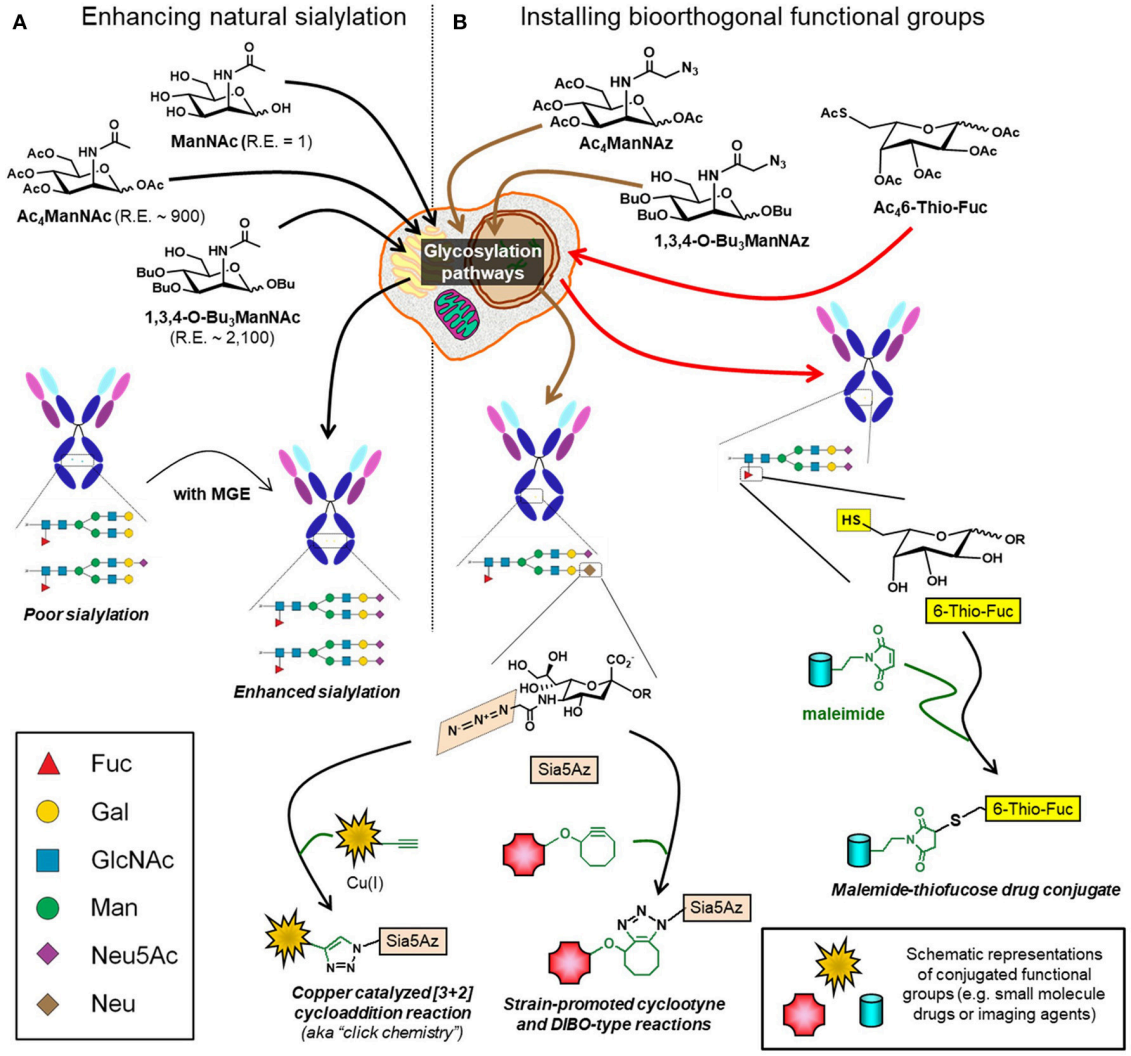
Glycoengineering mAbs for enhanced sialylation and glycan-targeted ADC production. **(A)** Cells can be supplemented with ManNAc or analogs (e.g., Ac_4_ManNAc or 1,3,4-O-Bu_3_ManNAc), which intercept and increase flux through the sialic acid biosynthetic pathway with the indicated relative efficiencies (“R.E.” values) increasing sialylation of recombinant glycoproteins, such as mAbs. **(B)** Alternatively, cells can be supplemented with analogs containing non-natural chemical moieties (e.g., Ac_4_ManNAz or 1,3,4-O-Bu_3_ManNAz to install azide groups or Ac_4_6-Thio-Fuc to install thiols). These functional groups, which do not naturally occur in glycans, constitute chemical handles for conjugation to small molecules including drugs, toxins, or imaging agents.

ADCs are an emerging class of therapeutics that leverage the specificity of mAbs to minimize off-target effects of small molecule drugs (197, 198). Historically, conjugation of drugs to antibodies typically utilized amino acids such as lysine and cysteine. However, with ~30 surface-exposed lysines and 8 hinge cysteines this strategy yields a heterogenous ADC mixture with a wide distribution of drug antibody ratios resulting in suboptimal pharmacokinetic properties, lower efficacy, and reduced specificity (197, 199, 200). An alternative approach to attach a drug to an antibody is to exploit the glycans located at Asn-279 in the IgG domain as a “chemical handle”—for example, mild oxidation of the terminal sialic acid creates an aldehyde capable of drug conjugation via oxime or hydrazone ligation (201, 202). One pitfall in this approach is that IgG Fc-region glycans are poorly sialylated (<10%) (9) but efforts are underway to increase sialylation or incorporate non-natural sialic acid groups through metabolic glycoengineering (203) (Figure 8A). Alternative strategies include utilizing IgG antibodies with fragment antigen-binding (Fab) glycosylation or targeting fucose instead of sialic acid, a strategy that has been demonstrated with 6-thiofucose (204). Once the glycan moieties of an antibody have been chemically remodeled, a variety of chemoenzymatic ligation methods are available to attach a drug including copper catalyzed or strain-promoted alkyne:azide “click” reactions (197, 205–207) (Figure 8B). Interestingly, ADCs can evoke multiple facets of activity, for example drug-conjugated gPD-L1 antibody (which is the PDL1 blocking antibody mentioned in section Blocking Antibodies) induces a potent cell-killing effect as well as a bystander-killing effect on adjacent cancer cells lacking PD-L1 expression (184, 208).

#### Single domain antibodies and nanobodies

Canonical antibodies are complex, glycosylated molecules comprised of Fab domains linked to a constant Fc region via a flexible hinge region; furthermore, many antibodies are linked to proteins, toxins, small molecule drugs, or radionuclides that increases their size and complexity (209–211). These properties can lead to incorrect domain association and aggregation (6, 212). To circumvent these pitfalls, efforts have been made to engineer mAbs with smaller sizes and fewer domains. This idea was galvanized in the 1990s by the discovery that *Camelidae* (camels) produce fully functional antibodies devoid of light chains (213). This breakthrough has escalated the development of monovalent (Fab, single chain variable fragment (scFv), single variable V_H_ and V_L_ domains) and bivalent (Fab'_2_, dibodies, minibodies) antibody-derived fragments now generally termed single domain antibodies or nanobodies. Single domain antibodies and nanobodies are advantageous due to their small size, high solubility, thermal stability, versatility, refolding capacities, reduced aggregation, high tissue penetration, lack of requirement for PTMs, and ability to be produced in nonmammalian cells (212, 214–216). These properties make single domain antibodies and nanobodies especially attractive for imaging, blocking, and neutralization applications (212, 215).

Although the non-essentiality of PTMs has been a “selling point” for single domain antibodies and nanobodies, glycosites can nevertheless ameliorate and expand the utility of this class of antibodies. For example, PEG conjugated to the N-glycan of scFv increased serum half-life ~10-fold (217). Another study showed that fusing a single domain antibody with N-linked glycosylation to one lacking glycans improved the construct's ability to neutralize foot-and-mouth disease virus 4-fold (218). Interestingly, shark and camel single domain antibodies can naturally contain sites of glycosylation; although the functional importance these glycans is currently unknown (215). These studies suggest that glycosylation can be used to augment the efficacy of single domain antibodies and nanobodies at least in part through physicochemical considerations (section Design Considerations and Biomanufacturing).

### Additional immunomodulatory glycoproteins

In addition to antibodies, the largest category of today's immunotherapeutics (219), many other glycoproteins modulate immunity. Three of these (interferons, interleukins, and colony-stimulating factor) that have already achieved clinical translation are summarized below.

#### Interferons

Interferons are a subclass of cytokines naturally produced by the body. These signaling proteins are grouped into three subclasses (α, β, and γ) according to their cell of origin and inducing agent. Upon binding to their cognate receptors, interferons activate signaling networks that provide antiviral, immunomodulatory, and antiproliferative activity (220). Given their ability to regulate the immune system, these cytokines have been exploited for therapeutic purposes. For example, interferon β–a naturally glycosylated protein—slows the progression of multiple sclerosis, a chronic autoimmune disease resulting in demyelination of nerve sheaths of the central nervous system (221–223). The hyperglycosylation of interferon β enhances its biophysical and pharmacokinetics properties by improving its physicochemical properties (224, 225). Although non-glycosylated interferon β is available, superior versions of glycosylated recombinant interferon β now in clinical use include Avonex® and Rebif® (226, 227).

#### Interleukin-2

Interleukin-2 (IL-2) is a naturally-occurring cytokine and an early example of an immunotherapeutic protein. Recombinant IL-2 is Food and Drug Administration (FDA) approved for treatment of metastatic renal cell carcinoma and metastatic melanoma with clinical trials underway for several additional diseases (228–231). The importance of glycosylation, usually a critical factor in the efficacy of a biologics drug, remains ambiguous for IL-2; the World Health Organization initially established glycosylated IL-2 as the standard for human use. Subsequent screening of glycosylated and non-glycosylated IL-2, however, showed similar bioactivity (232, 233) although glycosylated IL-2 produced in Jurkat cells had superior thermal stability. Nevertheless, T-cell-derived recombinant IL-2 is no longer in use as a therapeutic (234). Instead, today's FDA-approved recombinant IL-2 (e.g., Proleukin [also known as Aldesleukin] and other variants) is produced using *E. coli*, a species that lacks protein glycosylation (235). All in all, IL-2 provides an interesting example of a biologics drug where the role of glycosylation remains ambiguous although, based on overwhelming evidence from other products, we would not be surprised if superior forms of glycosylated IL-2 are developed in the future.

#### Colony stimulating factor

Colony stimulating factors (CSFs) are potent activators of the innate immune system that modulate the activity and populations of granulocytes and macrophages (236), which are critical hematopoietic cells involved in fighting bacterial, viral, and fungal infections. Given this function, CSFs have been explored to activate the immune system; in particular granulocyte-CSF is commonly used to stimulate the bone marrow to increase neutrophil production to treat neutropenia (237). Presently, five types of granulocyte-CSF have been produced using various expression systems including aglycosylated variants in *E. coli* (molgramostim and filgrastim), an O-glycosylated type in yeast (sargramostim), and versions with mammalian-type glycosylation in CHO cells (regramostim and lenograstim) (238, 239). A comparison of these various forms of granulocyte-CSF suggests that glycosylation prolongs serum half-life without significantly affecting biological activity (240).

### Vaccines

As mentioned earlier, vaccines pioneered the field of immunotherapy two centuries ago (241) and remain highly relevant today, as cancer vaccines provide another example (as introduced for MUC1 in section O-Glycans in Immunotherapy and outlined in Figure 5). In the modern era, glycans have become an integral part of vaccine development with polysaccharide-directed vaccines such as PCV13 and PPSV23 constituting a critical defense against pneumococcal infections (242, 243) illustrating how glycoconjugates have emerged as some of the safest and most efficacious vaccines (244). Today, vaccine development almost always requires cognizance of glycosylation with firmly established roles ranging from well established, intensely studied viruses such as HIV (245, 246) and influenza (247, 248) to sporadic and emerging threats such as the ebola (249) and zika viruses.

### Cell-based immunotherapy

Cell-based immunotherapy is rapidly emerging strategy that utilizes living cells such as T-cells, dendritic cells, and mesenchymal stem cells (MSCs) to harness the body's natural immune system to fight disease. In this section we review how glycosylation impacts the efficacy and development of two pioneering cell-based immunotherapies based on CAR T-cells and MSCs.

#### Chimeric antigen receptor (CAR) T-cell therapy

In 1989 Eshhar and coworkers developed a novel CAR that combined a scFv with a transmembrane domain and an intracellular signaling unit, CD3 ζ chain, enabling targeting to specific epitopes and concurrent activation of T-cells without dependence on the major histocompatibility complex molecules (250–252). Subsequent efforts enhanced CAR specificity, reduced off target effects, integrated costimulatory receptors, and increased T-cell proliferation capacity (252, 253). Current CAR T-cell preparation involves six steps: (i) harvesting white blood cells from the patient through leukapheresis, (ii) activating the cells using antibody coated beads, (iii) reprogramming the T-cells utilizing retroviruses to express CARs, (iv) expanding the CAR T-cells *ex vivo*, (v) placing the patient in an immunocompromised state via lymphodepleting chemotherapy, and (vi) transfusing the patient with the engineered CAR T-cells (254, 255).

CAR T-cells have been engineered to target glycan epitopes of glycolipids and glycoproteins aberrantly expressed in cancer including TAG72 (the sialyl Tn O-glycan epitope), the Lewis y antigen (Le^y^), the disialoganglioside GD2, and Tn MUC1 (256, 257). An early CAR T-cell therapy targeting TAG72 failed to elicit a clinical response possibly due to the CARs murine origin, lack of T-cell co-stimulation, or the affinity of the CC49 anti-sialyl Tn mAb (256, 258). A subsequent CAR T-cell therapy against Le^y^ was more successful (259) showing therapeutic potential in a phase I clinical trial (260). The ganglioside GD2, which is commonly overexpressed in neural crest-derived tumors, has been targeted in separate CAR T-cell studies. The first was safe and induced tumor necrosis *in vivo* and provided complete response in three out of eleven patients (261, 262). A subsequent GD2-targeting test conducted in conjunction with lymphodepletion resulted in improved CAR T-cell expansion in patients but failed to significantly improve patient antitumor response and survival time (263). Finally, the Tn and sialyl Tn MUC1 epitopes have been targeted by CAR T-cells using a humanized version of the 5E5 antibody (264). Although glycan-targeting CAR T-cell therapy has yet to achieve FDA approval, prospects are bright with 10 active phase I and II CAR T-cell trials targeting MUC1 glycoforms alone (127, 256).

#### Mesenchymal stem cell (MSC) homing

MSCs, which display potent immunosuppressive properties including inhibiting proliferation and activity of T-cells, inhibiting production of pro-inflammatory cytokines, mediating differentiation of B cells, and inducing macrophages *in vitro* (265, 266), are an emerging type of immunotherapy. Delivery of MSCs *in vivo*, however, typically suffers from inefficient homing and migration of MSCs to the target tissue (267). This pitfall has spurred research in several laboratories to improve MSC homing with efforts largely converging on exploiting selectin-mediated cell trafficking to direct systemically-delivered MSCs to sites of inflammation (or other desired locations, such as the bone marrow) in the body (267).

Selectin-mediated cell trafficking critically depends on the fucose-containing tetrasaccharide sLe^x^ [Neu5Ac-α(2,3)-Gal-β(1,4)-[Fuc-α(1,3)]-GlcNAc-R, Figure 3]. MSCs lack expression of the fucosyltransferases (Fut3-7) required for sLe^X^ synthesis (268, 269); without sLe^x^ MSCs have poor homing ability, which limits their immunotherapeutic potential. This pitfall is being overcome through a variety of strategies where MSCs are endowed with the requisite fucosyltransferase activities needed to create sLe^x^ motif and efficiently home to specific locations in the body (270–274). For example, glycoengineering via glycosyltransferse-programmed stereosubstitution and transfection with modified mRNA has been used to drive over expression of sLe^x^ to augment the homing capacity of numerous cell types including hematopoietic and progenitor stem cells (HSPCs) (275), MSCs (270), neural stem cells (276), and lymphocytes (118, 277).

## Design considerations and biomanufacturing

We begin this section by discussing how the physicochemical properties of glycans—which have been alluded to several times already, mostly in the context of pharmacokinetics—impact immunotherapeutics in section Physicochemical Properties. We then discuss, in section Cell-based Production Options, how the selection of the appropriate host cell as a biomanufacturing platform is crucial for endowing an immunotherapeutic drug with appropriate glycoforms to optimize not only physicochemical properties but also to maintain safety and improve bioactivity. Finally, in section Glycoengineering Approaches to Improve Immunotherapeutics we provide an overview of “glycoengineering” strategies—that typically complement and are fully compatible with cell-based production platforms that are being developed to enhance future immunotherapeutics.

### Physicochemical properties

Physicochemical considerations are critically important during the optimization of virtually all biologics, including immunotherapeutics. Even when the biological properties of a potential drug are tuned for optimal efficacy during early discovery phases, intractable “developability” issues often crop up later related to the physicochemical nature of the candidate. Physicochemical problems that can thwart drug development include difficulties in formulating a biologic for appropriate dosing, absorption to surfaces that causes large variance in delivery, protein aggregation or stability during storage, and solubility. Commonly employed strategies to improve physicochemical properties, such as PEGylation (which is mentioned above, e.g., in section O-Glycans in Immunotherapy), can affect immunity in sometimes unpredictable ways and also adversely impact safety (278–280). The *Guidance for Industry: Immunogenicity Assessment for Therapeutic Protein Products* published by the FDA states that

“For proteins that are normally glycosylated, use of a cell substrate production system and appropriate manufacturing methods that glycosylate the therapeutic protein product in a non-immunogenic manner is recommended (281).”

Consequently, although initially easier to implement than undertaking efforts to optimize pharmacokinetic properties through glycosylation, PEGylation of glycoproteins may require more work in the end because anti-drug antibody assays need to be developed to detect both the anti-protein antibody as well as antibodies against PEGylated epitopes found on the protein. A somewhat underappreciated role for glycosylation is the profound impact that it can have on the physicochemical properties of proteins, many of which are important for the developability of a lead biologic drug candidate. The fact that glycosylation can be viewed as “more natural” by the body (considering human types of glycosylation only), is another advantage that promotes the need for optimized glycoengineering strategies described in this review. Finally, as noted above, glycosylation often tunes biological activity (e.g., Fc effector function) in ways not accessible through PEGylation. Below, we discuss the impact that glycosylation has on the physicochemical properties of therapeutic proteins and the development of biologic drug candidates.

#### Protein aggregation

Many amino acids are electrically charged, are basic or acidic, or contain a thiol; the peptide backbone of a biologic is therefore typically vulnerable to unwanted and difficult-to-control chemical reactivity, and problems such as protein aggregation are often encountered during development. Aggregate bodies can elicit immunogenicity that ultimately leads to the intolerance and rejection of drug candidates (282). It has long been observed, however, that glycosylation can significantly improve the aggregation properties of proteins. For example, O-linked glycosylation can suppress the polymerization of an immunomodulating protein like human granulocyte-CSF (283). Crystallographic analysis of glycosylated interferon β marketed by Pfizer (Rebif®) revealed this drug was 10 times more potent than its unglycosylated counterpart due to the prevention of the formation of large, soluble aggregates (224, 284). In fact, interferon β produced in *E. coli* that is unglycosylated quantitatively contains about 60% aggregates that elicit antibodies in a high portion of patients while the glycosylated form contains only ~2% aggregates and is far less immunogenic (285).

From a production perspective, prevention of aggregate formation is important for improving yields of useable drug product (286). Aglycosylation—a strategy typically employed to simplify the production of antibodies—can increase aggregation (287). The prevention of protein aggregation by glycosylation is a complex physicochemical phenomenon that is not easily rationalized simply by the attachment of a hydrophilic constituent to a protein because glycans theoretically interact less favorably with water than the peptide backbone (286). Nevertheless, in theory, glycosylation slows aggregation by increasing the molecular solvent accessible surface area of a protein. In one study, increased glycosylation changed the surface area of the glycoconjugates from ~9,000 Å to ~16,000 Å, and the exposed surface area of the protein concomitantly decreased (from ~9,000 Å to ~5,000 Å), which influenced the internal electrostatic and biophysical properties of proteins through a steric diaelectric effect (288). Glycoengineering and optimization of production platform glycosylation stands to improve both the biomanufacturing process and biological drug properties of immunotherapeutics.

#### Colloidal stability

Another important physicochemical parameter that influences aggregation is colloidal stability. Proteins have intrinsic colloidal properties and most, if not all, biologics are administered and stored as solutions; therefore, improving the colloidal stability of protein therapeutics is critically important for shelf-life. Høberg-Nielsen and co-workers, for example, demonstrated that glycosylation promoted colloidal stability of aggregation-prone forms of the phytase enzyme from *Pheniophoria lycii* (286). In addition to the influence that N-glycans have on Fc receptor binding, these glycans stabilize the Fc C_H_2 regions of mAbs by protecting against aggregation through colloidal properties (289). Interestingly, previous studies have shown that under conditions of high temperature and high concentration (60°C and 20 mg/mL) aggregation in the model protein α-chymotrypsin could not be inhibited by a small glycan, but two or more larger glycans improved colloidal stability and abrogated aggregation (290). Based on this precedent, and others, the glycoengineering of immunotherapeutics is expected to improve shelf-life and ameliorate formulation issues by modulating of the colloidal properties of these proteins.

#### Conformational stability

Over the last 30 years nuclear magnetic resonance (NMR), circular dichroism, Förster resonance energy transfer (FRET), and powerful *in silico* techniques have provided important insights into how glycosylation influences the secondary structure and conformational dynamics of a protein (291). Complementary NMR-FRET studies have shown that β-turns followed by a surface loop transition, a common motif for sites of N-linked glycosylation, have a more compact peptide secondary structure when glycosylated with a chitobiosyl disaccharide group. These regions adopt an open and extended Asn-turn conformation when aglycosylated while the introduction of a glycan results in a compact type I β-turn structure, illustrating how glycosylation can serve as a “conformational switch” for proteins (291, 292). These observations also correlated with the *in silico* statistical calculations performed by Petrescu et al. who surveyed 506 glycoproteins and found that N-glycans alter the distribution of torsion angles within the protein to possibly reduce overall flexibility (293). Similarly, earlier elegant work revealed that oligosaccharides enhanced global dynamic stability and the unfolding equilibrium of RNaseB, and furthermore, this effect could be observed as far as 30 Å away from the site of glycosylation (294). The take home message is that glycosylation can serve to alter the equilibrium states between folded and unfolded proteins and can help select for small populations of conformers that have defined, stable, and precise structure (e.g., proteins with N-glycan proximal to their β-loops). Ultimately, this increased glycan-mediated stability complements glycan-mediated benefits related to aggregation and the colloidal properties of glycoproteins as discussed above.

#### Protection of proteins from oxidation

Another physicochemical feature of biologics tuned by glycosylation is susceptibility to oxidative insult. Because extracellular space is an oxidizing environment, the half-life, distribution, and efficacy of immunotherapeutics could be enhanced by resistance to oxidative stresses ubiquitous inside of a living organism. Again, glycosylation is beneficial because it can protect the polypeptide backbones of proteins from free-radical damage (295); protection was linked to the total degree of glycosylation and not any specific glycan or sugar moiety, indicating that “highly branched” glycans would be broadly protective. In the model protein EPO, oxidative damage to tryptophan that led to loss of biological activity, was thwarted by glycosylation (296). Related to immunotherapy, oxidation of methionine and tryptophan triggers the degradation of monoclonal antibodies (297, 298) and interferons are also susceptible to oxidation (299–301). In general, oxidized proteins also are immunogenic, an unwanted attribute of immunotherapeutic drugs; interestingly, despite earlier examples where glycans were the source of immunogenicity (e.g., for α-Gal or Neu5Gc, Figure 3) the examples provided in this paragraph illustrate how glycans can instead be protective by minimizing oxidative damage.

#### Physicochemical conclusions

Although the impact of glycosylation on immunotherapeutics is often focused on biological function, glycans also have a powerful ability to tailor physicochemical features critical for clinical translation and commercial developability. Specifically, glycosylation can optimize physicochemical considerations of biologics to improve features such as shelf-life, colloidal stability, resistance to oxidation, and the avoidance of unwanted immunogenicity. Although synthetic techniques such as PEGylation have been extensively used to improve physicochemical properties, control of glycosylation—achieved through appropriate selection of cell line for production (section Cell-based Production Options) or through glycoengineering methods (section Glycoengineering Approaches to Improve Immunotherapeutics)—can potentially provide superior results because glycosylation has been developed by nature over hundreds of millions of years to finely regulate the biology of proteins.

### Cell-based production options

Early generations of immunotherapeutics, such as vaccines, largely were produced in embryonated eggs or collected from animal products and human blood donations (5, 302). Today's immunotherapeutics, however, exploit recombinant DNA technology to produce proteins in cell-based manufacturing platforms (whereas certain immunotherapies, as discussed above [section Cell-based Immunotherapy], consist of the cells themselves). Cell-based biomanufacturing efforts have explored a wide range of expression systems including non-mammalian (bacteria, yeast, plant, and insect) and mammalian (human, hamster, and mouse) cells (179) to optimize product yield and install appropriate PTMs. From 2004 to 2013 biopharmaceuticals approved by the FDA and European Medicines Agency (EMA) were predominantly obtained from mammalian cells (56%), *E. scoli* (24%), *Saccharomyces cerevisiae* (13%), insect cells (4%), and transgenic animals and plants (3%) (303). The majority of products, obtained from mammalian cells, includes virtually all recent therapeutic proteins (including immunotherapeutics) where PTMs, especially glycosylation, can be optimized for safety, biological activity, function, stability, physicochemical properties, and pharmacokinetics (2, 111, 304). For this reason—after providing a brief synopsis of non-mammalian options (section Non-mammalian Cell lines)—we focus on the selection of mammalian expression systems used in biomanufacturing beginning with the use of human (section Human Cell Lines) and murine (section Murine Cell Lines) cell lines used in the early production of modern immunotherapeutics (i.e., mAbs). As discussed below, each of these cell lines had substantial pitfalls, leading to today's consolidation of production in CHO cells (section Chinese Hamster Ovary (CHO) Cells).

#### Non-mammalian cell lines

Insulin, the earliest recombinant human protein, was produced in *E. coli*, which benefits from low cost and high productivity (303, 305). Although a few biologics are still produced in *E. coli* (e.g., IL-2, as described in section Interleukin-2), the lack of N-glycans that ensure quality control during folding (306) makes prokaryotic production untenable for most glycoproteins including mAbs. Yeast (*S. cerevisiae* and *Pichia pastoris*) provide another high productivity, low cost production platform (307, 308) and—being eukaryotic cells—do have N-glycans; yeast glycans, however, tend to be highly mannosylated which reduces serum longevity thus compromising pharmacokinetics and also impacting downstream effector functions (309). Even though efforts have been made to “humanize” yeast glycosylation, these cells have not become a widely-accepted biomanufacturing platform (309). Finally, insect (e.g., *Trichoplusia and Drosophila*) cells have been investigated for recombinant glycoprotein production, but despite efforts to humanize glycosylation (310–312), these cells also have substantial pitfalls for biomanufacturing including minimal sialylation ability (311, 313).

#### Human cell lines

The inability of the initial bacterial, yeast, and insect production platforms to produce properly glycosylated human proteins led to production efforts in human cells. The first immortalized human cell line, HeLa, was derived from cervical cancer in 1951 (314) and paved the way for the development of other immortalized human cell lines, notably human embryonic kidney 293 (HEK293) and fibrosarcoma HT-1080 cells used to produce viral vaccines (106, 180, 315). However, it wasn't until ~2001 that the first therapeutic glycoprotein produced in human cells (HEK293), Drotecogin alfa, was approved by the FDA and EMA; since then several glycoprotein immunotherapeutics have been produced in human cells primarily in the HEK293 and HT-1080 lines (179).

Human cells offer important advantages over other production platforms including the ability to closely mimic PTMs, particularly glycosylation, naturally found in people. For example, human cells lines express Mgat3, α(1,3/4)-fucosyl transferase, and α(2,6)-sialyltransferse which are silent or missing in CHO cells. Furthermore, human cell lines do not produce immunogenic structures, such as α-Gal and N-glycolylneuraminic acid (Neu5Gc), thus minimizing safety and compatibility concerns. These factors reduce the need to genetically engineer cells and limit the cost of downstream processing (106, 180, 316). Although human cells have these attractive features as production platform, they also have substantial limitations and drawbacks. For example, human lines suffer from low growth rates, production capacities, and protein yields making them impractical for the production of many therapeutic proteins including mAbs. Furthermore, the absence of a species barrier makes human cell lines a significant safety risk due to the potential for contamination and transmission of human pathogens. In theory, these disadvantages can be overcome with advances in technology and adherence to stringent good manufacturing practices (106, 180, 316); in practice, most immunotherapeutics are now produced in rodent cells, as described next.

#### Murine cell lines

Murine myeloma cells, predominantly NS0 and Sp2/0, are another cell platform that is periodically used for the production of recombinant glycoproteins. Both the NS0 and Sp2/0 cell lines were developed from tumors and subsequently genetically engineered to stop producing their native immunoglobins yet retain the cellular machinery to secrete recombinant proteins at high levels (317, 318). Accordingly these lines have been used to produce of the commercial mAbs Cetuximab, Palivizumab, Dinutuximab, Necitumumab, and Elotuzumab (179, 180, 319). A downside of murine cells is their ability to incorporate α-Gal and Neu5Gc into glycans, thereby presenting a considerable risk of immunogenicity (49, 320, 321). Thus, murine cells used for therapeutic protein production must be thoroughly screened for clones lacking these immunogenic epitopes while producing desirable glycan profiles.

#### Chinese hamster ovary (CHO) cells

In 1986 tissue plasminogen became the first FDA-approved recombinant biopharmaceutical to be produced in CHO cells (180, 316, 322); since then these cells have become the predominant manufacturing platform for biologics producing an estimated 70% of recombinant biopharmaceutical proteins (2, 323, 324). Furthermore, over 90% of commercial antibodies are now produced in CHO cells (6, 179, 180). The success of CHO cells in commercial biomanufacturing stems from several key advantages. First, CHO cells can be grown in large bioreactors as a cell suspension in serum-free, chemically-defined media while maintaining high production rates. From a safety perspective, many viral entry genes are not expressed in CHO cells and there is a species barrier that minimizes risk of transferring infectious agents to humans (325, 326). Furthermore, over the past three decades the extensive documentation that CHO cells are safe hosts aids in facilitating regulatory approval to bring immunotherapeutics to the market (316, 322). Perhaps most importantly, CHO cells produce recombinant glycoproteins with compatible glycoforms that are bioactive in humans (179, 180, 322, 327).

Despite the advantages of CHO cell production platforms, shortcomings exist. CHO cells (as with most mammalian cell lines) retain the ability to produce glycans not found in humans including α-Gal and Neu5Gc (320, 328). Humans inherently express antibodies against these immunogenic epitopes that can lead to severe, potentially fatal immunogenic responses and/or negate the effects of immunotherapeutics (49, 320, 321). However, the levels of α-Gal and Neu5Gc are relatively low (<2% Neu5Gc and <0.2% α-Gal) in CHO cells, meaning this issue can be circumvented by selecting clones lacking these non-human epitopes (179, 320). CHO cells also lack certain types of glycosylation found in humans, such as α(2,6)-sialylation, α(1,3/4) -fucosylation, and bisecting GlcNAc (329–332). Overcoming these differences by “humanizing” CHO cell glycosylation is, at least in theory, possible through genetic and metabolic “glycoengineering” approaches, as discussed next in section Glycoengineering Approaches to Improve Immunotherapeutics.

### Glycoengineering approaches to improve immunotherapeutics

Various approaches to modulate glycans in living cells—i.e., “glycoengineering” methods—have developed over the past ~3 decades during the same time as the importance of glycosylation in immunity has been unraveled. Today, these parallel developments have set the stage to employ the various glycoengineering strategies now available to generate recombinant proteins (or even entire cells) with desirable glycan profiles (12, 333, 334) during immunotherapeutic design and manufacturing. Glycoengineering falls into two main approaches: genetic and metabolic; we will discuss specific examples of both approaches while describing general strengths and drawbacks to each approach. Although glycoengineering strategies are being developed for many production platforms [bacteria (161), yeast (335), plants (336), insects (337)], we will focus our discussion on mammalian cells used to produce the vast majority of today's immunotherapeutics.

#### Genetic approaches to glycoengineering

Many genetic approaches have been used to target glycosylation pathways and enzymes via gene knockdown, knockout, overexpression, knockin, and selective nucleotide mutation. These “genetic engineering” strategies have been used to reduce or silence undesirable glycosyltransferase activities, enhance glycosyltransferase activities, activate endogenously silent genes, introduce new glycosites, mimic hypomorphic disease mutations, and insert foreign genes (334). In recent years, genetic glycoengineering has been galvanized by the discovery and development of zinc-finger nucleases, transcription activator-like effector nucleases (TALENs), and clustered regularly interspaced short palindromic repeats/targeted Cas endonuclease (CRISPR/Cas) technology (334, 338, 339). A strength of genetic approaches is their versatility and ability to make permanent cellular modifications; however, genetic approaches have limitations such as off-target effects, inefficient *in vivo* delivery systems, confounding epigenetic regulation of glycosylation pathways, and unpredictable alterations to cellular physiology (334, 340).

Sialic acid is one of the most frequently targeted monosaccharides for glycoengineering due to its manifold impact on the pharmacokinetics of recombinant glycoproteins in general and its specific impact on bioactivity in ADCC, IVIG, and ADCs. Genetic manipulation of sialyltransferases constitutes a common approach to glycoengineer sialic acid; in particular β-galactoside α(2,6)-sialyltransferases (usually ST6GAL1) in CHO cells enables the production of glycoproteins with both α(2,3)-sialic acids (from the cells' endogenous STs) and α(2,6)-linked sialic acids (from the newly-expressed ST6GAL1), similar to glycoproteins produced in humans (339, 341–343). In addition, overexpression of ST6GAL1 (or other sialyltransferases) increases the overall sialylation of therapeutic glycoproteins including EPO (343–345), tissue plasminogen activator (342, 346), interferon γ (347, 348), and IgG (346, 349, 350). Other studies have targeted the preceding step, the addition of galactose, to enhance terminal sialylation levels. Multiple studies have demonstrated that concomitant over-expression of β(1,4)-galactosyltranferase and α(2,3)-sialyltranferase in CHO cells yielded increased sialylation and galactosylation in EPO, IgG, and tissue plasminogen activiator (344, 346). Another strategy is to overexpress Mgat4 and 5 to increase tri- and tetra-antennary branched N-glycans, thereby creating more sites for terminal sialylation; this strategy has been employed in EPO (345), albumin EPO (351), and interferon γ (352, 353).

Another strategy for improving sialylation targets enzymes and transporters in the sialic acid biosynthetic pathway to increase CMP-Neu5Ac levels. One approach recapitulated point mutations in the bifunctional enzyme UDP-GlcNAc 2-epimerase/ManNAc kinase (GNE) associated with sialuria (354, 355), a congenital disease that leads to excessive synthesis of sialic acid due to the absence of feedback regulation (356), which led to increases in intracellular CMP-sialic acid levels and EPO sialylation (357, 358). Although increasing intracellular CMP-Neu5Ac levels can increase glycoprotein sialylation there may be a saturation point due to the inefficiency of the CMP-sialic acid transporter responsible for transporting CMP-Neu5Ac to the Golgi. To overcome this barrier one study overexpressed CMP-sialic acid transporter in CHO cells, but only saw modest increases (4–16%) in interferon γ sialylation (359). Inhibiting or eliminating sialidases (or neuraminidases) is a complementary strategy for enhancing glycoprotein sialylation; these enzymes are glycosidases that catalyze the hydrolytic removal of sialic acid from glycoproteins, glycolipids, and polysaccharides (360). One study utilized short interfering RNA and short-hairpin RNA to lower expression of the *Neu1* and *Neu3* sialidase in CHO cells, which increased recombinant interferon γ sialylation by up to 33% (361).

In another approach, genetic glycoengineering can be utilized to introduce new glycosites into glycoproteins through creation of the Asn-X-Ser/Thr consensus sequence for N-glycosylation. This approach is illustrated by darbepoetin alfa, a genetically modified form of EPO that has five (instead of three) N-glycan sites (362); this enhanced level of glycosylation improved serum longevity ~3-fold (362) but was accompanied by adverse effects such as increased risk of stroke (363). (As a caveat, there is no evidence from carefully controlled studies that increased risk is a general feature of over-glycosylated therapeutic proteins beyond darbepoeitin alfa or a direct consequence of the newly-installed glycans). Another interesting example of “building in” N-glycosites is provided by Ibalizumab, where the strategic addition of an N-glycan to this mAb improves its HIV-neutralizing activity (364). In the future, installation of glycans on various immunotherapeutics, e.g., *Camelidae* antibodies (section Single Domain Antibodies and Nanobodies), may prove enhance the physicochemical properties and translational potential of these emerging drugs.

#### Metabolic glycoengineering

The second major strategy to control glycosylation is metabolic glycoengineering (MGE), where living cells or entire organisms are supplemented with monosaccharide precursors that either increase natural flux through a biosynthetic pathway (Figure 8A) or increasingly, substitute natural metabolites with their non-natural counterparts (Figure 8B). The exogenously-supplied synthetic monosaccharides are processed by the biosynthetic pathway, ultimately yielding glycans with enhanced glycoforms (e.g., improved sialylation) or non-natural chemical groups (203, 365, 366). Historically the sialic acid biosynthetic pathway has been the premier target of MGE due to this pathway's tolerance for non-natural variants of mannosamine or sialic acid (203). One advantage of MGE is its simplicity, where an analog can be directly added cell culture medium to exploit the intrinsic cellular machinery without any need to genetically manipulate the host cell, thus averting off-target complications. However, MGE can be non-trivial because of the need for custom synthesis of the required monosaccharides and expensive to implement on an industrial scale because their concentration in culture media must be maintained to obtain a desired glycan profile (203, 365).

One application of MGE relevant to immunotherapy involves increasing therapeutic glycoprotein sialylation through supplementation with ManNAc (367), this strategy, outlined in Figure 8A in the context of IgG antibodies has the potential to increase the physicochemical and pharmacokinetic properties of these antibodies, endow them with anti-inflammatory activity needed for IVIg therapy, or provide sialic acids required for ADC production [Neu5Ac can be oxidized to contain an aldehyde group allowing for drug conjugation via oxime ligation (201)]. A pitfall for ManNAc supplementation is that millimolar concentrations of ManNAc are required, which increase intracellular CMP-sialic acid levels up to 12-fold but only produces moderate gains in protein sialylation (368–370); the requirement for large concentrations of ManNAc (e.g., 20–50 mM) to achieve modest improvements are impractical from a biomanufacturing perspective due to the cost of ManNAc ($20 / g or higher). Efforts have long been underway to improve the efficiency of monosaccharide analogs intended as metabolic supplements ranging from fluorinated ManNAc analogs in the early 1980s (371) to disaccharides in the mid-1990s (372) to non-natural ManNAc analogs used in MGE in the late 1990s (372) through peracetylation (e.g., as illustrated by Ac_4_ManNAc, Figure 8A). Despite improving efficiency by ~900-fold (373), growth inhibition and cytotoxicity (374, 375) limit the application of per-acetylated analogs in a biomanufacturing setting. To circumvent these issues our group has developed (376, 377) and characterized (378) butyrated ManNAc analogs that can be applied to culture medium in micromolar concentrations. The analog's butyrate groups enhance cellular uptake by ~2,100-fold and are subsequently cleaved by nonspecific esterases allowing the ManNAc to intercept and increase flux through the sialic acid biosynthetic pathway (379). Supplementation of CHO cells with the “high-flux” ManNAc analog (1,3,4-O-Bu_3_ManNAc, Figure 8A) improves EPO and IgG sialylation (Figure 8) (380, 381) and in theory, could be used to augment the pharmacokinetic and physicochemical properties of any recombinant immunotherapeutic.

In a second MGE-based approach, ManNAc analogs can be used to install non-natural chemical moieties into glycans (Figure 8B), in essence creating a chemical handle for bioorthogonal conjugation of small molecules including toxins, drugs, genes, imaging agents, and polymers (203). This strategy has been used to incorporate numerous non-natural functional groups such as ketones (382–384), azides (377, 385), alkynes (386), diazirines (387), aryl azides (388), and thiols (389) into glycans for subsequent conjugation via click chemistry. A sialic acid-based MGE approach can be used to introduce conjugation sites restricted to the Fc region of mAbs for developing ADCs (373, 390, 391); similarly, the fucose-replacing analog 6-thiofucose can introduce thiol moieties into 70% of IgG heavy chains with 90% conjugation efficiency to small molecule drugs via maleimide chemistry (204). As superior metabolic analogs [e.g., butyrated ManNAz [1,3,4-O-Bu_3_ManNAz], Figure 8B (377)] and conjugation chemistries [e.g., strain-promoted alkyne:azide cycloaddition (376, 377)] are developed we anticipate a bright future for MGE-based ADCs.

#### Combined genetic and metabolic engineering approaches

The field of MGE has often been regarded as a genetically “silent” method to label glycans based on the assumption that the “glycosylation machinery” of a cell is not substantially perturbed while processing the exogenously-supplied sugars required for this methodology. While this premise is basically accurate, our group (and others) have described how metabolic flux engendered by MGE monosaccharide analogs (and even natural sugars) can on occasion affect the expression of “glycogenes” with this effect most well studied for the sialic acid biosynthetic pathway (392–395). The ability of MGE analogs to affect gene expression and cell physiology extends beyond glycogenes *per se* and can have a profound impact on cellular processes such as cell differentiation (396–398). We briefly mention these effects both to caution researchers to the complex interplay between metabolic, genetics, and cell fate that can occur during MGE interventions but also to highlight the opportunities to use this technology to tune biological activity, which we fully anticipate will facilitate future generations of immunotherapy.

## Conclusion and future perspectives

Over the past 30 years immunotherapy has become the most promising approach for developing new medicines and treating disease. In order to maintain the rapid advancement of immunotherapies it is critical to not only optimize glycosylation for maximal efficacy but also exploit these macromolecules to ameliorate existing treatments. To reach these goals it is vital to better understand the underlying biology of glycosylation which requires the ongoing development of novel tools for studying glycosylation and continued improvement of carbohydrate chemistry methods. Moving forward areas of glycobiology not typical associated with immunotherapy, such as O-linked glycosylation [both mucin-type and other forms, such as the intracellular “O-GlcNAc” PTM now being linked to immunity (399–402)] and glycolipids, are sure to offer new opportunities for creating biotherapeutics. Finally, although immunotherapy has already achieved substantial success in treating disease we are only scratching the surface, therefore we foresee glycosylation a key to helping immunotherapy realize its full potential in the future.

## Author contributions

KJY and MJB: Writing, editing, figures; CTS: Writing, editing; SRS, RA: Writing.

### Conflict of interest statement

The authors declare that the research was conducted in the absence of any commercial or financial relationships that could be construed as a potential conflict of interest.

## Abbreviations

ADC: antibody-drug conjugates
ADCC: antibody-dependent cell cytotoxicity (also referred to as antibody-dependent cellular cytotoxicity or antibody-dependent cell-mediated cytotoxicity)
ASGP: asialoglycoprotein
CAR: chimeric antigen receptor
CDC: complement dependent cytotoxicity
CHO: Chinese hamster ovary
CRISPR/Cas: clustered regularly interspaced short palindromic repeats/targeted Cas endonuclease
CSF: colony stimulating factor
EMA: European Medicines Agency
EPO: erythropoietin
ER: endoplasmic reticulum
Fab: fragment antigen-binding
Fcγ: (fragment crystallizable γ)
FDA: Food and Drug Administration
FRET: Förster resonance energy transfer
Fuc: fucose
Fut/FUT: fucosyltransferase
Gal: galactose
GalCer: galactosylceramide
GalNAc: N-acetylgalactosamine
Glc: glucose
GlcCer: glucosylceramide
GlcNAc: N-acetylglucosamine
GSL: glycosphingolipid
HEK293: human embryonic kidney 293 cells
HSPC: hematopoietic stem and progenitor cell
IgG: immunoglobin G
IL-2: interleukin-2
IVIG: intravenous immunoglobin
LacNAc: Galβ(1-4)GlcNAc
LLO: lipid-linked oligosaccharide (Glc_3_Man_9_GlcNAc_2_-P-P-dolichol)
mAb: monoclonal antibody
Man: mannose
Mgat: N-acetylglucosaminyltransferase
MSC: mesenchymal stem (or stromal) cell
MUC1: mucin 1
Neu5Ac: N-acetylneuraminic acid
Neu5Gc: N-glycolylneuraminic acid
NMR: nuclear magnetic resonance
PD1: programmed cell death protein-1
PDL1: programmed death ligand-1
PEG: polyethylene glycol
PTM: post-translational modifications
scFv: single chain variable fragment
SIGN-R1 or DC-SIGN: specific intracellular adhesion molecule-grabbing nonintegrin R1
sLe^x^: sialyl Lewis x
TALEN: transcription activator-like effector nuclease
α-Gal: galactose-α(1,3)-galactose.

## References

[1] Huang CJ Lowe AJ Batt CA. Recombinant immunotherapeutics: current state and perspectives regarding the feasibility and market. Appl Microbiol Biotechnol. (2010) 87:401–10. 10.1007/s00253-010-2590-7 20422181

[2] Ghaderi D Zhang M Hurtado-Ziola N Varki A. Production platforms for biotherapeutic glycoproteins. Occurrence, impact, and challenges of non-human sialylation. Biotechnol Genet Eng Rev. (2012) 28:147–75. 10.5661/bger-28-147 22616486

[3] Berg P Mertz JE. Personal reflections on the origins and emergence of recombinant DNA technology. Genetics (2010) 184:9–17. 10.1534/genetics.109.112144 20061565 PMC2815933

[4] Tibaldi JM. Evolution of insulin development: focus on key parameters. Adv Ther. (2012) 29:590–619. 10.1007/s12325-012-0034-8 22843207

[5] Fitzhugh DJ Lockey RF. History of immunotherapy: the first 100 years. Immunol Allergy Clin North Am. (2011) 31:57. 10.1016/j.iac.2011.03.003 21530811

[6] Walsh G. Biopharmaceutical benchmarks 2014. Nat Biotechnol. (2014) 32:992–1000. 10.1038/nbt.3040 25299917

[7] Anonymous Global Immunotherapy Market Research Report 2018. The Market Reports (2018). p. 1–98.

[8] Chhina M Overview of biological products. Center for Drug Evaluation and Research U S Food and Drug Administration (2013).

[9] Arnold JN Wormald MR Sim RB Rudd PM Dwek RA. The impact of glycosylation on the biological function and structure of human immunoglobulins. Annu Rev Immunol. (2007) 25:21–50. 10.1146/annurev.immunol.25.022106.141702 17029568

[10] Dicker M Strasser R. Using glyco-engineering to produce therapeutic proteins. Exp Opin Biol Ther. (2015) 15:1501–16. 10.1517/14712598.2015.1069271 26175280 PMC7100909

[11] Jefferis R. Glycosylation as a strategy to improve antibody-based therapeutics. Nat Rev Drug Discov. (2009) 8:226–34. 10.1038/nrd2804 19247305

[12] Sola RJ Griebenow K. Glycosylation of therapeutic proteins: an effective strategy to optimize efficacy. BioDrugs (2010) 24:9–21. 10.2165/11530550-000000000-00000 20055529 PMC2805475

[13] Freire T Osinaga E. The sweet side of tumor immunotherapy. Immunotherapy (2012) 4:719–34. 10.2217/imt.12.58 22853758

[14] Daniotti JL Vilcaes AA Torres Demichelis V Ruggiero FM Rodriguez-Walker M. Glycosylation of glycolipids in cancer: basis for development of novel therapeutic approaches. Front Oncol. (2013) 3:306. 10.3389/fonc.2013.00306 24392350 PMC3867695

[15] Reusch D Tejada ML. Fc glycans of therapeutic antibodies as critical quality attributes. Glycobiology (2015) 25:1325–34. 10.1093/glycob/cwv065 26263923 PMC4634315

[16] Varki A Kornfeld S Chapter 1: Historical background and overview. In: Varki A Cummings R Esko J Stanley P Hart G Aebi M Darvill A Kinoshita T Packer N Prestegard J Schaar R Seeberger P editors. Essentials of Glycobiology. Cold Spring Harbor, NY: Cold Spring Harbor Laboratory Press (2017).

[17] Dwek RA. Glycobiology: toward understanding the function of sugars. Chem Rev. (1996) 96:683–720. 10.1021/cr940283b 11848770

[18] Washburn N Schwab I Ortiz D Bhatnagar N Lansing JC Medeiros A. Controlled tetra-Fc sialylation of IVIg results in a drug candidate with consistent enhanced anti-inflammatory activity. Proc Natl Acad Sci USA. (2015) 112:1297. 10.1073/pnas.1422481112 25733881 PMC4371931

[19] Stanley P Taniguchi N Aebi M Chapter 8: N-Glycans. In: Varki A Cummings R Esko J Stanley P Hart G Aebi M Darvill A Kinoshita T Packer N Prestegard J Schnaar R Seeberger P editors. Essentials of Glycobiology. Cold Spring Harbor, NY: Cold Spring Harbor Laboratory Press (2017).

[20] Bieberich E. Synthesis, processing, and function of N-glycans in N-glycoproteins. Adv Neurobiol. (2014) 9:47–70. 10.1007/978-1-4939-1154-7_3 25151374 PMC4236024

[21] Chen H Wang Z Sun Z Kim EK Yarema KJ. Chapter 1. Mammalian glycosylation: an overview of carbohydrate biosynthesis. In: Yarema KJ, editor. Handbook of Carbohydrate Engineering. Boca Raton, FL: CRC Press (Taylor & Francis Group) (2005). p. 1–48.

[22] Meledeo MA Yarema KJ Glycan biosynthesis and glycosylation in mammals. In: Begley T, editor. Wiley Encyclopedia of Chemical Biology. (Hoboken, NJ) (2008). p. 1–16. 10.1002/9780470048672.wecb190

[23] Aebi M. N-linked protein glycosylation in the ER. Biochim Biophys Acta (2013) 1833, 2430–2437. 10.1016/j.bbamcr.2013.04.001 23583305

[24] Kornfeld R Kornfeld S. Assembly of asparagine-linked oligosaccharides. Annu Rev Biochem. (1985) 54:631–64. 10.1146/annurev.bi.54.070185.003215 3896128

[25] Chojnacki T Dallner G. The biological role of dolichol. Biochem J. (1988) 251:1–9. 10.1042/bj2510001 3291859 PMC1148956

[26] Rush JS Waechter CJ. Transmembrane movement of a water-soluble analogue of mannosylphosphoryldolichol is mediated by an endoplasmic reticulum protein. J Cell Biol. (1995) 130:529–36. 10.1083/jcb.130.3.529 7622555 PMC2120537

[27] Sanyal S Menon AK. Stereoselective transbilayer translocation of mannosyl phosphoryl dolichol by an endoplasmic reticulum flippase. Proc Natl Acad Sci USA. (2010) 107:11289–94. 10.1073/pnas.1002408107 20534553 PMC2895134

[28] Mohorko E Glockshuber R Aebi M. Oligosaccharyltransferase: the central enzyme of N-linked protein glycosylation. J Inherit Metab Dis. (2011) 34:869–78. 10.1007/s10545-011-9337-1 21614585

[29] Kelleher DJ Gilmore R. An evolving view of the eukaryotic oligosaccharyltransferase. Glycobiology (2006) 16:62R. 10.1093/glycob/cwj066 16317064

[30] Cheng K Zhou Y Neelamegham S. DrawGlycan-SNFG: a robust tool to render glycans and glycopeptides with fragmentation information. Glycobiology (2017) 27:200–5. 10.1093/glycob/cww115 28177454 PMC6410959

[31] Krambeck FJ Bennun SV Narang S Choi S Yarema KJ Betenbaugh MJ. A mathematical model to derive N-glycan structures and cellular enzyme activities from mass spectrometric data. Glycobiology (2009) 19:1163–75. 10.1093/glycob/cwp081 19506293 PMC2757573

[32] Krambeck FJ Betenbaugh MJ. A mathematical model of N-linked glycosylation. Biotechnol Bioeng. (2005) 92:711–28. 10.1002/bit.20645 16247773

[33] Werz DB Ranzinger R Herget S Adibekian A von der Lieth CW Seeberger PH. Exploring the structural diversity of mammalian carbohydrates (“glycospace”) by statistical databank analysis. ACS Chem Biol. (2007) 2:685–91. 10.1021/cb700178s 18041818

[34] Driouich A Gonnet P Makkie M Laine AC Faye L. The role of high-mannose and complex asparagine-linked glycans in the secretion and stability of glycoproteins. Planta (1989) 180:96–104. 10.1007/BF02411415 24201849

[35] Goetze AM Liu YD Zhang Z Shah B Lee E Bondarenko PV. High-mannose glycans on the Fc region of therapeutic IgG antibodies increase serum clearance in humans. Glycobiology (2011) 21:949–59. 10.1093/glycob/cwr027 21421994

[36] Yu M Brown D Reed C Chung S Lutman J Stefanich E. Production, characterization, and pharmacokinetic properties of antibodies with N-linked mannose-5 glycans. MAbs (2012) 4:475–87. 10.4161/mabs.20737 22699308 PMC3499342

[37] Shi HH Goudar CT. Recent advances in the understanding of biological implications and modulation methodologies of monoclonal antibody N-linked high mannose glycans. Biotechnol Bioeng (2014) 111:1907–19. 10.1002/bit.25318 24975601

[38] Chen HL Li CF Grigorian A Tian W Demetriou M. T cell receptor signaling co-regulates multiple Golgi genes to enhance N-glycan branching. J Biol Chem (2009) 284:32454–61. 10.1074/jbc.M109.023630 19706602 PMC2781660

[39] Kanda Y Yamane-Ohnuki N Sakai N Yamano K Nakano R Inoue M. Comparison of cell lines for stable production of fucose-negative antibodies with enhanced ADCC. Biotechnol Bioeng. (2006) 94:680–8. 10.1002/bit.20880 16609957

[40] Kanda Y Yamada T Mori K Okazaki A Inoue M Kitajima-Miyama K. Comparison of biological activity among nonfucosylated therapeutic IgG1 antibodies with three different N-linked Fc oligosaccharides: the high-mannose, hybrid, and complex types. Glycobiology (2007) 17:104–18. 10.1093/glycob/cwl057 17012310

[41] Liu L. Antibody glycosylation and its impact on the pharmacokinetics and pharmacodynamics of monoclonal antibodies and Fc-fusion proteins. J Pharm Sci. (2015) 104:1866–84. 10.1002/jps.24444 25872915

[42] Hiatt A Bohorova N Bohorov O Goodman C Kim D Pauly MH. Glycan variants of a respiratory syncytial virus antibody with enhanced effector function and in vivo efficacy. Proc Natl Acad Sci USA. (2014) 111:5992–7. 10.1073/pnas.1402458111 24711420 PMC4000855

[43] Schachter H. Biosynthetic controls that determine the branching and microheterogeneity of protein-bound oligosaccharides. Biochem Cell Biol. (1986) 64:163–81. 10.1139/o86-026 3521675

[44] Rose DR. Structure, mechanism and inhibition of Golgi α-mannosidase II. Curr Opin Struct Biol. (2012) 22:558–62. 10.1016/j.sbi.2012.06.005 22819743

[45] Varki A Gagneux P. Multifarious roles of sialic acids in immunity. Ann N Y Acad Sci. (2012) 1253:16–36. 10.1111/j.1749-6632.2012.06517.x 22524423 PMC3357316

[46] Büll C den Brok MH Adema GJ. Sweet escape: sialic acids in tumor immune evasion. Biochim Biophys Acta Rev Cancer (2014) 1846:238–46. 10.1016/j.bbcan.2014.07.005 25026312

[47] Shitara K Enhancement of ADCC of Antibodies by Glycoengineering. Available online at: http://www.glycoforum.gr.jp/science/word/immunity/IS-A06E.html (Accessed August 7, 2018).

[48] Samraj AN Pearce OM Laubli H Crittenden AN Bergfeld AK Banda K. A red meat-derived glycan promotes inflammation and cancer progression. Proc Natl Acad Sci USA. (2015) 112:542–47. 10.1073/pnas.1417508112 25548184 PMC4299224

[49] Chung CH Mirakhur B Chan E Le QT Berlin J Morse M. Cetuximab-induced anaphylaxis and IgE specific for galactose-α-1,3-galactose. N Engl J Med. (2008) 358:1109–17. 10.1056/NEJMoa074943 18337601 PMC2361129

[50] Dennis JW Nabi IR Demetriou M. Metabolism, cell surface organization, and disease. Cell (2009) 139:1229–41. 10.1016/j.cell.2009.12.008 20064370 PMC3065826

[51] Demetriou M Granovsky M Quaggin S Dennis JW. Negative regulation of T-cell activation and autoimmunity by Mgat5 N-glycosylation. Nature (2001) 409:733–9. 10.1038/35055582 11217864

[52] Morgan R Gao G Pawling J Dennis JW Demetriou M Li B. N-acetylglucosaminyltransferase V (Mgat5)-mediated N-glycosylation negatively regulates Th1 cytokine production by T cells. J Immunol. (2004) 173:7200–8. 10.4049/jimmunol.173.12.7200 15585841

[53] Zhao Y Sato Y Isaji T Fukuda T Matsumoto A Miyoshi E. Branched N-glycans regulate the biological functions of integrins and cadherins. FEBS J. (2008) 275:1939–48. 10.1111/j.1742-4658.2008.06346.x 18384383

[54] Lau KS Partridge EA Grigorian A Silvescu CI Reinhold VN Demetriou M. Complex N-glycan number and degree of branching cooperate to regulate cell proliferation and differentiation. Cell (2007) 129:123–34. 10.1016/j.cell.2007.01.049 17418791

[55] Bumbaca D Boswell CA Fielder PJ Khawli LA. Physiochemical and biochemical factors influencing the pharmacokinetics of antibody therapeutics. AAPS J. (2012) 14:554–8. 10.1208/s12248-012-9369-y 22610647 PMC3385840

[56] Misaizu T Matsuki S Strickland TW Takeuchi M Kobata A Takasaki S. Role of antennary structure of N-linked sugar chains in renal handling of recombinant human erythropoietin. Blood (1995) 86:4097–104. 7492766

[57] Weiss P Ashwell G. The asialoglycoprotein receptor: properties and modulation by ligand. Prog Clin Biol Res. (1989) 300:169–84. 2674962

[58] Kizuka Y Taniguchi N. Enzymes for N-glycan branching and their genetic and nongenetic regulation in cancer. Biomolecules (2016) 6:E25. 10.3390/biom6020025 27136596 PMC4919920

[59] Gu J Nishikawa A Tsuruoka N Ohno M Yamaguchi N Kangawa K. Purification and characterization of UDP-N-acetylglucosamine: α-6-D-mannoside β-1–6N-acetylglucosaminyltransferase (N-acetylglucosaminyltransferase V) from a human lung cancer cell line. J Biochem. (1993) 113:614–9. 10.1093/oxfordjournals.jbchem.a124091 8393437

[60] Pinho SS Reis CA Paredes J Magalhaes AM Ferreira AC Figueiredo J. The role of N-acetylglucosaminyltransferase III and V in the post-transcriptional modifications of E-cadherin. Hum Mol Genet. (2009) 18:2599–608. 10.1093/hmg/ddp194 19403558

[61] Song Y Aglipay JA Bernstein JD Goswami S Stanley P. The bisecting GlcNAc on N-glycans inhibits growth factor signaling and retards mammary tumor progression. Cancer Res. (2010) 70:3361–71. 10.1158/0008-5472.CAN-09-2719 20395209 PMC2856092

[62] Yoshimura M Nishikawa A Ihara Y Taniguchi S Taniguchi N. Suppression of lung metastasis of B16 mouse melanoma by N-acetylglucosaminyltransferase III gene transfection. Proc Natl Acad Sci USA. (1995) 92:8754–8. 10.1073/pnas.92.19.8754 7568011 PMC41045

[63] Yoshimura M Ihara Y Matsuzawa Y Taniguchi N. Aberrant glycosylation of E-cadherin enhances cell-cell binding to suppress metastasis. J Biol Chem. (1996) 271:13811–5. 10.1074/jbc.271.23.13811 8662832

[64] Ihara Y Yoshimura M Miyoshi E Nishikawa A Sultan AS Toyosawa S Ohnishi A. Ectopic expression of N-acetylglucosaminyltransferase III in transgenic hepatocytes disrupts apolipoprotein B secretion and induces aberrant cellular morphology with lipid storage. Proc Natl Acad Sci USA. (1998) 95:2526–30. 10.1073/pnas.95.5.2526 9482919 PMC19400

[65] Xu Q Isaji T Lu Y Gu W Kondo M Fukuda T. Roles of N-acetylglucosaminyltransferase III in epithelial-to-mesenchymal transition induced by transforming growth factor β1 (TGF-β1) in epithelial cell lines. J Biol Chem. (2012) 287:16563–74. 10.1074/jbc.M111.262154 22451656 PMC3351319

[66] Pinho SS Oliveira P Cabral J Carvalho S Huntsman D Gartner F. Loss and recovery of Mgat3 and GnT-III mediated E-cadherin N-glycosylation is a mechanism involved in epithelial-mesenchymal-epithelial transitions. PLoS ONE (2012) 7:e33191. 10.1371/journal.pone.0033191 22427986 PMC3302839

[67] Lu J Isaji T Im S Fukuda T Kameyama A Gu J. Expression of N-Acetylglucosaminyltransferase III suppresses α2,3-sialylation, and its distinctive functions in cell migration are attributed to α2,6-sialylation levels. J Biol Chem. (2016) 291:5708–20. 10.1074/jbc.M115.712836 26801611 PMC4786709

[68] Davies J Jiang L Pan LZ LaBarre MJ Anderson D Reff M Expression of GnTIII in a recombinant anti-CD20 CHO production cell line: expression of antibodies with altered glycoforms leads to an increase in ADCC through higher affinity for FC g RIII. Biotechnol Bioeng. (2001) 74:288–94. 10.1002/bit.1119 11410853

[69] Li H Sethuraman N Stadheim TA Zha D Prinz B Ballew N Bobrowicz P. Optimization of humanized IgGs in glycoengineered *Pichia pastoris*. Nat Biotechnol. (2006) 24:210–5. 10.1038/nbt1178 16429149

[70] Shields RL Lai J Keck R O'Connell LY Hong K Meng YG Lack of fucose on human IgG1 N-linked oligosaccharide improves binding to human Fcg RIII and antibody-dependent cellular toxicity. J Biol Chem. (2002) 277:26733–40. 10.1074/jbc.M202069200 11986321

[71] Iida S Misaka H Inoue M Shibata M Nakano R Yamane-Ohnuki N Nonfucosylated therapeutic IgG1 antibody can evade the inhibitory effect of serum immunoglobulin G on antibody-dependent cellular cytotoxicity through its high binding to FcgRIIIa. Clin Cancer Res. (2006) 12:2879–87. 10.1158/1078-0432.CCR-05-2619 16675584

[72] Koyota S Ikeda Y Miyagawa S Ihara H Koma M Honke K. Down-regulation of the α-Gal epitope expression in N-glycans of swine endothelial cells by transfection with the N-acetylglucosaminyltransferase III gene. Modulation of the biosynthesis of terminal structures by a bisecting GlcNAc. J Biol Chem. (2001) 276:32867–74. 10.1074/jbc.M102371200 11443114

[73] Qasba PK Ramakrishnan B Boeggeman E. Structure and function of β-1,4-galactosyltransferase. Curr Drug Targets (2008) 9:292–309. 10.2174/138945008783954943 18393823 PMC2365515

[74] Furukawa K Sato T. β-1,4-galactosylation of N-glycans is a complex process. Biochim Biophys Acta (1999) 1473, 54–66. 10.1016/S0304-4165(99)00169-5 10580129

[75] Millward TA Heitzmann M Bill K Langle U Schumacher P Forrer K. Effect of constant and variable domain glycosylation on pharmacokinetics of therapeutic antibodies in mice. Biologicals (2008) 36:41–7. 10.1016/j.biologicals.2007.05.003 17890101

[76] Scallon BJ Tam SH McCarthy SG Cai AN Raju TS. Higher levels of sialylated Fc glycans in immunoglobulin G molecules can adversely impact functionality. Mol Immunol. (2007) 44:1524–34. 10.1016/j.molimm.2006.09.005 17045339

[77] Raju TS Jordan RE. Galactosylation variations in marketed therapeutic antibodies. MAbs (2012) 4:385–91. 10.4161/mabs.19868 22531450 PMC3355481

[78] Raju TS. Terminal sugars of Fc glycans influence antibody effector functions of IgGs. Curr Opin Immunol. (2008) 20:471–8. 10.1016/j.coi.2008.06.007 18606225

[79] Boyd PN Lines AC Patel AK. The effect of the removal of sialic acid, galactose and total carbohydrate on the functional activity of Campath-1H. Mol Immunol. (1995) 32:1311–8. 10.1016/0161-5890(95)00118-2 8643100

[80] Reichert JM. Probabilities of success for antibody therapeutics. MAbs (2009) 1:387–9. 10.4161/mabs.1.4.9031 20068390 PMC2726601

[81] Strohl WR Knight DM. Discovery and development of biopharmaceuticals: current issues. Curr Opin Biotechnol. (2009) 20:668–72. 10.1016/j.copbio.2009.10.012 19896824

[82] O'Neil BH Allen R Spigel DR Stinchcombe TE Moore DT Berlin JD. High incidence of cetuximab-related infusion reactions in Tennessee and North Carolina and the association with atopic history. J Clin Oncol. (2007) 25:3644–48. 10.1200/JCO.2007.11.7812 17704414

[83] Steinke JW Platts-Mills TA Commins SP. The α-gal story: lessons learned from connecting the dots. J Allergy Clin Immunol. (2015) 135:96; quiz 597. 10.1016/j.jaci.2014.12.1947 25747720 PMC4600073

[84] Prudden AR Liu L Capicciotti CJ Wolfert MA Wang S Gao Z. Synthesis of asymmetrical multiantennary human milk oligosaccharides. Proc Natl Acad Sci USA. (2017) 114:6954–9. 10.1073/pnas.1701785114 28630345 PMC5502611

[85] Javaud C Dupuy F Maftah A Julien R Petit JM. The fucosyltransferase gene family: an amazing summary of the underlying mechanisms of gene evolution. Genetica (2003) 118:157–70. 10.1023/A:1024101625214 12868606

[86] Stanley P Cummings R Chapter 14: Structures common to different glycans. In: Varki A Cummings R Esko J Stanley P Hart G Aebi M Darvill A Kinoshita T Packer N Prestegard J Schnaar R Seeberger P, editors. Essentials of Glycobiology. Cold Spring Harbor, NY: Cold Spring Harbor Laboratory Press (2017).

[87] Wang X Inoue S Gu J Miyoshi E Noda K Li W Dysregulation of TGF-b1 receptor activation leads to abnormal lung development and emphysema-like phenotype in core fucose-deficient mice. Proc Natl Acad Sci USA. (2005) 102:15791–6. 10.1073/pnas.0507375102 16236725 PMC1257418

[88] Wang X Gu J Ihara H Miyoshi E Honke K Taniguchi N. Core fucosylation regulates epidermal growth factor receptor-mediated intracellular signaling. J Biol Chem. (2006) 281:2572–7. 10.1074/jbc.M510893200 16316986

[89] Li W Nakagawa T Koyama N Wang X Jin J Mizuno-Horikawa Y. Down-regulation of trypsinogen expression is associated with growth retardation in α1,6-fucosyltransferase-deficient mice: attenuation of proteinase-activated receptor 2 activity. Glycobiology (2006) 16:1007–19. 10.1093/glycob/cwl023 16861703

[90] Ito Y Miyauchi A Yoshida H Uruno T Nakano K Takamura Y. Expression of α1,6-fucosyltransferase (FUT8) in papillary carcinoma of the thyroid: its linkage to biological aggressiveness and anaplastic transformation. Cancer Lett. (2003) 200:167–72. 10.1016/S0304-3835(03)00383-5 14568171

[91] Noda K Miyoshi E Uozumi N Gao CX Suzuki K Hayashi N. High expression of α-1–6 fucosyltransferase during rat hepatocarcinogenesis. Int J Cancer (1998) 75:444–50. 10.1002/(SICI)1097-0215(19980130)75<444::AID-IJC19>3.0.CO;2-8 9455807

[92] Honma R Kinoshita I Miyoshi E Tomaru U Matsuno Y Shimizu Y. Expression of fucosyltransferase 8 is associated with an unfavorable clinical outcome in non-small cell lung cancers. Oncology (2015) 88:298–308. 10.1159/000369495 25572677

[93] Potapenko IO Haakensen VD Luders T Helland A Bukholm I Sorlie T. Glycan gene expression signatures in normal and malignant breast tissue; possible role in diagnosis and progression. Mol Oncol. (2010) 4:98–118. 10.1016/j.molonc.2009.12.001 20060370 PMC5527897

[94] Wang X Chen J Li QK Peskoe SB Zhang B Choi C. Overexpression of α (1,6) fucosyltransferase associated with aggressive prostate cancer. Glycobiology (2014) 24:935–44. 10.1093/glycob/cwu051 24906821 PMC4153758

[95] Rothman RJ Perussia B Herlyn D Warren L. Antibody-dependent cytotoxicity mediated by natural killer cells is enhanced by castanospermine-induced alterations of IgG glycosylation. Mol Immunol. (1989) 26:1113–23. 10.1016/0161-5890(89)90055-2 2633046

[96] Satoh M Iida S Shitara K. Non-fucosylated therapeutic antibodies as next-generation therapeutic antibodies. Expert Opin Biol Ther. (2006) 6:1161–73. 10.1517/14712598.6.11.1161 17049014

[97] Okazaki A Shoji-Hosaka E Nakamura K Wakitani M Uchida K Kakita S. Fucose depletion from human IgG1 oligosaccharide enhances binding enthalpy and association rate between IgG1 and FcγRIIIa. J Mol Biol. (2004) 336:1239–49. 10.1016/j.jmb.2004.01.007 15037082

[98] Natsume A Wakitani M Yamane-Ohnuki N Shoji-Hosaka E Niwa R Uchida K. Fucose removal from complex-type oligosaccharide enhances the antibody-dependent cellular cytotoxicity of single-gene-encoded bispecific antibody comprising of two single-chain antibodies linked to the antibody constant region. J Biochem. (2006) 140:359–68. 10.1093/jb/mvj157 16861252

[99] Mori K Kuni-Kamochi R Yamane-Ohnuki N Wakitani M Yamano K Imai H. Engineering Chinese hamster ovary cells to maximize effector function of produced antibodies using FUT8 siRNA. Biotechnol Bioeng. (2004) 88:901–8. 10.1002/bit.20326 15515168

[100] Ito A Ishida T Yano H Inagaki A Suzuki S Sato F. Defucosylated anti-CCR4 monoclonal antibody exercises potent ADCC-mediated antitumor effect in the novel tumor-bearing humanized NOD/Shi-scid, IL-2Rγ(null) mouse model. Cancer Immunol Immunother. (2009) 58:1195–206. 10.1007/s00262-008-0632-0 19048251 PMC11030985

[101] Li T DiLillo DJ Bournazos S Giddens JP Ravetch JV Wang LX. Modulating IgG effector function by Fc glycan engineering. Proc Natl Acad Sci USA. (2017) 114:3485–90. 10.1073/pnas.1702173114 28289219 PMC5380036

[102] Nimmerjahn F Ravetch JV. Antibodies, Fc receptors and cancer. Curr Opin Immunol. (2007) 19:239–45. 10.1016/j.coi.2007.01.005 17291742

[103] Sakae Y Satoh T Yagi H Yanaka S Yamaguchi T Isoda Y. Conformational effects of N-glycan core fucosylation of immunoglobulin G Fc region on its interaction with Fcγ receptor IIIa. Sci Rep. (2017) 7:8. 10.1038/s41598-017-13845-8 29062024 PMC5653758

[104] Yamane-Ohnuki N Kinoshita S Inoue-Urakubo M Kusunoki M Iida S Nakano R. Establishment of FUT8 knockout Chinese hamster ovary cells: an ideal host cell line for producing completely defucosylated antibodies with enhanced antibody-dependent cellular cytotoxicity. Biotechnol Bioeng. (2004) 87:614–22. 10.1002/bit.20151 15352059

[105] Luo C Chen S Xu N Wang C Sai WB Zhao W. Glycoengineering of pertuzumab and its impact on the pharmacokinetic/pharmacodynamic properties. Sci Rep. (2017) 7:46347. 10.1038/srep46347 28397880 PMC5387714

[106] Goh JB Ng SK. Impact of host cell line choice on glycan profile. Crit Rev Biotechnol. (2018) 38:851–67. 10.1080/07388551.2017.1416577 29262720

[107] Castilho A Gruber C Thader A Oostenbrink C Pechlaner M Steinkellner H. Processing of complex N-glycans in IgG Fc-region is affected by core fucosylation. MAbs (2015) 7:863–70. 10.1080/19420862.2015.1053683 26067753 PMC4622071

[108] Angata T Varki A. Chemical diversity in the sialic acids and related α-keto acids: an evolutionary perspective. Chem Rev. (2002) 102:439–69. 10.1021/cr000407m 11841250

[109] Harduin-Lepers A Vallejo-Ruiz V Krzewinski-Recchi MA Samyn-Petit B Julien S Delannoy P. The human sialyltransferase family. Biochimie (2001) 83:727–37. 10.1016/S0300-9084(01)01301-3 11530204

[110] Li Y Chen X. Sialic acid metabolism and sialyltransferases: natural functions and applications. Appl Microbiol Biotechnol. (2012) 94:887–905. 10.1007/s00253-012-4040-1 22526796 PMC3534974

[111] Bork K Horstkorte R Weidemann W. Increasing the sialylation of therapeutic glycoproteins: the potential of the sialic acid biosynthetic pathway. J Pharm Sci. (2009) 98:3499–508. 10.1002/jps.21684 19199295

[112] Morell AG Gregoriadis G Scheinberg IH Hickman J Ashwell G. The role of sialic acid in determining the survival of glycoproteins in the circulation. J Biol Chem. (1971) 246:1461–7. 5545089

[113] Tang L Persky AM Hochhaus G Meibohm B. Pharmacokinetic aspects of biotechnology products. J Pharm Sci. (2004) 93:2184–204. 10.1002/jps.20125 15295780

[114] Weinstein T Gafter U Chagnac A Skutelsky E. Distribution of glycosaminoglycans in rat renal tubular epithelium. J Am Soc Nephrol. (1997) 8:586–95. 10495788 10.1681/ASN.V84586

[115] Samuelsson A Towers TL Ravetch JV. Anti-inflammatory activity of IVIG mediated through the inhibitory Fc receptor. Science (2001) 291:484–6. 10.1126/science.291.5503.484 11161202

[116] Sondermann P Pincetic A Maamary J Lammens K Ravetch JV. General mechanism for modulating immunoglobulin effector function. Proc Natl Acad Sci USA. (2013) 110:9868–72. 10.1073/pnas.1307864110 23697368 PMC3683708

[117] Anthony RM Wermeling F Karlsson MC Ravetch JV. Identification of a receptor required for the anti-inflammatory activity of IVIG. Proc Natl Acad Sci USA. (2008) 105:19571–8. 10.1073/pnas.0810163105 19036920 PMC2604916

[118] Sackstein R Schatton T Barthel SR. T-lymphocyte homing: an underappreciated yet critical hurdle for successful cancer immunotherapy. Lab Invest. (2017) 97:669. 10.1038/labinvest.2017.25 28346400 PMC5446300

[119] Brockhausen I Stanley P Chapter 10: O-GalNAc Glycans. In: Varki A Cummings R Esko J Stanley P Hart G Aebi M Darvill A Kinoshita T Packer N Prestegard J Schnaar R Seeberger P editors. Essentials of Glycobiology. Cold Spring Harbors, NY: Cold Spring Harbor Laboratory Press (2017).

[120] Haltiwanger R Wells L Freeze H Stanley P Other classess of eukaryotic glycans. In: Varki A Cummings R Esko J Stanley P Hart G Aebi M Darvill A Kinoshita T Packer N Prestegard J Schnaar R Seeberger P, editors. Essentials of Glycobiology. Cold Spring Harbor, NY: Cold Spring Harbor Laboratory Press (2017).

[121] Hanisch FG. O-glycosylation of the mucin type. Biol Chem. (2001) 382:143–9. 10.1515/BC.2001.022 11308013

[122] Van den Steen P Rudd PM Dwek RA Opdenakker G. Concepts and principles of O-linked glycosylation. Crit Rev Biochem Mol Biol. (1998) 33:151–208. 10.1080/10409239891204198 9673446

[123] Bennett EP Mandel U Clausen H Gerken TA Fritz TA Tabak LA. Control of mucin-type O-glycosylation: a classification of the polypeptide GalNAc-transferase gene family. Glycobiology (2012) 22:736–56. 10.1093/glycob/cwr182 22183981 PMC3409716

[124] Ten Hagen KG Fritz TA Tabak LA. All in the family: the UDP-GalNAc:polypeptide N-acetylgalactosaminyltransferases. Glycobiology (2003) 13:16R. 10.1093/glycob/cwg007 12634319

[125] Kong Y Joshi HJ Schjoldager KT Madsen TD Gerken TA Vester-Christensen MB. Probing polypeptide GalNAc-transferase isoform substrate specificities by in vitro analysis. Glycobiology (2015) 25:55–65. 10.1093/glycob/cwu089 25155433 PMC4245906

[126] Schachter H Brockhausen I. The biosynthesis of branched O-glycans. Symp Soc Exp Biol. (1989) 43:1–26. 2701469

[127] Taylor-Papadimitriou J Burchell JM Graham R Beatson R. Latest developments in MUC1 immunotherapy. Biochem Soc Trans. (2018) 46:659–68. 10.1042/BST20170400 29784646 PMC6008591

[128] Kimura T Finn OJ. MUC1 immunotherapy is here to stay. Exp Opin Biol Ther. (2013) 13:35–49. 10.1517/14712598.2012.725719 22998452

[129] Hossain MK Wall KA. Immunological evaluation of recent MUC1 glycopeptide cancer vaccines. Vaccines (2016) 4:E25. 10.3390/vaccines4030025 27472370 PMC5041019

[130] Beatson RE Taylor-Papadimitriou J Burchell JM. MUC1 immunotherapy. Immunotherapy (2010) 2:305–27. 10.2217/imt.10.17 20635898

[131] Lakshminarayanan V Thompson P Wolfert MA Buskas T Bradley JM Pathangey LB. Immune recognition of tumor-associated mucin MUC1 is achieved by a fully synthetic aberrantly glycosylated MUC1 tripartite vaccine. Proc Natl Acad Sci USA. (2012) 109:261–6. 10.1073/pnas.1115166109 22171012 PMC3252914

[132] Pan Y Ayani T Nadas J Wen S Guo Z. Accessibility of N-acyl-D-mannosamines to N-acetyl-D-neuraminic acid aldolase. Carbohydr Res. (2004) 339:2091–100. 10.1016/j.carres.2004.05.028 15280054 PMC3177532

[133] Guo Z Shao N. Glycopeptide and glycoprotein synthesis involving unprotected carbohydrate building blocks. Med Res Rev. (2005) 25:655–78. 10.1002/med.20033 15895471

[134] Pan Y Chefalo P Nagy N Harding C Guo Z. Synthesis and immunological properties of N-modified GM3 antigens as therapeutic cancer vaccines. J Med Chem. (2005) 48:875–83. 10.1021/jm0494422 15689172 PMC3180873

[135] Qiu L Gong X Wang Q Li J Hu H Wu Q. Combining synthetic carbohydrate vaccines with cancer cell glycoengineering for effective cancer immunotherapy. Cancer Immunol Immunother. (2012) 61:2045–54. 10.1007/s00262-012-1224-6 22539085 PMC3874797

[136] Oei AL Moreno M Verheijen RH Sweep FC Thomas CM Massuger LF. Induction of IgG antibodies to MUC1 and survival in patients with epithelial ovarian cancer. Int J Cancer (2008) 123:1848–53. 10.1002/ijc.23725 18661524

[137] Mitchell PL Quinn MA Grant PT Allen DG Jobling TW White SC. A phase 2, single-arm study of an autologous dendritic cell treatment against mucin 1 in patients with advanced epithelial ovarian cancer. J Immunother Cancer (2014) 2:16. eCollection 2014. 10.1186/2051-1426-2-16 24995129 PMC4080759

[138] Lepisto AJ Moser AJ Zeh H Lee K Bartlett D McKolanis JR. A phase I/II study of a MUC1 peptide pulsed autologous dendritic cell vaccine as adjuvant therapy in patients with resected pancreatic and biliary tumors. Cancer Ther. (2008) 6:955–64. 19129927 PMC2614325

[139] Morse MA Niedzwiecki D Marshall JL Garrett C Chang DZ Aklilu M. A randomized phase II study of immunization with dendritic cells modified with poxvectors encoding CEA and MUC1 compared with the same poxvectors plus GM-CSF for resected metastatic colorectal cancer. Ann Surg (2013) 258:879–86. 10.1097/SLA.0b013e318292919e 23657083 PMC3812363

[140] Posey AD Jr Schwab RD Boesteanu AC Steentoft C Mandel U Engels B. Engineered CAR T cells targeting the cancer-associated Tn-glycoform of the membrane mucin MUC1 control adenocarcinoma. Immunity (2016) 44:1444–54. 10.1016/j.immuni.2016.05.014 27332733 PMC5358667

[141] Maher J Wilkie S Davies DM Arif S Picco G Julien S. Targeting of tumor-associated glycoforms of MUC1 with CAR T cells. Immunity (2016) 45:945–6. 10.1016/j.immuni.2016.10.014 27851917

[142] You F Jiang L Zhang B Lu Q Zhou Q Liao X. Phase 1 clinical trial demonstrated that MUC1 positive metastatic seminal vesicle cancer can be effectively eradicated by modified Anti-MUC1 chimeric antigen receptor transduced T cells. Sci China Life Sci. (2016) 59:386–97. 10.1007/s11427-016-5024-7 26961900

[143] Wei X Lai Y Li J Qin L Xu Y Zhao R. PSCA and MUC1 in non-small-cell lung cancer as targets of chimeric antigen receptor T cells. Oncoimmunology (2017) 6:e1284722. 10.1080/2162402X.2017.1284722 28405515 PMC5384358

[144] Novak J Tomana M Kilian M Coward L Kulhavy R Barnes S. Heterogeneity of O-glycosylation in the hinge region of human IgA1. Mol Immunol. (2000) 37:1047–56. 10.1016/S0161-5890(01)00019-0 11399322

[145] Xue J Zhu LP Wei Q. IgG-Fc N-glycosylation at Asn297 and IgA O-glycosylation in the hinge region in health and disease. Glycoconj J. (2013) 30:735–45. 10.1007/s10719-013-9481-y 23783413

[146] Takahashi K Hiki Y Odani H Shimozato S Iwase H Sugiyama S. Structural analyses of O-glycan sugar chains on IgA1 hinge region using SELDI-TOFMS with various lectins. Biochem Biophys Res Commun. (2006) 350:580–7. 10.1016/j.bbrc.2006.09.075 17022936

[147] Takahashi N Tetaert D Debuire B Lin LC Putnam FW. Complete amino acid sequence of the δ heavy chain of human immunoglobulin D. Proc Natl Acad Sci USA. (1982) 79:2850–4. 10.1073/pnas.79.9.2850 6806818 PMC346304

[148] Gala FA Morrison SL. The role of constant region carbohydrate in the assembly and secretion of human IgD and IgA1. J Biol Chem. (2002) 277:29005–11. 10.1074/jbc.M203258200 12023968

[149] Kim H Yamaguchi Y Masuda K Matsunaga C Yamamoto K Irimura T. O-glycosylation in hinge region of mouse immunoglobulin G2b. J Biol Chem. (1994) 269:12345–50. 7512967

[150] Plomp R Dekkers G Rombouts Y Visser R Koeleman CA Kammeijer GS. Hinge-region O-glycosylation of human immunoglobulin G3 (IgG3). Mol Cell Proteom. (2015) 14:1373–84. 10.1074/mcp.M114.047381 25759508 PMC4424406

[151] Arnold JN Radcliffe CM Wormald MR Royle L Harvey DJ Crispin M. The glycosylation of human serum IgD and IgE and the accessibility of identified oligomannose structures for interaction with mannan-binding lectin. J Immunol. (2004) 173:6831–40. 10.4049/jimmunol.173.11.6831 15557177

[152] Stefanich EG Ren S Danilenko DM Lim A Song A Iyer S. Evidence for an asialoglycoprotein receptor on nonparenchymal cells for O-linked glycoproteins. J Pharmacol Exp Ther. (2008) 327:308–15. 10.1124/jpet.108.142232 18728239

[153] Liu L Gomathinayagam S Hamuro L Prueksaritanont T Wang W Stadheim TA. The impact of glycosylation on the pharmacokinetics of a TNFR2:Fc fusion protein expressed in glycoengineered *Pichia pastoris*. Pharm Res. (2013) 30:803–12. 10.1007/s11095-012-0921-3 23135825

[154] DeFrees S Wang ZG Xing R Scott AE Wang J Zopf D. GlycoPEGylation of recombinant therapeutic proteins produced in *Escherichia coli*. Glycobiology (2006) 16:833–43. 10.1093/glycob/cwl004 16717104

[155] Zundorf I Dingermann T. PEGylation–a well-proven strategy for the improvement of recombinant drugs. Pharmazie (2014) 69:323–6. 10.1691/ph.2014.3867 24855821

[156] Hansen JE Lund O Tolstrup N Gooley AA Williams KL Brunak S. NetOglyc: prediction of mucin type O-glycosylation sites based on sequence context and surface accessibility. Glycoconj J. (1998) 15:115–30. 10.1023/A:1006960004440 9557871

[157] Julenius K Molgaard A Gupta R Brunak S. Prediction, conservation analysis, and structural characterization of mammalian mucin-type O-glycosylation sites. Glycobiology (2005) 15:153–64. 10.1093/glycob/cwh151 15385431

[158] Tarp MA Clausen H. Mucin-type O-glycosylation and its potential use in drug and vaccine development. Biochim Biophys Acta (2008) 1780, 546–63. 10.1016/j.bbagen.2007.09.010 17988798

[159] Dombrovskiy VY Martin AA Sunderram J Paz HL. Rapid increase in hospitalization and mortality rates for severe sepsis in the United States: a trend analysis from 1993 to 2003^*^. Crit Care Med. (2007) 35:1244–50. 10.1097/01.CCM.0000261890.41311.E9 17414736

[160] Wilson RP Winter SE Spees AM Winter MG Nishimori JH Sanchez JF. The Vi capsular polysaccharide prevents complement receptor 3-mediated clearance of *Salmonella enterica* serotype Typhi. Infect Immun. (2011) 79:830–7. 10.1128/IAI.00961-10 21098104 PMC3028862

[161] Saeui CT Urias E Liu L Mathew MP Yarema KJ. Metabolic glycoengineering bacteria for therapeutic, recombinant protein, and metabolite production applications. Glycoconj J. (2015) 32:425–41. 10.1007/s10719-015-9583-9 25931032 PMC5753405

[162] Piazza M Rossini C Della Fiorentina S Pozzi C Comelli F Bettoni I. Glycolipids and benzylammonium lipids as novel antisepsis agents: synthesis and biological characterization. J Med Chem. (2009) 52:1209–13. 10.1021/jm801333m 19161283

[163] Mitov IG Terziiski DG. Immunoprophylaxis and immunotherapy of gram-negative sepsis and shock with antibodies to core glycolipids and lipid A of bacterial lipopolysaccharides. Infection (1991) 19:383–90. 10.1007/BF01726444 1816107

[164] Wang X Quinn PJ Yan A. Kdo2 -lipid A: structural diversity and impact on immunopharmacology. Biol Rev Camb Philos Soc. (2015) 90:408–27. 10.1111/brv.12114 24838025 PMC4402001

[165] Schnaar R Kinoshita T Chapter 11: Glycospingolipids. In: Varki A Cummings R Esko J Stanley P Hart G Aebi M Darvill A Kinoshita T Packer N Prestegard J Schnaar R Seeberger P, editors. Essentials of Glycobiology. Cold Spring Harbor, NY: Cold Spring Harbor Laboratory Press (2017).

[166] Hakomori S. Structure, organization, and function of glycosphingolipids in membrane. Curr Opin Hematol. (2003) 10:16–24. 10.1097/00062752-200301000-00004 12483107

[167] D'Angelo G Capasso S Sticco L Russo D. Glycosphingolipids: synthesis and functions. FEBS J. (2013) 280:6338–53. 10.1111/febs.12559 24165035

[168] Zhuo D Li X Guan F. Biological roles of aberrantly expressed glycosphingolipids and related enzymes in human cancer development and progression. Front Physiol. (2018) 9:466. 10.3389/fphys.2018.00466 29773994 PMC5943571

[169] Furman WL Shulkin BL Federico SM McCarville MB Davidoff AM Krasin MJ Early response rates and Curie scores at end of induction: An update from a phase II study of an anti-GD2 monoclonal antibody (mAb) with chemotherapy (CT) in newly diagnosed patients (pts) with high-risk (HR) neuroblastoma (NB). JCO (2017) 35:10534 10.1200/JCO.2017.35.15_suppl.10534

[170] Lee J Kim J Kim S Kang J Lee DH Cho BC. P1.01–070 BIW-(8962) an Anti-GM2 ganglioside monoclonal antibody, in advanced/recurrent lung cancer: A phase I/II study. J Thorac Oncol. (2017) 12:S1922. 10.1016/j.jtho.2017.09.72411909707

[171] Gabri MR Cacciavillano W Chantada GL Alonso DF. Racotumomab for treating lung cancer and pediatric refractory malignancies. Exp Opin Biol Ther. (2016) 16:573–8. 10.1517/14712598.2016.1157579 26903265

[172] Danishefsky SJ Shue YK Chang MN Wong CH. Development of Globo-H cancer vaccine. Acc Chem Res. (2015) 48:643–52. 10.1021/ar5004187 25665650

[173] Gasser O Sharples KJ Barrow C Williams GM Bauer E Wood CE. A phase I vaccination study with dendritic cells loaded with NY-ESO-1 and α-galactosylceramide: induction of polyfunctional T cells in high-risk melanoma patients. Cancer Immunol Immunother. (2018) 67:285–98. 10.1007/s00262-017-2085-9 29094183 PMC11028320

[174] Kwak CY Park SY Lee CG Okino N Ito M Kim JH. Enhancing the sialylation of recombinant EPO produced in CHO cells via the inhibition of glycosphingolipid biosynthesis. Sci Rep. (2017) 7:4. 10.1038/s41598-017-13609-4 29026192 PMC5638827

[175] Wang W Singh S Zeng DL King K Nema S. Antibody structure, instability, and formulation. J Pharm Sci. (2007) 96:1–26. 10.1002/jps.20727 16998873

[176] Aboud-Pirak E Hurwitz E Pirak ME Bellot F Schlessinger J Sela M. Efficacy of antibodies to epidermal growth factor receptor against KB carcinoma in vitro and in nude mice. J Natl Cancer Inst. (1988) 80:1605–11. 10.1093/jnci/80.20.1605 3193478

[177] Karapetis CS Khambata-Ford S Jonker DJ O'Callaghan CJ Tu D Tebbutt NC. K-ras mutations and benefit from cetuximab in advanced colorectal cancer. N Engl J Med. (2008) 359:1757–1765. 10.1056/NEJMoa0804385 18946061

[178] Van Cutsem E Köhne C Hitre E Zaluski J Chang Chien C Makhson A. Cetuximab and chemotherapy as initial treatment for metastatic colorectal cancer. N Engl J Med. (2009) 360:1408–17. 10.1056/NEJMoa0805019 19339720

[179] Lalonde ME Durocher Y. Therapeutic glycoprotein production in mammalian cells. J Biotechnol. (2017) 251:128–40. 10.1016/j.jbiotec.2017.04.028 28465209

[180] Dumont J Euwart D Mei B Estes S Kshirsagar R. Human cell lines for biopharmaceutical manufacturing: history, status, and future perspectives. Crit Rev Biotechnol. (2016) 36:1110–22. 10.3109/07388551.2015.1084266 26383226 PMC5152558

[181] Li CW Lim SO Xia W Lee HH Chan LC Kuo CW. Glycosylation and stabilization of programmed death ligand-1 suppresses T-cell activity. Nat Commun. (2016) 7:12632. 10.1038/ncomms12632 27572267 PMC5013604

[182] Tan S Zhang CW Gao GF. Seeing is believing: anti-PD-1/PD-L1 monoclonal antibodies in action for checkpoint blockade tumor immunotherapy. Signal Transduct Target Ther. (2016) 1:16029. 10.1038/sigtrans.2016.29 29263905 PMC5661648

[183] Balar AV Weber JS. PD-1 and PD-L1 antibodies in cancer: current status and future directions. Cancer Immunol Immunother. (2017) 66:551–64. 10.1007/s00262-017-1954-6 28213726 PMC11028560

[184] Li CW Lim SO Chung EM Kim YS Park AH Yao J. Eradication of triple-negative breast cancer cells by targeting glycosylated PD-L1. Cancer Cell (2018) 33:187–201.e10. 10.1016/j.ccell.2018.01.009 29438695 PMC5824730

[185] Hashimoto G Wright PF Karzon DT. Antibody-dependent cell-mediated cytotoxicity against influenza virus-infected cells. J Infect Dis. (1983) 148:785–94. 10.1093/infdis/148.5.785 6605395

[186] Gómez Román VR Murray JC Weiner LM Chapter 1 - Antibody-Dependent Cellular Cytotoxicity (ADCC). In: Ackerman ME Nimmerjahn F, editors. Antibody Fc. Boston, MA: Academic Press (2014). p. 1–27.

[187] Forthal DN Gach JS Landucci G Jez J Strasser R Kunert R. Fc-glycosylation influences Fcγ receptor binding and cell-mediated anti-HIV activity of monoclonal antibody 2G12. J Immunol. (2010) 185:6876–82. 10.4049/jimmunol.1002600 21041724

[188] Kaneko Y Nimmerjahn F Ravetch JV. Anti-inflammatory activity of immunoglobulin G resulting from Fc sialylation. Science (2006) 313:670–3. 10.1126/science.1129594 16888140

[189] Anthony RM Nimmerjahn F Ashline DJ Reinhold VN Paulson JC Ravetch JV. Recapitulation of IVIG anti-inflammatory activity with a recombinant IgG Fc. Science (2008) 320:373–6. 10.1126/science.1154315 18420934 PMC2409116

[190] Seo Y Ishii Y Ochiai H Fukuda K Akimoto S Hayashida T Cetuximab-mediated ADCC activity is correlated with the cell surface expression level of EGFR but not with the KRAS/BRAF mutational status in colorectal cancer. Oncol Rep. (2014) 31:2115–22. 10.3892/or.2014.3077 24626880

[191] Kimura H Sakai K Arao T Shimoyama T Tamura T Nishio K Antibody-dependent cellular cytotoxicity of cetuximab against tumor cells with wild-type or mutant epidermal growth factor receptor. Cancer Sci. (2007) 98:1275–80. 10.1111/j.1349-7006.2007.00510.x 17498200 PMC11159318

[192] Schwab I Biburger M Kronke G Schett G Nimmerjahn F. IVIg-mediated amelioration of ITP in mice is dependent on sialic acid and SIGNR1. Eur J Immunol. (2012) 42:826–30. 10.1002/eji.201142260 22278120

[193] Anthony RM Ravetch JV. A novel role for the IgG Fc glycan: the anti-inflammatory activity of sialylated IgG Fcs. J Clin Immunol. (2010) 30 (Suppl. 1):S9–14. 10.1007/s10875-010-9405-6 20480216

[194] Schwab I Nimmerjahn F. Intravenous immunoglobulin therapy: how does IgG modulate the immune system? Nat Rev Immunol. (2013) 13:176–89. 10.1038/nri3401 23411799

[195] Seite JF Shoenfeld Y Youinou P Hillion S. What is the contents of the magic draft IVIg? Autoimmun Rev. (2008) 7:435–9. 10.1016/j.autrev.2008.04.012 18558358

[196] Nimmerjahn F Ravetch JV. The antiinflammatory activity of IgG: the intravenous IgG paradox. J Exp Med. (2007) 204:11–5. 10.1084/jem.20061788 17227911 PMC2118416

[197] Beck A Goetsch L Dumontet C Corvaia N. Strategies and challenges for the next generation of antibody-drug conjugates. Nat Rev Drug Discov. (2017) 16:315–37. 10.1038/nrd.2016.268 28303026

[198] Sievers EL Senter PD. Antibody-drug conjugates in cancer therapy. Annu Rev Med. (2013) 64:15–29. 10.1146/annurev-med-050311-201823 23043493

[199] Perez HL Cardarelli PM Deshpande S Gangwar S Schroeder GM Vite GD. Antibody-drug conjugates: current status and future directions. Drug Discov Today (2014) 19:869–81. 10.1016/j.drudis.2013.11.004 24239727

[200] McCombs JR Owen SC. Antibody drug conjugates: design and selection of linker, payload and conjugation chemistry. AAPS J. (2015) 17:339–51. 10.1208/s12248-014-9710-8 25604608 PMC4365093

[201] Zhou Q Stefano JE Manning C Kyazike J Chen B Gianolio DA. Site-specific antibody-drug conjugation through glycoengineering. Bioconjug Chem. (2014) 25:510–20. 10.1021/bc400505q 24533768

[202] Liu S Dicker KT Jia X Modular and orthogonal synthesis of hybrid polymers and networks. Chem Commun. (2015) 51:5218–37. 10.1039/C4CC09568E PMC4359094 25572255

[203] Du J Meledeo MA Wang Z Khanna HS Paruchuri VD Yarema KJ. Metabolic glycoengineering: sialic acid and beyond. Glycobiology (2009) 19:1382–401. 10.1093/glycob/cwp115 19675091 PMC2770326

[204] Okeley NM Toki BE Zhang X Jeffrey SC Burke PJ Alley SC. Metabolic engineering of monoclonal antibody carbohydrates for antibody-drug conjugation. Bioconjug Chem. (2013) 24:1650–55. 10.1021/bc4002695 24050213

[205] Li X Fang T Boons GJ. Preparation of well-defined antibody-drug conjugates through glycan remodeling and strain-promoted azide-alkyne cycloadditions. Angew Chem Int Ed Engl. (2014) 53:7179–82. 10.1002/anie.201402606 24862406 PMC4128391

[206] Qasba PK. Glycans of antibodies as a specific site for drug conjugation using glycosyltransferases. Bioconjug Chem. (2015) 26:2170–5. 10.1021/acs.bioconjchem.5b00173 26065635

[207] van Geel R Wijdeven MA Heesbeen R Verkade JM Wasiel AA van Berkel SS. Chemoenzymatic conjugation of toxic payloads to the globally conserved N-glycan of native mAbs provides homogeneous and highly efficacious antibody-drug conjugates. Bioconjug Chem. (2015) 26:2233–42. 10.1021/acs.bioconjchem.5b00224 26061183

[208] Salatino M Girotti MR Rabinovich GA. Glycans pave the way for immunotherapy in triple-negative breast cancer. Cancer Cell (2018) 33:155–7. 10.1016/j.ccell.2018.01.015 29438689

[209] Brekke OH Sandlie I. Therapeutic antibodies for human diseases at the dawn of the twenty-first century. Nat Rev Drug Discov. (2003) 2:52–62. 10.1038/nrd984 12509759

[210] Ma H O'Kennedy R. The structure of natural and recombinant antibodies. Methods Mol Biol. (2015) 1348:7–11. 10.1007/978-1-4939-2999-3_2 26424258

[211] Stanfield RL Wilson IA Antibody structure. Microbiol Spectr. (2014). 10.1128/microbiolspec.AID-0012-2013. [Epub ahead of print]. 26105818

[212] Siontorou CG. Nanobodies as novel agents for disease diagnosis and therapy. Int J Nanomed. (2013) 8:4215–27. 10.2147/IJN.S39428 24204148 PMC3818023

[213] Hamers-Casterman C Atarhouch T Muyldermans S Robinson G Hamers C. Naturally occurring antibodies devoid of light chains. Nature (1993) 363:446–8. 10.1038/363446a0 8502296

[214] Saerens D Ghassabeh GH Muyldermans S. Single-domain antibodies as building blocks for novel therapeutics. Curr Opin Pharmacol. (2008) 8:600–8. 10.1016/j.coph.2008.07.006 18691671

[215] Wesolowski J Alzogaray V Reyelt J Unger M Juarez K Urrutia M. Single domain antibodies: promising experimental and therapeutic tools in infection and immunity. Med Microbiol Immunol. (2009) 198:157–74. 10.1007/s00430-009-0116-7 19529959 PMC2714450

[216] Holliger P Hudson PJ. Engineered antibody fragments and the rise of single domains. Nat Biotechnol. (2005) 23:1126–36. 10.1038/nbt1142 16151406

[217] Wang M Lee LS Nepomich A Yang JD Conover C Whitlow M. Single-chain Fv with manifold N-glycans as bifunctional scaffolds for immunomolecules. Protein Eng. (1998) 11:1277–83. 10.1093/protein/11.12.1277 9930678

[218] Harmsen MM van Solt CB Fijten HP. Enhancement of toxin- and virus-neutralizing capacity of single-domain antibody fragments by N-glycosylation. Appl Microbiol Biotechnol. (2009) 84:1087–94. 10.1007/s00253-009-2029-1 19455325 PMC2755796

[219] Jenkins N Curling EM. Glycosylation of recombinant proteins: problems and prospects. Enzyme Microb Technol. (1994) 16:354–64. 10.1016/0141-0229(94)90149-X 7764790

[220] De Andrea M Ravera R Gioia D Gariglio M Landolfo S. The interferon system: an overview. Eur J Paediatr Neurol. (2002) 6(Suppl A):8. 10.1053/ejpn.2002.0573 12365360

[221] Goldenberg MM. Multiple sclerosis review. PT (2012) 37:175–84. 22605909 PMC3351877

[222] Freedman MS. Long-term follow-up of clinical trials of multiple sclerosis therapies. Neurology (2011) 76:26. 10.1212/WNL.0b013e318205051d 21205679

[223] Murdoch D Lyseng-Williamson KA. Spotlight on subcutaneous recombinant interferon-β-1a (Rebif) in relapsing-remitting multiple sclerosis. BioDrugs (2005) 19:323–5. 10.2165/00063030-200519050-00005 16207073

[224] Runkel L Meier W Pepinsky RB Karpusas M Whitty A Kimball K. Structural and functional differences between glycosylated and non-glycosylated forms of human interferon-β (IFN-β). Pharm Res. (1998) 15:641–9. 10.1023/A:1011974512425 9587963

[225] Song K Yoon IS Kim NA Kim DH Lee J Lee HJ. Glycoengineering of interferon-β 1a improves its biophysical and pharmacokinetic properties. PLoS ONE (2014) 9:e96967. 10.1371/journal.pone.0096967 24858932 PMC4032242

[226] Naghmeh M Amir A Elias O Bita K Modares GM Sorayya K Therapeutic effect of Avonex, Rebif and Betaferon on quality of life in multiple sclerosis. Psychiatry Clin Neurosci. (2015) 69:649–57. 10.1111/pcn.12308 25907350

[227] Christophi GP Christophi JA Gruber RC Mihai C Mejico LJ Massa PT Quantitative differences in the immunomodulatory effects of Rebif and Avonex in IFN-b 1a treated multiple sclerosis patients. J Neurol Sci. (2011) 307:41–5. 10.1016/j.jns.2011.05.024 21658727 PMC3395433

[228] Jiang T Zhou C Ren S. Role of IL-2 in cancer immunotherapy. Oncoimmunology (2016) 5:e1163462. 10.1080/2162402X.2016.1163462 27471638 PMC4938354

[229] Rosenberg SA Lotze MT Muul LM Leitman S Chang AE Ettinghausen SE. Observations on the systemic administration of autologous lymphokine-activated killer cells and recombinant interleukin-2 to patients with metastatic cancer. N Engl J Med. (1985) 313:1485–92. 10.1056/NEJM198512053132327 3903508

[230] Rosenberg SA. IL-2: the first effective immunotherapy for human cancer. J Immunol. (2014) 192:5451–8. 10.4049/jimmunol.1490019 24907378 PMC6293462

[231] Lam ET Wong MK Agarwal N Redman BG Logan T Gao D. Retrospective analysis of the safety and efficacy of high-dose interleukin-2 after prior tyrosine kinase inhibitor therapy in patients with advanced renal cell carcinoma. J Immunother. (2014) 37:360–5. 10.1097/CJI.0000000000000044 25075565 PMC4127096

[232] Gearing AJ Thorpe R. The international standard for human interleukin-2. Calibration by international collaborative study. J Immunol Methods (1988) 114:3–9. 10.1016/0022-1759(88)90145-7 3263444

[233] Robb RJ Kutny RM Panico M Morris HR Chowdhry V. Amino acid sequence and post-translational modification of human interleukin 2. Proc Natl Acad Sci USA. (1984) 81:6486–90. 10.1073/pnas.81.20.6486 6333684 PMC391949

[234] Wadhwa M Bird C Heath AB Dilger P Thorpe R Participants of the collaborative study The 2nd International Standard for Interleukin-2 (IL-2). Report of a collaborative study. J Immunol Methods (2013) 397:1–7. 10.1016/j.jim.2013.07.012 23948423

[235] Kamionka M. Engineering of therapeutic proteins production in *Escherichia coli*. Curr Pharm Biotechnol. (2011) 12:268–74. 10.2174/138920111794295693 21050165 PMC3179032

[236] Metcalf D. The colony-stimulating factors and cancer. Cancer Immunol Res. (2013) 1:351–6. 10.1158/2326-6066.CIR-13-0151 24524092 PMC3918448

[237] Crawford J Glaspy JA Stoller RG Tomita DK Vincent ME McGuire BW. Final results of a placebo-controlled study of filgrastim in small-cell lung cancer: exploration of risk factors for febrile neutropenia. Support Cancer Ther. (2005) 3:36–46. 10.3816/SCT.2005.n.023 18632435

[238] Amadori S Suciu S Jehn U Stasi R Thomas X Marie JP. Leukemia group use of glycosylated recombinant human G-CSF (lenograstim) during and/or after induction chemotherapy in patients 61 years of age and older with acute myeloid leukemia: final results of AML-13, a randomized phase-3 study. Blood (2005) 106:27–34. 10.1182/blood-2004-09-3728 15761020 PMC1895135

[239] Hussein AM Ross M Vredenburgh J Meisenberg B Hars V Gilbert C. Effects of granulocyte-macrophage colony stimulating factor produced in Chinese hamster ovary cells (regramostim), *Escherichia coli* (molgramostim) and yeast (sargramostim) on priming peripheral blood progenitor cells for use with autologous bone marrow after high-dose chemotherapy. Eur J Haematol. (1995) 55:348–56. 10.1111/j.1600-0609.1995.tb00713.x 7493686

[240] Hoglund M. Glycosylated and non-glycosylated recombinant human granulocyte colony-stimulating factor (rhG-CSF)–what is the difference? Med Oncol. (1998) 15:229–33. 10.1007/BF02787205 9951685

[241] Stern AM Markel H. The history of vaccines and immunization: familiar patterns, new challenges. Health Aff (2005) 24:611–21. 10.1377/hlthaff.24.3.611 15886151

[242] XXX Anonymous Pneumococcal Vaccination. Available online at: https://www.cdc.gov/pneumococcal/vaccination.html (Accessed July 28, 2018).

[243] Daniels CC Rogers PD Shelton CM. A review of pneumococcal vaccines: current polysaccharide vaccine recommendations and future protein antigens. J Pediatr Pharmacol Ther. (2016) 21:27–35. 10.5863/1551-6776-21.1.27 26997927 PMC4778694

[244] Costantino P Rappuoli R Berti F. The design of semi-synthetic and synthetic glycoconjugate vaccines. Exp Opin Drug Disc. (2011) 6:1045–66. 10.1517/17460441.2011.609554 22646863

[245] Scanlan CN Offer J Zitzmann N Dwek RA. Exploiting the defensive sugars of HIV-1 for drug and vaccine design. Nature (2007) 446:1038. 10.1038/nature05818 17460665

[246] Horiya S MacPherson IS Krauss IJ. Recent strategies targeting HIV glycans in vaccine design. Nat Chem Biol. (2014) 10:990–9. 10.1038/nchembio.1685 25393493 PMC4431543

[247] Wu CY Lin CW Tsai TI Lee CD Chuang HY Chen JB. Influenza A surface glycosylation and vaccine design. Proc Natl Acad Sci USA. (2017) 114:280–5. 10.1073/pnas.1617174114 28028222 PMC5240703

[248] Hutter J Rodig JV Hoper D Seeberger PH Reichl U Rapp E. Toward animal cell culture-based influenza vaccine design: viral hemagglutinin N-glycosylation markedly impacts immunogenicity. J Immunol. (2013) 190:220–30. 10.4049/jimmunol.1201060 23225881

[249] Dowling W Thompson E Badger C Mellquist JL Garrison AR Smith JM. Influences of glycosylation on antigenicity, immunogenicity, and protective efficacy of ebola virus GP DNA vaccines. J Virol. (2007) 81:1821–37. 10.1128/JVI.02098-06 17151111 PMC1797596

[250] Eshhar Z Waks T Gross G Schindler DG. Specific activation and targeting of cytotoxic lymphocytes through chimeric single chains consisting of antibody-binding domains and the γ or ζ subunits of the immunoglobulin and T-cell receptors. Proc Natl Acad Sci USA. (1993) 90:720–4. 10.1073/pnas.90.2.720 8421711 PMC45737

[251] Gross G Waks T Eshhar Z. Expression of immunoglobulin-T-cell receptor chimeric molecules as functional receptors with antibody-type specificity. Proc Natl Acad Sci USA. (1989) 86:10024–8. 10.1073/pnas.86.24.10024 2513569 PMC298636

[252] Wilkins O Keeler AM Flotte TR. CAR T-cell therapy: progress and prospects. Hum Gene Ther Meth. (2017) 28:61–6. 10.1089/hgtb.2016.153 28330372 PMC5429042

[253] Figueroa JA Reidy A Mirandola L Trotter K Suvorava N Figueroa A. Chimeric antigen receptor engineering: a right step in the evolution of adoptive cellular immunotherapy. Int Rev Immunol. (2015) 34:154–87. 10.3109/08830185.2015.1018419 25901860

[254] Zhang C Liu J Zhong JF Zhang X. Engineering CAR-T cells. Biomark Res. (2017) 5:22. eCollection 2017. 10.1186/s40364-017-0102-y 28652918 PMC5482931

[255] Levine BL Miskin J Wonnacott K Keir C. Global manufacturing of CAR T cell therapy. Mol Ther Methods Clin Dev. (2016) 4:92–101. 10.1016/j.omtm.2016.12.006 28344995 PMC5363291

[256] Steentoft C Migliorini D King TR Mandel U June CH Posey AD. Glycan-directed Car-T cells. Glycobiology (2018) 28:656–69. 10.1093/glycob/cwy008 29370379

[257] Blidner AG Marino KV Rabinovich GA. Driving CARs into sweet roads: targeting glycosylated antigens in cancer. Immunity (2016) 44:1248–50. 10.1016/j.immuni.2016.06.010 27332727

[258] Hege KM Bergsland EK Fisher GA Nemunaitis JJ Warren RS McArthur JG. Safety, tumor trafficking and immunogenicity of chimeric antigen receptor (CAR)-T cells specific for TAG-72 in colorectal cancer. J Immunother Cancer (2017) 5:9. eCollection 2017. 10.1186/s40425-017-0222-9 28344808 PMC5360066

[259] Westwood JA Smyth MJ Teng MW Moeller M Trapani JA Scott AM. Adoptive transfer of T cells modified with a humanized chimeric receptor gene inhibits growth of Lewis-Y-expressing tumors in mice. Proc Natl Acad Sci USA. (2005) 102:19051–6. 10.1073/pnas.0504312102 16365285 PMC1323148

[260] Ritchie DS Neeson PJ Khot A Peinert S Tai T Tainton K. Persistence and efficacy of second generation CAR T cell against the LeY antigen in acute myeloid leukemia. Mol Ther. (2013) 21:2122–9. 10.1038/mt.2013.154 23831595 PMC3831035

[261] Louis CU Savoldo B Dotti G Pule M Yvon E Myers GD. Antitumor activity and long-term fate of chimeric antigen receptor-positive T cells in patients with neuroblastoma. Blood (2011) 118:6050–6. 10.1182/blood-2011-05-354449 21984804 PMC3234664

[262] Pule MA Savoldo B Myers GD Rossig C Russell HV Dotti G. Virus-specific T cells engineered to coexpress tumor-specific receptors: persistence and antitumor activity in individuals with neuroblastoma. Nat Med. (2008) 14:1264–70. 10.1038/nm.1882 18978797 PMC2749734

[263] Heczey A Louis CU Savoldo B Dakhova O Durett A Grilley B. CAR T cells administered in combination with lymphodepletion and PD-1 inhibition to patients with neuroblastoma. Mol Ther. (2017) 25:2214–24. 10.1016/j.ymthe.2017.05.012 28602436 PMC5589058

[264] Lavrsen K Madsen CB Rasch MG Woetmann A Odum N Mandel U. Aberrantly glycosylated MUC1 is expressed on the surface of breast cancer cells and a target for antibody-dependent cell-mediated cytotoxicity. Glycoconj J. (2013) 30:227–36. 10.1007/s10719-012-9437-7 22878593

[265] Hoogduijn MJ Popp F Verbeek R Masoodi M Nicolaou A Baan C. The immunomodulatory properties of mesenchymal stem cells and their use for immunotherapy. Int Immunopharmacol. (2010) 10:1496–500. 10.1016/j.intimp.2010.06.019 20619384

[266] Luk F de Witte SF Bramer WM Baan CC Hoogduijn MJ. Efficacy of immunotherapy with mesenchymal stem cells in man: a systematic review. Expert Rev Clin Immunol. (2015) 11:617–36. 10.1586/1744666X.2015.1029458 25817052

[267] De Becker A Riet IV. Homing and migration of mesenchymal stromal cells: how to improve the efficacy of cell therapy? World J Stem Cells (2016) 8:73–87. 10.4252/wjsc.v8.i3.73 27022438 PMC4807311

[268] Sackstein R. Glycosyltransferase-programmed stereosubstitution (GPS) to create HCELL: engineering a roadmap for cell migration. Immunol Rev. (2009) 230:51–74. 10.1111/j.1600-065X.2009.00792.x 19594629 PMC4306344

[269] Yi T Song SU. Immunomodulatory properties of mesenchymal stem cells and their therapeutic applications. Arch Pharm Res. (2012) 35:213–21. 10.1007/s12272-012-0202-z 22370776

[270] Sackstein R Merzaban JS Cain DW Dagia NM Spencer JA Lin CP. *Ex vivo* glycan engineering of CD44 programs human multipotent mesenchymal stromal cell trafficking to bone. Nat Med. (2008) 14:181–7. 10.1038/nm1703 18193058

[271] Warren L Manos PD Ahfeldt T Loh YH Li H Lau F. Highly efficient reprogramming to pluripotency and directed differentiation of human cells with synthetic modified mRNA. Cell Stem Cell (2010) 7:618–30. 10.1016/j.stem.2010.08.012 20888316 PMC3656821

[272] Levy O Zhao W Mortensen LJ Leblanc S Tsang K Fu M. mRNA-engineered mesenchymal stem cells for targeted delivery of interleukin-10 to sites of inflammation. Blood (2013) 122:23. 10.1182/blood-2013-04-495119 23980067 PMC3790516

[273] Dykstra B Lee J Mortensen LJ Yu H Wu ZL Lin CP. Glycoengineering of E-selectin ligands by intracellular versus extracellular fucosylation differentially affects osteotropism of human mesenchymal stem cells. Stem Cells (2016) 34:2501–11. 10.1002/stem.2435 27335219 PMC5064874

[274] Thankamony SP Sackstein R. Enforced hematopoietic cell E- and L-selectin ligand (HCELL) expression primes transendothelial migration of human mesenchymal stem cells. Proc Natl Acad Sci USA. (2011) 108:2258–63. 10.1073/pnas.1018064108 21257905 PMC3038774

[275] Merzaban JS Burdick MM Gadhoum SZ Dagia NM Chu JT Fuhlbrigge RC. Analysis of glycoprotein E-selectin ligands on human and mouse marrow cells enriched for hematopoietic stem/progenitor cells. Blood (2011) 118:1774–83. 10.1182/blood-2010-11-320705 21659548 PMC3158712

[276] Merzaban JS Imitola J Starossom SC Zhu B Wang Y Lee J. Cell surface glycan engineering of neural stem cells augments neurotropism and improves recovery in a murine model of multiple sclerosis. Glycobiology (2015) 25:1392–409. 10.1093/glycob/cwv046 26153105 PMC4634313

[277] Parmar S Liu X Najjar A Shah N Yang H Yvon E. *Ex vivo* fucosylation of third-party human regulatory T cells enhances anti-graft-versus-host disease potency *in vivo*. Blood (2015) 125:1502–6. 10.1182/blood-2014-10-603449 25428215 PMC4342362

[278] Garay RP El-Gewely R Armstrong JK Garratty G Richette P. Antibodies against polyethylene glycol in healthy subjects and in patients treated with PEG-conjugated agents. Exp Opin Drug Deliv. (2012) 9:1319–23. 10.1517/17425247.2012.720969 22931049

[279] Schellekens H Hennink WE Brinks V. The immunogenicity of polyethylene glycol: facts and fiction. Pharm Res. (2013) 30:1729–34. 10.1007/s11095-013-1067-7 23673554

[280] Liu Y Reidler H Pan J Milunic D Qin D Chen D. A double antigen bridging immunogenicity ELISA for the detection of antibodies to polyethylene glycol polymers. J Pharmacol Toxicol Methods (2011) 64:238–45. 10.1016/j.vascn.2011.07.003 21827863

[281] Anonymous Guidance for Industry on Immunogenicity Assessment for Therapeutic Protein Products. U.S. Department of Health and Human Services Food and Drug Administration, Washington DC (2014).

[282] Ratanji KD Derrick JP Dearman RJ Kimber I. Immunogenicity of therapeutic proteins: influence of aggregation. J Immunotoxicol. (2014) 11:99–109. 10.3109/1547691X.2013.821564 23919460 PMC4002659

[283] Oh-eda M Hasegawa M Hattori K Kuboniwa H Kojima T Orita T. O-linked sugar chain of human granulocyte colony-stimulating factor protects it against polymerization and denaturation allowing it to retain its biological activity. J Biol Chem. (1990) 265:11432–5. 1694845

[284] Sola RJ Griebenow K. Effects of glycosylation on the stability of protein pharmaceuticals. J Pharm Sci. (2009) 98:1223–45. 10.1002/jps.21504 18661536 PMC2649977

[285] van Beers MM Bardor M. Minimizing immunogenicity of biopharmaceuticals by controlling critical quality attributes of proteins. Biotechnol J. (2012) 7:1473–84. 10.1002/biot.201200065 23027660

[286] Hoiberg-Nielsen R Westh P Arleth L. The effect of glycosylation on interparticle interactions and dimensions of native and denatured phytase. Biophys J. (2009) 96:153–61. 10.1529/biophysj.108.136408 18835893 PMC2710053

[287] Zheng K Bantog C Bayer R. The impact of glycosylation on monoclonal antibody conformation and stability. MAbs (2011) 3:568–76. 10.4161/mabs.3.6.17922 22123061 PMC3242843

[288] Sola RJ Rodriguez-Martinez JA Griebenow K. Modulation of protein biophysical properties by chemical glycosylation: biochemical insights and biomedical implications. Cell Mol Life Sci. (2007) 64:2133–52. 10.1007/s00018-007-6551-y 17558468 PMC11138447

[289] Li W Zhu Z Chen W Feng Y Dimitrov DS. Crystallizable fragment glycoengineering for therapeutic antibodies development. Front Immunol. (2017) 8:1554. 10.3389/fimmu.2017.01554 29181010 PMC5693878

[290] Sola RJ Al-Azzam W Griebenow K. Engineering of protein thermodynamic, kinetic, and colloidal stability: chemical glycosylation with monofunctionally activated glycans. Biotechnol Bioeng. (2006) 94:1072–9. 10.1002/bit.20933 16586505

[291] Imperiali B Protein glycosylation: the clash of the titans. Acc Chem Res. (1997) 30:452–9.

[292] Bosques CJ Tschampel SM Woods RJ Imperiali B. Effects of glycosylation on peptide conformation: a synergistic experimental and computational study. J Am Chem Soc. (2004) 126:8421–5. 10.1021/ja0496266 15237998 PMC1386730

[293] Petrescu A Milac A Petrescu SM Dwek RA Wormald MR. Statistical analysis of the protein environment of N-glycosylation sites: implications for occupancy, structure, and folding. Glycobiology (2004) 14:103–14. 10.1093/glycob/cwh008 14514716

[294] Joao HC Scragg IG Dwek RA. Effects of glycosylation on protein conformation and amide proton exchange rates in RNase B. FEBS Lett. (1992) 307:343–6. 10.1016/0014-5793(92)80709-P 1322837

[295] Martínek V Sklenár J Dračínský M Šulc M Hofbauerová K Bezouška K. Glycosylation protects proteins against free radicals generated from toxic xenobiotics. Toxicol Sci. (2010) 117:359–74. 10.1093/toxsci/kfq206 20616208

[296] Uchida E Morimoto K Kawasaki N Izaki Y Abdu Said A Hayakawa T Effect of active oxygen radicals on protein and carbohydrate moieties of recombinant human erythropoietin. Free Radic Res. (1997) 27:311–23. 10.3109/10715769709065769 9350435

[297] Folzer E Diepold K Bomans K Finkler C Schmidt R Bulau P. Selective oxidation of methionine and tryptophan residues in a therapeutic IgG1 molecule. J Pharm Sci. (2015) 104:2824–31. 10.1002/jps.24509 26010344

[298] Lam XM Yang JY Cleland JL. Antioxidants for prevention of methionine oxidation in recombinant monoclonal antibody HER2. J Pharm Sci. (1997) 86:1250–5. 10.1021/js970143s 9383735

[299] Hermeling S Aranha L Damen JM Slijper M Schellekens H Crommelin DJ Structural characterization and immunogenicity in wild-type and immune tolerant mice of degraded recombinant human interferon a2b. Pharm Res. (2005) 22:1997–2006. 10.1007/s11095-005-8177-9 16184451

[300] van Beers MM Sauerborn M Gilli F Brinks V Schellekens H Jiskoot W. Oxidized and aggregated recombinant human interferon β is immunogenic in human interferon β transgenic mice. Pharm Res. (2011) 28:2393–402. 10.1007/s11095-011-0451-4 21544687 PMC3170469

[301] Kuriakose A Chirmule N Nair P. Immunogenicity of biotherapeutics: causes and association with posttranslational modifications. J Immunol Res. (2016) 2016:1298473. 10.1155/2016/1298473 27437405 PMC4942633

[302] Plotkin SA Plotkin SL. The development of vaccines: how the past led to the future. Nat Rev Microbiol. (2011) 9:889–93. 10.1038/nrmicro2668 21963800

[303] Baeshen NA Baeshen MN Sheikh A Bora RS Ahmed MM Ramadan HA. Cell factories for insulin production. Microb Cell Fact. (2014) 13:141. 10.1186/s12934-014-0141-0 25270715 PMC4203937

[304] Hossler P Khattak SF Li ZJ. Optimal and consistent protein glycosylation in mammalian cell culture. Glycobiology (2009) 19:936–49. 10.1093/glycob/cwp079 19494347

[305] Ladisch MR Kohlmann KL. Recombinant human insulin. Biotechnol Prog. (1992) 8:469–78. 10.1021/bp00018a001 1369033

[306] Helenius A Aebi M. Intracellular functions of N-linked glycans. Science (2001) 291:2364–9. 10.1126/science.291.5512.2364 11269317

[307] Nielsen KH. Protein expression-yeast. Methods Enzymol (2014) 536:133–47. 10.1016/B978-0-12-420070-8.00012-X 24423273

[308] Ahmad M Hirz M Pichler H Schwab H. Protein expression in *Pichia pastoris*: recent achievements and perspectives for heterologous protein production. Appl Microbiol Biotechnol. (2014) 98:5301–17. 10.1007/s00253-014-5732-5 24743983 PMC4047484

[309] Meehl MA Stadheim TA. Biopharmaceutical discovery and production in yeast. Curr Opin Biotechnol. (2014) 30:120–7. 10.1016/j.copbio.2014.06.007 25014890

[310] Lawrence SM Huddleston KA Tomiya N Nguyen N Lee YC Vann WF. Cloning and expression of human sialic acid pathway genes to generate CMP-sialic acids in insect cells. Glycoconj J. (2001) 18:205–13. 10.1023/A:1012452705349 11602804

[311] Tomiya N Howe D Aumiller JJ Pathak M Park J Palter KB Complex-type biantennary N-glycans of recombinant human transferrin from Trichoplusia in insect cells expressing mammalian [β]-1,4-galactosyltransferase and [β]-1,2-N-acetylglucosaminyltransferase II. Glycobiology (2003) 13:23–34. 10.1093/glycob/cwg012 12634321

[312] Viswanathan K Lawrence S Hinderlich S Yarema KJ Lee YC Betenbaugh MJ. Engineering sialic acid synthetic ability into insect cells: identifying metabolic bottlenecks and devising strategies to overcome them. Biochemistry (2003) 42:15215–25. 10.1021/bi034994s 14690432

[313] Granell AE Palter KB Akan I Aich U Yarema KJ Betenbaugh MJ. DmSAS is required for sialic acid biosynthesis in cultured Drosophila third instar larvae CNS neurons. ACS Chem Biol. (2011) 6:1287–95. 10.1021/cb200238k 21919466 PMC7673583

[314] Scherer WF Syverton JT Gey GO. Studies on the propagation in vitro of poliomyelitis viruses. IV. Viral multiplication in a stable strain of human malignant epithelial cells (strain HeLa) derived from an epidermoid carcinoma of the cervix. J Exp Med. (1953) 97:695–710. 10.1084/jem.97.5.695 13052828 PMC2136303

[315] Petricciani J Sheets R. An overview of animal cell substrates for biological products. Biologicals (2008) 36:359–62. 10.1016/j.biologicals.2008.06.004 18674929

[316] Butler M Spearman M. The choice of mammalian cell host and possibilities for glycosylation engineering. Curr Opin Biotechnol. (2014) 30:107–12. 10.1016/j.copbio.2014.06.010 25005678

[317] Galfre G Milstein C. Preparation of monoclonal antibodies: strategies and procedures. Meth Enzymol. (1981) 73:3–46. 10.1016/0076-6879(81)73054-4 7300683

[318] Potter M Boyce CR. Induction of plasma-cell neoplasms in strain BALB/c mice with mineral oil and mineral oil adjuvants. Nature (1962) 193:1086–7. 10.1038/1931086a0 14488296

[319] Barnes LM Bentley CM Dickson AJ. Advances in animal cell recombinant protein production: GS-NS0 expression system. Cytotechnology (2000) 32:109–23. 10.1023/A:1008170710003 19002973 PMC3449689

[320] Ghaderi D Taylor RE Padler-Karavani V Diaz S Varki A. Implications of the presence of N-glycolylneuraminic acid in recombinant therapeutic glycoproteins. Nat Biotechnol. (2010) 28:863–7. 10.1038/nbt.1651 20657583 PMC3077421

[321] Tangvoranuntakul P Gagneux P Diaz S Bardor M Varki N Varki A. Human uptake and incorporation of an immunogenic nonhuman dietary sialic acid. Proc Natl Acad Sci USA. (2003) 100:12045–50. 10.1073/pnas.2131556100 14523234 PMC218710

[322] Kim JY Kim YG Lee GM. CHO cells in biotechnology for production of recombinant proteins: current state and further potential. Appl Microbiol Biotechnol. (2012) 93:917–30. 10.1007/s00253-011-3758-5 22159888

[323] Durocher Y Butler M. Expression systems for therapeutic glycoprotein production. Curr Opin Biotechnol. (2009) 20:700–7. 10.1016/j.copbio.2009.10.008 19889531

[324] Swiech K Picanco-Castro V Covas DT. Human cells: new platform for recombinant therapeutic protein production. Protein Expr Purif. (2012) 84:147–53. 10.1016/j.pep.2012.04.023 22580292

[325] Berting A Farcet MR Kreil TR. Virus susceptibility of Chinese hamster ovary (CHO) cells and detection of viral contaminations by adventitious agent testing. Biotechnol Bioeng. (2010) 106:598–607. 10.1002/bit.22723 20503298 PMC7161873

[326] Xu X Nagarajan H Lewis NE Pan S Cai Z Liu X. The genomic sequence of the Chinese hamster ovary (CHO)-K1 cell line. Nat Biotechnol. (2011) 29:735–41. 10.1038/nbt.1932 21804562 PMC3164356

[327] Lai T Yang Y Ng SK. Advances in mammalian cell line development technologies for recombinant protein production. Pharmaceuticals (2013) 6:579–603. 10.3390/ph6050579 24276168 PMC3817724

[328] Bosques CJ Collins BE Meador JW III Sarvaiya H Murphy JL Dellorusso G. Chinese hamster ovary cells can produce galactose-a-1,3-galactose antigens on proteins. Nat Biotechnol. (2010) 28:1153–6. 10.1038/nbt1110-1153 21057479 PMC4005363

[329] Howard DR Fukuda M Fukuda MN Stanley P. The GDP-fucose:N-acetylglucosaminide 3-α-L-fucosyltransferases of LEC11 and LEC12 Chinese hamster ovary mutants exhibit novel specificities for glycolipid substrates. J Biol Chem. (1987) 262:16830–7. 2890642

[330] Sasaki H Bothner B Dell A Fukuda M. Carbohydrate structure of erythropoietin expressed in Chinese hamster ovary cells by a human erythropoietin cDNA. J Biol Chem. (1987) 262:12059–76. 3624248

[331] Campbell C Stanley P. A dominant mutation to ricin resistance in Chinese hamster ovary cells induces UDP-GlcNAc:glycopeptide β-4-N-acetylglucosaminyltransferase III activity. J Biol Chem. (1984) 259:13370–8. 6238035

[332] Patnaik SK Stanley P. Lectin-resistant CHO glycosylation mutants. Meth Enzymol. (2006) 416:159–82. 10.1016/S0076-6879(06)16011-5 17113866

[333] Sinclair AM Elliott S. Glycoengineering: the effect of glycosylation on the properties of therapeutic proteins. J Pharm Sci. (2005) 94:1626–35. 10.1002/jps.20319 15959882

[334] Clausen H Wandall H Steentoft C Stanley P Schanaar R Chapter 56: Glycosylation engineering. In: Varki A Cummings R Esko J Stanley P Hart G Aebi M Darvill A Kinoshita T Packer N Prestegard J Schnaar R Seeberger P, editors. Essentials of Glycobiology. Cold Springs Harbor, NY: Cold Springs Harbor Laboratory Press (2017).

[335] Hamilton SR Zha D. Progress in yeast glycosylation engineering. Meth Mol Biol. (2015) 1321: 73–90. 10.1007/978-1-4939-2760-9_6 26082216

[336] Castilho A Steinkellner H. Glyco-engineering in plants to produce human-like N-glycan structures. Biotechnol J. (2012) 7:1088–98. 10.1002/biot.201200032 22890723

[337] Geisler C Mabashi-Asazuma H Jarvis DL. An overview and history of glyco-engineering in insect expression systems. Meth Mol Biol. (2015) 1321:131–152. 10.1007/978-1-4939-2760-9_10 26082220

[338] Chandrasegaran S Carroll D. Origins of programmable nucleases for genome engineering. J Mol Biol. (2016) 428:963–89. 10.1016/j.jmb.2015.10.014 26506267 PMC4798875

[339] Wang Q Yin B Chung CY Betenbaugh MJ. Glycoengineering of CHO cells to improve product quality. Meth Mol Biol. (2017) 1603:25–44. 10.1007/978-1-4939-6972-2_2 28493121

[340] Agrawal P Kurcon T Pilobello KT Rakus JF Koppolu S Liu Z. Mapping posttranscriptional regulation of the human glycome uncovers microRNA defining the glycocode. Proc Natl Acad Sci USA. (2014) 111:4338–43. 10.1073/pnas.1321524111 24591635 PMC3964104

[341] Lee EU Roth J Paulson JC. Alteration of terminal glycosylation sequences on N-linked oligosaccharides of Chinese hamster ovary cells by expression of β-galactoside α 2,6-sialyltransferase. J Biol Chem. (1989) 264:13848–55. 2668274

[342] Minch SL Kallio PT Bailey JE. Tissue plasminogen activator coexpressed in Chinese hamster ovary cells with α(2,6)-sialyltransferase contains NeuAc α(2,6)Gal β(1,4)Glc-N-AcR linkages. Biotechnol Prog. (1995) 11:348–51. 10.1021/bp00033a015 7619404

[343] Schlenke P Grabenhorst E Wagner R Nimtz M Conradt HS Expression of human α2:6-sialyltransferase in BHK-21A cells increases the sialylation of coexpressed human erythropoietin: NeuAc-transfer onto GalNAc(βl-4)GlcNAc-R motives. In: Carrondo MJT Griffiths B Moreira JLP, editors. Animal Cell Technology: From Vaccines to Genetic Medicine. Dordrecht: Springer Netherlands (1997). p. 475–80.

[344] Jeong YT Choi O Lim HR Son YD Kim HJ Kim JH. Enhanced sialylation of recombinant erythropoietin in CHO cells by human glycosyltransferase expression. J Microbiol Biotechnol. (2008) 18:1945–1952. 10.4014/jmb.0800.546 19131698

[345] Yin B Gao Y Chung CY Yang S Blake E Stuczynski MC. Glycoengineering of Chinese hamster ovary cells for enhanced erythropoietin N-glycan branching and sialylation. Biotechnol Bioeng. (2015) 112:2343–51. 10.1002/bit.25650 26154505

[346] Weikert S Papac D Briggs J Cowfer D Tom S Gawlitzek M. Engineering Chinese hamster ovary cells to maximize sialic acid content of recombinant glycoproteins. Nat Biotechnol. (1999) 17:1116–21. 10.1038/15104 10545921

[347] Bragonzi A Distefano G Buckberry LD Acerbis G Foglieni C Lamotte D. A new Chinese hamster ovary cell line expressing α2,6-sialyltransferase used as universal host for the production of human-like sialylated recombinant glycoproteins. Biochim Biophys Acta (2000) 1474:273–82. 10.1016/S0304-4165(00)00023-4 10779678

[348] Monaco L Marc A Eon-Duval A Acerbis G Distefano G Lamotte D. Genetic engineering of α2,6-sialyltransferase in recombinant CHO cells and its effects on the sialylation of recombinant interferon-γ. Cytotechnology (1996) 22:197–203. 10.1007/BF00353939 22358930

[349] Jassal R Jenkins N Charlwood J Camilleri P Jefferis R Lund J. Sialylation of human IgG-Fc carbohydrate by transfected rat α2,6-sialyltransferase. Biochem Biophys Res Commun. (2001) 286:243–9. 10.1006/bbrc.2001.5382 11500028

[350] Raymond C Robotham A Spearman M Butler M Kelly J Durocher Y. Production of α2,6-sialylated IgG1 in CHO cells. MAbs (2015) 7:571–83. 10.1080/19420862.2015.1029215 25875452 PMC4622614

[351] Cha HM Lim JH Yeon JH Hwang JM Kim DI. Co-overexpression of Mgat1 and Mgat4 in CHO cells for production of highly sialylated albumin-erythropoietin. Enzyme Microb Technol. (2017) 103:53–8. 10.1016/j.enzmictec.2017.04.010 28554385

[352] Fukuta K Yokomatsu T Abe R Asanagi M Makino T. Genetic engineering of CHO cells producing human interferon-γ by transfection of sialyltransferases. Glycoconj J. (2000) 17:895–904. 10.1023/A:1010977431061 11511814

[353] Fukuta K Abe R Yokomatsu T Kono N Asanagi M Omae F. Remodeling of sugar chain structures of human interferon-γ. Glycobiology (2000) 10:421–30. 10.1093/glycob/10.4.421 10764830

[354] Seppala R Lehto VP Gahl WA. Mutations in the human UDP-N-acetylglucosamine 2-epimerase gene define the disease sialuria and the allosteric site of the enzyme. Am J Hum Genet. (1999) 64:1563–9. 10.1086/302411 10330343 PMC1377899

[355] Yarema KJ Goon S Bertozzi CR. Metabolic selection of glycosylation defects in human cells. Nat Biotechnol. (2001) 19:553–8. 10.1038/89305 11385460

[356] Hinderlich S Weidemann W Yardeni T Horstkorte R Huizing M. UDP-GlcNAc 2-Epimerase/ManNAc Kinase (GNE): a master regulator of sialic acid synthesis. Top Curr Chem. (2015) 366:97–137. 10.1007/128_2013_464 23842869 PMC4161665

[357] Bork K Reutter W Weidemann W Horstkorte R. Enhanced sialylation of EPO by overexpression of UDP-GlcNAc 2-epimerase/ManAc kinase containing a sialuria mutation in CHO cells. FEBS Lett. (2007) 581:4195–8. 10.1016/j.febslet.2007.07.060 17706199

[358] Son YD Jeong YT Park SY Kim JH. Enhanced sialylation of recombinant human erythropoietin in Chinese hamster ovary cells by combinatorial engineering of selected genes. Glycobiology (2011) 21:1019–28. 10.1093/glycob/cwr034 21436238

[359] Wong NS Yap MG Wang DI. Enhancing recombinant glycoprotein sialylation through CMP-sialic acid transporter over expression in Chinese hamster ovary cells. Biotechnol Bioeng. (2006) 93:1005–16. 10.1002/bit.20815 16432895

[360] Monti E Miyagi T. Structure and function of mammalian sialidases. Top Curr Chem. (2015) 366:183–208. 10.1007/128_2012_328 22760823

[361] Zhang M Koskie K Ross JS Kayser KJ Caple MV. Enhancing glycoprotein sialylation by targeted gene silencing in mammalian cells. Biotechnol Bioeng. (2010) 105:1094–105. 10.1002/bit.22633 20014139

[362] Smith RE Jr Jaiyesimi IA Meza LA Tchekmedyian NS Chan D Griffith H. Novel erythropoiesis stimulating protein (NESP) for the treatment of anaemia of chronic disease associated with cancer. Br J Cancer (2001) 84:24. 10.1054/bjoc.2001.1749 11308271 PMC2363901

[363] Bello NA Lewis EF Desai AS Anand IS Krum H McMurray JJ. Increased risk of stroke with darbepoetin alfa in anaemic heart failure patients with diabetes and chronic kidney disease. Eur J Heart Fail (2015) 17:1201–7. 10.1002/ejhf.412 26423928 PMC4703474

[364] Song R Oren DA Franco D Seaman MS Ho DD. Strategic addition of an N-linked glycan to a monoclonal antibody improves its HIV-1-neutralizing activity. Nat Biotechnol. (2013) 31:1047–52. 10.1038/nbt.2677 24097413 PMC3825789

[365] Campbell CT Sampathkumar SG Yarema KJ. Metabolic oligosaccharide engineering: perspectives, applications, and future directions. Mol Biosyst. (2007) 3:187–94. 10.1039/b614939c 17308665

[366] Kayser H Zeitler R Kannicht C Grunow D Nuck R Reutter W. Biosynthesis of a nonphysiological sialic acid in different rat organs, using N-propanoyl-D-hexosamines as precursors. J Biol Chem. (1992) 267:16934–8. 1512235

[367] Yorke S The application of N-acetylmannosamine to the mammalian cell culture production of recombinant human glycoproteins. Chem N Zeal. (2013) 77:18–20. Available online at: https://nzic.org.nz/app/uploads/2018/06/CiNZ-Jan-2013-min.pdf

[368] Baker KN Rendall MH Hills AE Hoare M Freedman RB James DC. Metabolic control of recombinant protein N-glycan processing in NS0 and CHO cells. Biotechnol Bioeng (2001) 73:188–202. 10.1002/bit.1051 11257601

[369] Wong NS Wati L Nissom PM Feng HT Lee MM Yap MG. An investigation of intracellular glycosylation activities in CHO cells: effects of nucleotide sugar precursor feeding. Biotechnol Bioeng (2010) 107:321–36. 10.1002/bit.22812 20506284

[370] Gu X Wang DI. Improvement of interferon-γ sialylation in Chinese hamster ovary cell culture by feeding of N-acetylmannosamine. Biotechnol Bioeng (1998) 58:642–8. 10.1002/(SICI)1097-0290(19980620)58:6<642::AID-BIT10>3.0.CO;2-9 10099302

[371] Schwartz EL Hadfield AF Brown AE Sartorelli AC. Modification of sialic acid metabolism of murine erythroleukemia cells by analogs of N-acetylmannosamine. Biochim Biophys Acta (1983) 762:489–97. 10.1016/0167-4889(83)90051-4 6871252

[372] Sarkar AK Rostand KS Jain RK Matta KL Esko JD. Fucosylation of disaccharide precursors of sialyl Lewis^x^ inhibit selectin-mediated cell adhesion. J Biol Chem. (1997) 272:25608–16. 10.1074/jbc.272.41.25608 9325281

[373] Jones MB Teng H Rhee JK Lahar N Baskaran G Yarema KJ. Characterization of the cellular uptake and metabolic conversion of acetylated N-acetylmannosamine (ManNAc) analogues to sialic acids. Biotechnol Bioeng (2004) 85:394–405. 10.1002/bit.10901 14755557

[374] Kim EJ Jones MB Rhee JK Sampathkumar SG Yarema KJ. Establishment of N-acetylmannosamine (ManNAc) analogue-resistant cell lines as improved hosts for sialic acid engineering applications. Biotechnol Prog. (2004) 20:1674–82. 10.1021/bp049841q 15575698

[375] Kim EJ Sampathkumar SG Jones MB Rhee JK Baskaran G Goon S. Characterization of the metabolic flux and apoptotic effects of O-hydroxyl- and N-acyl-modified N-acetylmannosamine analogs in Jurkat cells. J Biol Chem. (2004) 279:18342–52. 10.1074/jbc.M400205200 14966124

[376] Aich U Campbell CT Elmouelhi N Weier CA Sampathkumar SG Choi SS. Regioisomeric SCFA attachment to hexosamines separates metabolic flux from cytotoxicity and MUC1 suppression. ACS Chem Biol. (2008) 3:230–40. 10.1021/cb7002708 18338853

[377] Almaraz RT Udayanath A Khanna HS Elaine T Rahul B Shivam S. Metabolic oligosaccharide engineering with N-Acyl functionalized ManNAc analogs: cytotoxicity, metabolic flux, and glycan-display considerations. Biotechnol Bioeng (2012) 109:992–1006. 10.1002/bit.24363 22068462 PMC3288793

[378] Saeui CT Liu L Urias E Morrissette-McAlmon J Bhattacharya R Yarema KJ. Pharmacological, physiochemical, and drug-relevant biological properties of short chain fatty acid hexosamine analogues used in metabolic glycoengineering. Mol Pharm. (2018) 15:705–20. 10.1021/acs.molpharmaceut.7b00525 28853901 PMC6292510

[379] Mathew MP Tan E Shah S Bhattacharya R Adam Meledeo M Huang J. Extracellular and intracellular esterase processing of SCFA-hexosamine analogs: implications for metabolic glycoengineering and drug delivery. Bioorg Med Chem Lett. (2012) 22:6929–33. 10.1016/j.bmcl.2012.09.017 23041156 PMC3530194

[380] Yin B Wang Q Chung CY Ren X Bhattacharya R Yarema KJ. Butyrated ManNAc analog improves protein expression in Chinese hamster ovary cells. Biotechnol Bioeng (2018) 115:1531–41. 10.1002/bit.26560 29427449

[381] Yin B Wang Q Chung CY Bhattacharya R Ren X Tang J. A novel sugar analog enhances sialic acid production and biotherapeutic sialylation in CHO cells. Biotechnol Bioeng (2017) 114:1899–902. 10.1002/bit.26291 28295160

[382] Mahal LK Yarema KJ Bertozzi CR. Engineering chemical reactivity on cell surfaces through oligosaccharide biosynthesis. Science (1997) 276:1125–8. 10.1126/science.276.5315.1125 9173543

[383] Nauman DA Bertozzi CR. Kinetic parameters for small-molecule drug delivery by covalent cell surface targeting. Biochim Biophys Acta (2001) 1568:147–54. 10.1016/S0304-4165(01)00211-2 11750762

[384] Lee JH Baker TJ Mahal LK Zabner J Bertozzi CR Wiemer DF. Engineering novel cell surface receptors for virus-mediated gene transfer. J Biol Chem. (1999) 274:21878–84. 10.1074/jbc.274.31.21878 10419507

[385] Laughlin ST Baskin JM Amacher SL Bertozzi CR. *In vivo* imaging of membrane-associated glycans in developing zebrafish. Science (2008) 320:664–7. 10.1126/science.1155106 18451302 PMC2701225

[386] Hsu TL Hanson SR Kishikawa K Wang SK Sawa M Wong CH. Alkynyl sugar analogs for the labeling and visualization of glycoconjugates in cells. Proc Natl Acad Sci USA. (2007) 104:2614–9. 10.1073/pnas.0611307104 17296930 PMC1815231

[387] Tanaka Y Kohler JJ. Photoactivatable crosslinking sugars for capturing glycoprotein interactions. J Am Chem Soc. (2008) 130:3278–9. 10.1021/ja7109772 18293988

[388] Yarema KJ Sun Z. A photochemical snapshot of CD22 binding. Nat Chem Biol. (2005) 1:69–70. 10.1038/nchembio0705-69 16408000

[389] Sampathkumar SG Jones MB Yarema KJ. Metabolic expression of thiol-derivatized sialic acids on the cell surface and their quantitative estimation by flow cytometry. Nat Protoc. (2006) 1:1840–51. 10.1038/nprot.2006.252 17487167

[390] Agarwal P Bertozzi CR. Site-specific Antibody–drug conjugates: the nexus of bioorthogonal chemistry, protein engineering, and drug development. Bioconj Chem. (2015) 26:176–92. 10.1021/bc5004982 25494884 PMC4335810

[391] Zhou Q. Site-specific antibody conjugation for ADC and beyond. Biomedicines (2017) 5:64. 10.3390/biomedicines5040064 29120405 PMC5744088

[392] Sampathkumar SG Li AV Yarema KJ. Synthesis of non-natural ManNAc analogs for the expression of thiols on cell-surface sialic acids. Nat Protoc. (2006) 1:2377–85. 10.1038/nprot.2006.319 17406481

[393] Badr HA AlSadek DM El-Houseini ME Saeui CT Mathew MP Yarema KJ. Harnessing cancer cell metabolism for theranostic applications using metabolic glycoengineering of sialic acid in breast cancer as a pioneering example. Biomaterials (2017) 116:158–73. 10.1016/j.biomaterials.2016.11.044 27926828 PMC5193387

[394] Badr HA AlSadek DM Mathew MP Li CZ Djansugurova LB Yarema KJ. Nutrient-deprived cancer cells preferentially use sialic acid to maintain cell surface glycosylation. Biomaterials (2015) 70:23–36. 10.1016/j.biomaterials.2015.08.020 26295436 PMC4658327

[395] Saeui CT Nairn AV Galizzi M Douville C Gowda P Park M. Integration of genetic and metabolic features related to sialic acid metabolism distinguishes human breast cell subtypes. PLoS ONE (2018) 13:e0195812. 10.1371/journal.pone.0195812 29847599 PMC5976204

[396] Horstkorte R Rau K Laabs S Danker K Reutter W. Biochemical engineering of the N-acyl side chain of sialic acid leads to increased calcium influx from intracellular compartments and promotes differentiation of HL60 cells. FEBS Lett. (2004) 571:99–102. 10.1016/j.febslet.2004.06.067 15280024

[397] Sampathkumar SG Li AV Jones MB Sun Z Yarema KJ. Metabolic installation of thiols into sialic acid modulates adhesion and stem cell biology. Nat Chem Biol. (2006) 2:149–52. 10.1038/nchembio770 16474386

[398] Schmidt C Stehling P Schnitzer J Reutter W Horstkorte R. Biochemical engineering of neural cell surfaces by the synthetic N-propanoyl-substituted neuraminic acid precursor. J Biol Chem. (1998) 273:19146–52. 10.1074/jbc.273.30.19146 9668100

[399] Hart GW. Nutrient regulation of immunity: O-GlcNAcylation regulates stimulus-specific NF-κB–dependent transcription. Sci Signal (2013) 6:pe26. 10.1126/scisignal.2004596 23982203

[400] Bond MR Hanover JA. A little sugar goes a long way: the cell biology of O-GlcNAc. J Cell Biol. (2015) 208:869. 10.1083/jcb.201501101 25825515 PMC4384737

[401] Baudoin L Issad T. O-GlcNAcylation and inflammation: a vast territory to explore. Front Endocrinol. (2015) 5:235. 10.3389/fendo.2014.00235 25620956 PMC4288382

[402] de Jesus T Shukla S Ramakrishnan P. Too sweet to resist: control of immune cell function by O-GlcNAcylation. Cell Immunol. (2018). 10.1016/j.cellimm.2018.05.010. [Epub ahead of print]. 29887419 PMC6275141

